# Cryo-EM structural analysis of FADD:Caspase-8 complexes defines the catalytic dimer architecture for co-ordinated control of cell fate

**DOI:** 10.1038/s41467-020-20806-9

**Published:** 2021-02-05

**Authors:** Joanna L. Fox, Michelle A. Hughes, Xin Meng, Nikola A. Sarnowska, Ian R. Powley, Rebekah Jukes-Jones, David Dinsdale, Timothy J. Ragan, Louise Fairall, John W. R. Schwabe, Nobuhiro Morone, Kelvin Cain, Marion MacFarlane

**Affiliations:** 1 MRC Toxicology Unit, University of Cambridge, Hodgkin Building, Lancaster Road, Leicester, LE1 9HN UK; 2 Leicester Institute of Structural and Chemical Biology, Department of Molecular and Cellular Biology, University of Leicester, University Road, Leicester, LE1 7RH UK

**Keywords:** Proteases, Structural biology, Cell death

## Abstract

Regulated cell death is essential in development and cellular homeostasis. Multi-protein platforms, including the Death-Inducing Signaling Complex (DISC), co-ordinate cell fate via a core FADD:Caspase-8 complex and its regulatory partners, such as the cell death inhibitor c-FLIP. Here, using electron microscopy, we visualize full-length procaspase-8 in complex with FADD. Our structural analysis now reveals how the FADD-nucleated tandem death effector domain (tDED) helical filament is required to orientate the procaspase-8 catalytic domains, enabling their activation via anti-parallel dimerization. Strikingly, recruitment of c-FLIP_S_ into this complex inhibits Caspase-8 activity by altering tDED triple helix architecture, resulting in steric hindrance of the canonical tDED Type I binding site. This prevents both Caspase-8 catalytic domain assembly and tDED helical filament elongation. Our findings reveal how the plasticity, composition and architecture of the core FADD:Caspase-8 complex critically defines life/death decisions not only via the DISC, but across multiple key signaling platforms including TNF complex II, the ripoptosome, and RIPK1/RIPK3 necrosome.

## Abstract (web summary)

The core FADD:Caspase-8 complex and its regulatory partners, such as the cell death inhibitor c-FLIP, coordinate cell fate. Here authors present the structure of full-length procaspase-8 in a complex with FADD and reveal how recruitment of c-FLIP_S_ into this complex inhibits Caspase-8 activity.

## Introduction

Regulated cell death is essential in development and cellular homeostasis. Aberrant levels or inappropriate initiation of cell death can result in cancer, auto-immune disorders and neurodegenerative disease. Cell death proceeds via different pathways depending on the stimulus and the subsequent protein complexes that are formed. Caspase-8 and FADD are core components of the canonical death-inducing signaling complex (DISC), which is formed when the extrinsic apoptosis pathway is triggered via binding of ligand to death receptors (DRs) of the tumor necrosis factor (TNF) receptor (TNFR) superfamily, e.g., TRAIL-R1/R2 or CD95. Ligand binding induces clustering of DRs, enabling recruitment of the adapter protein FADD via its death domain (DD) to the intracellular DDs of oligomerized DRs. Procaspase-8 (defined as full-length Caspase-8 monomer) then binds to FADD via death effector domain (DED) interactions, thereby forming the DISC. FADD and Caspase-8 are not only key proteins of the canonical CD95/TRAIL-R DISC, but are also core components of the cytosolic TNF complex II^1,2^, the ripoptosome^3,4^ as well as RIPK1/RIPK3-containing necroptotic complexes^5^. In all these scenarios, DED-mediated interaction between FADD and Caspase-8 is critical for cell death induction^6^. Thus, uncovering the molecular architecture of the core FADD:Caspase-8 complex, and how this is affected by interaction with other regulatory partners is essential for understanding how cell death is regulated.

Extensive biochemical characterization of the native CD95/TRAIL DISC isolated from cells revealed that FADD was present at sub-stoichiometric levels compared to both DRs and Caspase-8^7,8^. These studies suggested that following ligand-induced DR oligomerization and DD-mediated FADD recruitment^9–11^, FADD undergoes a conformational change that enables recruitment of multiple Caspase-8 molecules. This led to the proposal of the DED chain model in which Caspase-8 proteins interact sequentially via their tandem DEDs (tDEDs). The proposed helical chain of Caspase-8 molecules, nucleated by a single FADD molecule, is able to form via the interaction of a canonical ‘FL motif’ on one Caspase-8 DED2 into the DED1 pocket region on the adjacent Caspase-8^7^. This protein–protein interaction has subsequently been named a Type I interaction^12^. These DED chains or ‘death effector filaments’ have also been visualized by confocal microscopy by expressing GFP-labeled Caspase-8 tDEDs-only^7,13^. Subsequent Cryo-Electron Microscopy (Cryo-EM) studies of the tDED-only filament revealed that, rather than being a single strand of tDED molecules, filaments are made up of three tDED strands which wind around each other to form a triple helix structure^12^.

Early biochemical studies showed that procaspase-8 exists as a monomer and enzymatic activation requires dimerization of the catalytic domains of two Caspase-8 molecules, each made up of a large (p18) and small (p10) subunit^14–16^. On dimerization, a series of auto-cleavage events occur at key aspartic acid residues, resulting in removal of the inter-domain linker between the large and small subunits^16,17^. Structural determination of the fold of the cleaved catalytic domains showed that the two catalytic domains must associate in an anti-parallel orientation relative to each other^14,15,18^. This interaction is thought to result in modifications to the tertiary structure that facilitate formation of the substrate binding and catalytic sites of the Caspase-8 dimer. Importantly, both dimerization and auto-processing of the catalytic domains of procaspase-8 are required to produce an active protein capable of driving apoptosis^19,20^. To date, structural information on the catalytically inactive Caspase-8 p18-p10 monomer is solely based on NMR analysis of a truncated (ΔDED) recombinant Caspase-8. This revealed that the inter-domain linker between the large and small subunits blocks the active site^21^, rendering the Caspase-8 monomer catalytically inactive. However, there is an important caveat to the interpretation of these studies, namely that all the data are for truncated recombinant Caspase-8 lacking the tDEDs at the N-terminus of the natural protein.

Thus, although there has been a concerted effort to determine the structure of the individual proteins and the overall architecture of the DISC, to date, all published structures have been of individual/truncated domains of isolated recombinant proteins^9–11^. Often mutations have also been introduced to facilitate expression of high concentrations of soluble proteins amenable to structural determination by X-ray crystallography^12,14,15,18,22,23^ or NMR^21^. Since procaspase-8 recruitment to the DISC (and other FADD-based signaling platforms) is mediated via FADD-nucleated tDED interactions, these studies cannot predict the effect of tDEDs on structural dimerization/activation of procaspase-8. Moreover, the impact of recruitment of other tDED proteins including known regulators of FADD:Caspase-8 signaling complexes e.g., c-FLIP isoforms/Caspase-10, on tDED filament architecture/procaspase-8 signaling outcome has not been explored.

Furthermore, it remains unknown how the proposed tDED-only filament structure facilitates Caspase-8 catalytic domain dimerization/activation. In this study, we have solved this conundrum by co-expressing soluble full-length procaspase-8 together with FADD. Using electron microscopy we now visualize the structural architecture of the FADD-nucleated full-length Caspase-8 complex. Crucially, our data explain how the triple helix tDED filament structure facilitates dimerization and activation of procaspase-8. Furthermore, by incorporation of c-FLIP_S_ into the complex, we provide a structural explanation as to how c-FLIP_S_ both terminates tDED filament assembly and inhibits Caspase-8 activation. Thus, by visualizing and determining the structure of the core FADD:Caspase-8 activation complex, we provide insights into cell death regulation, not only by the DISC but also across similar FADD:Caspase-8 signaling platforms that co-ordinate cell fate.

## Results

### Full-length Caspase-8 filament formation is nucleated by FADD

**Fig. 1:**
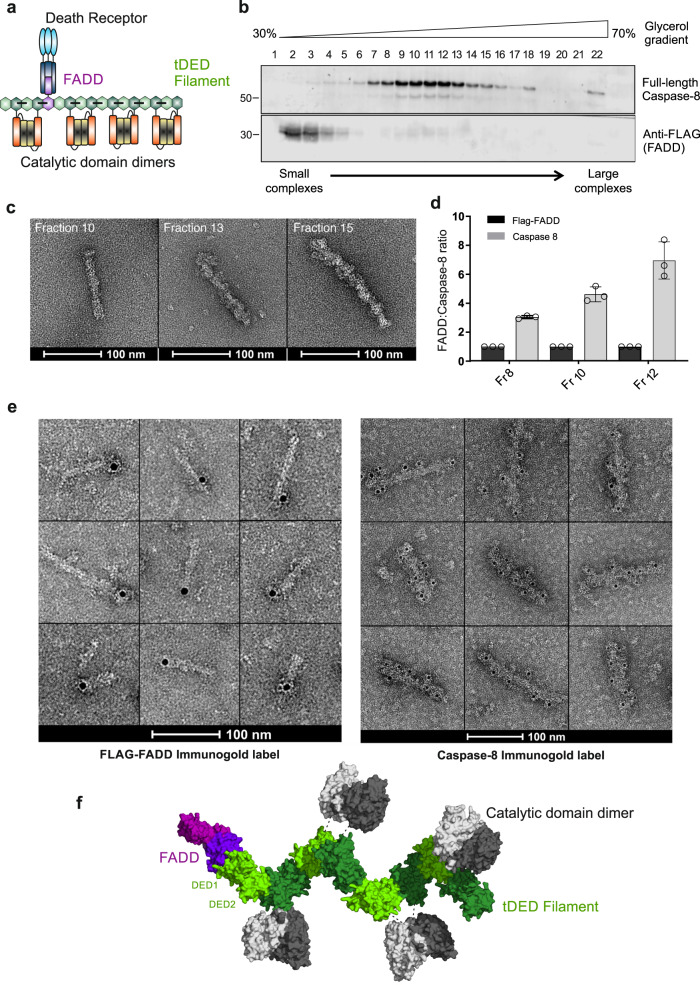
Caspase-8 filament formation is nucleated by FADD. **a** Schematic representation of the architecture of DISC. **b** Western blot analysis of Glycerol Density Gradient purification of soluble Flag-FADD:Caspase-8 complexes. Western blots are representative of 3 independent experiments. **c** Negative-stain EM of FADD:Caspase-8 filaments purified from different gradient fractions (Fractions 10, 13 and 15). Micrographs are representative of 3 independent experiments. **d** Label-free quantitative mass spectrometry determination of FADD:Caspase-8 ratio in Glycerol Density Gradient fractions (Graph shows Mean ± SD, *n* = 3 technical repeats; from a representative experiment of 3 biologically independent experiments). **e** Immuno-gold labeling of FLAG-tag and Caspase-8 p18 subunit in purified filament complexes from Fraction 10 within the peak visualized by EM. Micrographs are representative of 3 independent experiments. **f** Structural model of FADD:Caspase-8 tDED single-chain complex. Source data are provided as a Source Data file.

To investigate the molecular architecture of FADD:Caspase-8 complexes (Fig. 1a), FLAG-tagged FADD and full-length catalytically inactive Caspase-8 (C360A; hereafter referred to as Caspase-8) were co-expressed in HEK 293F cells (Caspase-8-null 293F, clone 1D3; Supplementary Fig. 1) and the resultant soluble complexes isolated using FLAG affinity resin. Complexes were eluted from the beads and purified using glycerol density gradient centrifugation. Analysis of the purified gradient fractions revealed a heterogeneous mixture of complexes spread over 12 fractions (Fig. 1b). Most of the complexes eluted in fractions 7-13 where both FADD and Caspase-8 could be detected by Western blotting. Analysis of complexes in selected fractions across the gradient by negative-stain transmission electron microscopy (EM) showed that the complexes were filament-like in shape and increased in length across the gradient (Fig. 1c and Supplementary Fig. 2). Furthermore, analysis of the stoichiometry of FADD and Caspase-8 in the different gradient fractions by quantitative label-free LC-MS/MS revealed increasing amounts of Caspase-8, relative to FADD as the complexes increased in length (Fig. 1d).

We next investigated the composition of these FLAG-FADD:Caspase-8 complexes using Immuno-gold labeling of the component proteins (Fig. 1e and Supplementary Fig. 3). Complexes were labeled with either anti-FLAG to determine the position of FADD or with anti-Caspase-8. Anti-FLAG labeling revealed a single gold moiety positioned at one end of the complex, whereas anti-Caspase-8 labeling resulted in multiple gold moieties detected along the filament. These results demonstrate that full-length Caspase-8 forms complexes that are nucleated by FADD, which extend their filament length by the addition of an increasing number of Caspase-8 molecules (Fig. 1f).

### Negative-stain EM structure determination of FADD:Caspase-8 filament

**Fig. 2:**
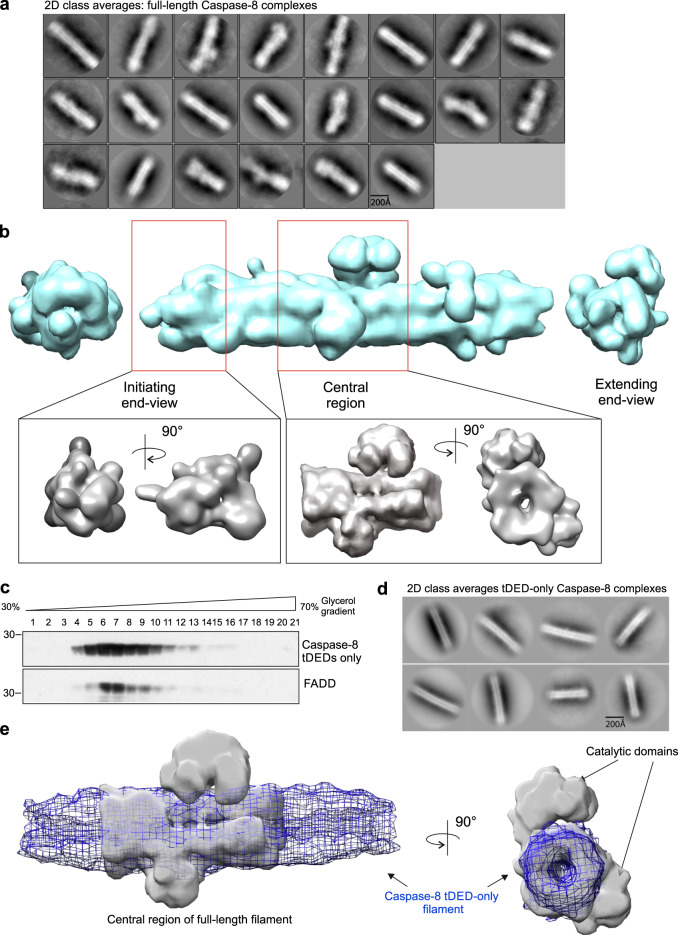
Negative-stain EM model of FADD:full-length Caspase-8 filament. **a** Reference-free 2D class averages generated in RELION of negative-stain EM of FLAG-FADD:Caspase-8 complexes. **b** Initial model generated in CryoSPARC. Whole filament shown in blue (27 Å) with views of ‘closed’ initiating end (left) and ‘open’ extending end (right). Gray EM volumes are refinements of masked sections of the full filament; both the initiating end (17 Å) and central Caspase-8 only region of complex (14.5 Å). **c** Western blot analysis of Glycerol Density Gradient purification of soluble FADD:Caspase-8 tDED-only (expressed together with FLAG-c-FLIP_S_ to stabilize the tDED-only complexes). Western blots are representative of 3 independent experiments. **d** Reference-free 2D class averages generated in CryoSPARC of negative-stain EM of FADD:Caspase-8 tDED-only complexes. **e** Overlay of initial model generated in CryoSPARC of central region of Caspase-8 tDED-only complex (mesh) with EM volume of central region of full-length Caspase-8 filament. Source data are provided as a Source Data file.

Negative-stain EM data of FLAG-FADD:Caspase-8 complexes, collected from Fraction 10 within the peak of the density gradient (Fig. 1b), provided sufficiently homogeneous complexes for structural determination. Filaments of similar lengths were picked using a mask of maximal length 700 Å and reference-free 2D class averages generated in RELION using the single-particle approach (Fig. 2a and Supplementary Figs. 4–6). While some 2D classes were different lengths, there was clearly a lower level of density in the center of each filament suggesting the core complex could be a tubular structure. Furthermore, many of the filaments were decorated with extra density which we hypothesized may be the presence of the catalytic domains of full-length Caspase-8. An initial low-resolution 3D model of the data was generated in CryoSPARC (Fig. 2b). This map (Supplementary Fig. 7) had stronger density near the center of the reconstruction and substantially weakened density at the periphery. This density gradient is most likely due to a combination of: (i) compositional heterogeneity near the ends of the filaments; (ii) conformational flexibility of the individual particles that are used in the reconstruction; (iii) uncertainty in the angular alignment of the particles. These elements will show stronger effects at the edge than at the center of the reconstruction. We further refined the 3D model with various masked sub-volumes of the EM map and then refined these separately. This procedure revealed that the two ends of the complex were different (Supplementary Fig. 8), with one end exhibiting an open helical-shaped configuration. We have named this the ‘extending’ end (Supplementary Fig. 9), and hypothesize this is where additional Caspase-8 molecules would bind to extend the filament. In contrast, the other end of the complex displayed a closed tapered appearance (Fig. 2b), which we propose is the ‘initiating’ end of the filament where FADD is positioned. 3D refinement of the central region (Fig. 2b and Supplementary Fig. 7) revealed that the central filament structure was indeed a hollow tubular structure. However, additional discreet EM volumes were attached periodically to the exterior, suggesting that these may map to the catalytic domains of Caspase-8.

To determine if the discreet EM volume decorating the exterior of the filament structure was indeed the p18-p10 catalytic domains of Caspase-8, the tDEDs of Caspase-8 without catalytic domains were expressed. The FADD-nucleated Caspase-8 tDED complexes were again purified using glycerol density gradient centrifugation (Fig. 2c) and negative-stain EM data collected. Complexes, of the same length as full-length Caspase-8 complexes, were analyzed in CryoSPARC. The resultant reference-free 2D class averages (Fig. 2d) were filamentous in shape and exhibited a lower central EM density, similar to that previously observed in full-length Caspase-8 complexes (Fig. 2a). A low-resolution 3D model generated in CryoSPARC (Fig. 2e; mesh) revealed a smooth tube-shaped EM volume. Importantly, overlaying this tDED-only filament volume onto the central region of the full-length Caspase-8 3D map, confirmed that the core central filament structure is composed of Caspase-8 tDEDs while the additional EM volume decorating the full-length Caspase-8 model most likely represents the catalytic domains (Fig. 2e).

**Fig. 3:**
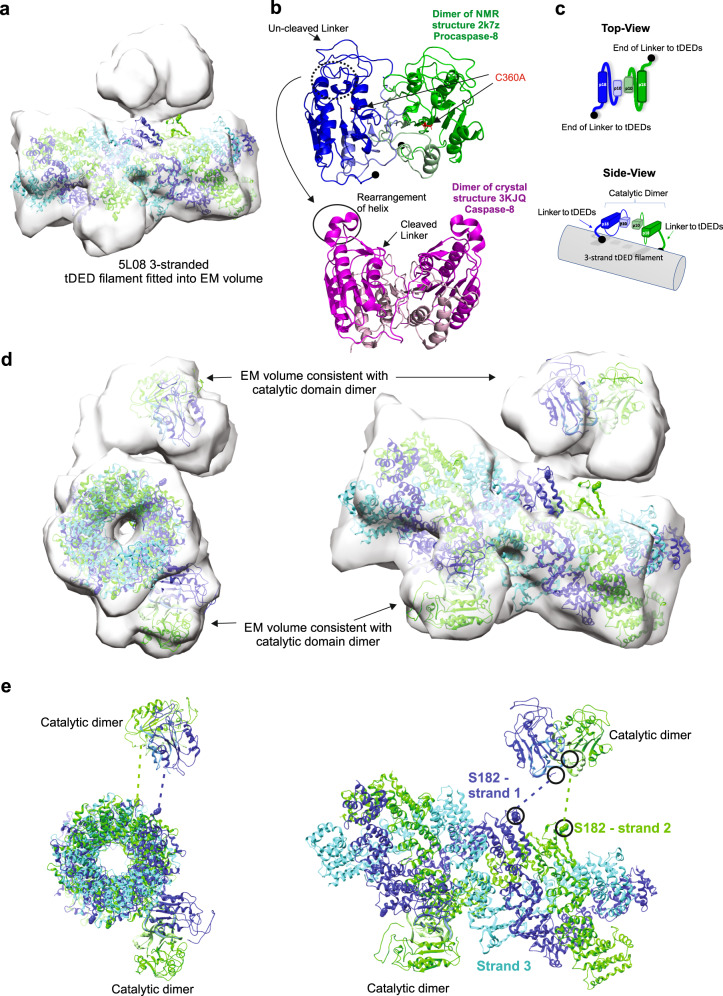
Core tDED triple-helix facilitates inter-strand Caspase-8 catalytic domain anti-parallel dimerization. Refined central region of full-length Caspase-8 filament with, **a** published tDED-only structure (5L08;^12^) fitted as a rigid body. **b** Ribbon NMR-based structural model of dimerized uncleaved catalytic subunit domains (2k7z; ^21^; upper panel, C360A in red and K224 as filled circle) and ribbon X-ray structure of cleaved catalytic subunit dimer (3KJQ;^14^; lower panel). **c** Schematic representation of uncleaved Caspase-8 p18 (large) and p10 (small) catalytic domains in anti-parallel orientation relative to tDED domains. **d** Fitted ribbon diagram of Caspase-8 tDEDs and catalytic domain assembly. Each strand of the helix is coloured; blue and green strands contain aligned DEDs, while in the cyan strand the DEDs are offset. **e** Caspase-8 catalytic domain subunit colours reflect the DED strand from which they originate. Distance between the termini connected by the dashed lines is approximately 35 Å, which can be accommodated by the 32 amino acid flexible linker between the tDED and catalytic domains of Caspase-8.

To determine the core structure of the FADD-nucleated full-length Caspase-8 activation complex, the published structure of the tDEDs alone (5L08,^12^) was fitted as a rigid body into the EM volume of the FADD:full-length Caspase-8 (Fig. 3a). The published tDED-only structure is made up of three tDED strands in a triple helix, where each strand is consistent with the tDED-based helical model proposed by Dickens et al.^7^ (Supplementary Fig. 10). tDEDs in Strands 1 (blue) and 2 (green) are rotated ~20˚ along the axis of the filament relative to each other, resulting in the DED2 domains being “aligned” with their carboxy termini within 35 Å of each other. The DED2 domains on Strand 3 (cyan) however, are rotated ~70˚ relative to Strand 1 so the carboxy termini are further apart (~50 Å); we, therefore, term this the “offset” strand (Supplementary Fig. 11). Importantly, this structure fits well within the tubular-shaped core EM volume (Fig. 3a); furthermore, we observed that all complexes formed were of a uniform width suggesting that all three tDED strands extend simultaneously.

We then investigated if/how this tDED triple helix filament structure could facilitate Caspase-8 catalytic domain activation. NMR-based structural determination of monomeric Caspase-8 catalytic domains revealed that the inter-domain linker between the large (p18) and small (p10) catalytic subunits blocks the active site, rendering the Caspase-8 monomer inactive^21^ (Fig. 3b). Upon dimerization, a series of auto-cleavage events occur at key aspartic acid residues, resulting in removal of the inter-domain linker^14^ (Fig. 3b). The two catalytic domains within the dimer then associate in an anti-parallel orientation relative to each other^16^ (Fig. 3c). This anti-parallel orientation is crucial for formation of the substrate binding/catalytic sites of the Caspase-8 dimer. In our study full-length catalytically inactive (C360A) was expressed, therefore we modeled an uncleaved Caspase-8 catalytic domain dimer (Fig. 3b, based on 2k7z;^21^) to fit as a rigid body into the model of the FADD:full-length Caspase-8 EM volume (Fig. 3d). Importantly, this structure fits well within the EM volume we had hypothesized to be the catalytic domains within the Caspase-8 EM volume (Fig. 2e).

Investigation of the relative spatial orientations of the known structures fitted into the EM volume (Fig. 3e), revealed how the tDED filament of Caspase-8 facilitates anti-parallel dimerization of the catalytic domains. Thus, the dimerized catalytic subunits originate from Caspase-8 tDEDs that reside on different, but aligned strands (labeled Strands 1 (blue) and 2 (green)) within the tDED triple helix (Fig. 3e). At this resolution, there is no identifiable EM volume for the flexible linker that connects the tDED and p18 catalytic subunit of full-length Caspase-8. However, in our model the positioning of the second Caspase-8 catalytic dimer is such that its corresponding tDEDs also reside on the aligned Strands 1 and 2 within the triple helix (Fig. 3e).

### tDED helical filament architecture is essential for activation of Caspase-8

**Fig. 4:**
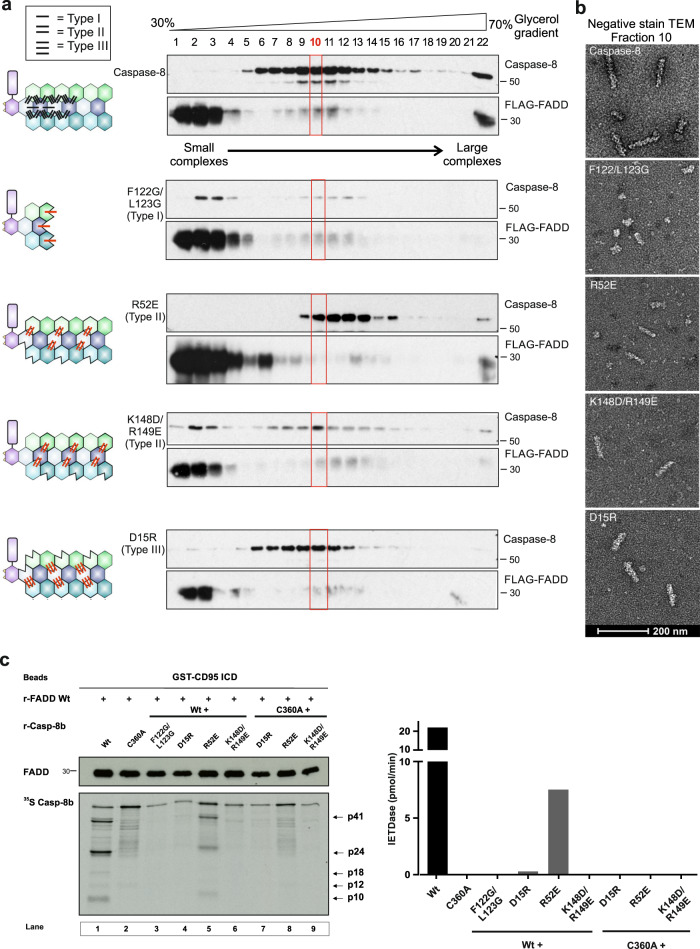
tDED filament architecture is essential for Caspase-8 catalytic activity. **a** Left Panel, Schematics illustrate the position of Caspase-8 tDED Type I, II and III interaction mutants within triple helix filament. Right Panel, Western blot analysis of Glycerol Density Gradient profiles of full-length Caspase-8 C360A (Caspase-8) and full-length Caspase-8 tDED interaction mutants. Western blots are representative of 3 independent experiments. **b** Negative-stain EM of FADD in complex with full-length Caspase-8 C360A (Caspase-8) or Caspase-8 tDED interaction mutants. Representative filaments visualized are from equivalent gradient fractions (Fraction 10), for each complex. Micrographs are representative of 3 independent experiments. **c** Reconstituted CD95 DISC (r-DISC; GST-CD95-IcD:r-FADD:r-Casp-8), assembled with Wt (Wild-type) or mutant ^35^S r-Casp-8b. Beads were analyzed for r-Casp-8b cleavage and IETDase activity. Data shown are from one experiment, representative of 3 independent experiments. Source data are provided as a Source Data file.

As the tDED helical filament consists of 3 strands^12^, and having established that Caspase-8 catalytic subunit dimers originate from Caspase-8 molecules residing on adjacent strands (Fig. 3e, Strands 1 and 2), we wished to determine the significance of both intra- and inter-strand interactions within the FADD:Caspase-8 complex architecture and their effect on Caspase-8 activation. Type I contacts occur within a single strand (intra-strand) between the canonical FL motif on DED2 and adjacent pocket residues on DED1 of the next Caspase-8 molecule, and are essential for Caspase-8 activity^7,24^ (Fig. 4a, schematic). By contrast, the interactions identified by Cryo-EM in the Caspase-8 tDED-only structure describe tDED inter-strand contacts termed Type II and III interactions^12^ (Fig. 4a, schematic). Mutation of the intra-strand Caspase-8 Type I contact F122G/L123G resulted in a significant shift in the buoyant density (size) of the FLAG-FADD:Caspase-8 complexes purified by density gradient centrifugation (Fig. 4a). There was a marked decrease in the amount of protein filament complexes, as the majority of expressed proteins eluted in fractions where uncomplexed FLAG-FADD normally sediments. Importantly, TEM analysis and quantitation of the Type I mutant complexes in Fraction 10, showed fewer complexes compared to FLAG-FADD:Caspase-8 and they appeared to be globular aggregates rather than filaments (Fig. 4b and Supplementary Fig. 12). Mutation of residues R52 (Type II), K148/R149 (Type II) and D15 (Type III), all of which were identified as being involved in inter-strand contacts, produced contrasting results. The R52E mutant sedimented as a tighter peak (over 7 fractions) and contained a mixture of slightly thinner filaments and fragmented globular structures (Fig. 4a,b and Supplementary Fig. 12). Mutation of K148D/R149E resulted in a significant proportion of uncomplexed protein sedimenting essentially as monomeric proteins and a reduced number of complexes, which were shorter in length than FLAG-FADD:Caspase-8 filaments (Fig. 4b, compare Caspase-8 with contact mutants). The D15R mutation produced fewer complexes, which sedimented similarly to FADD:Caspase-8 complexes and on TEM appeared as shortened filaments (Fig. 4b and Supplementary Fig. 12). Thus, the most notable mutation which affected the FADD:Caspase-8 structure as visualized/quantified by TEM was mutation of the canonical Type I contact, which completely abrogated filament formation. However, mutation of the inter-strand contacts (Type II and III) resulted in abnormal filament structures of variable size and shape.

A key question is how does abnormal filament shape and size impact on Caspase-8 catalytic domain dimerization and subsequent enzymic activation. We tested this using a functional reconstituted CD95 DISC (r-DISC) model which, as described previously^19^, uses GST-tagged CD95 intracellular domain (CD95-IcD) complexed with recombinant FADD (r-FADD) to form a nucleating scaffold that recruits/activates recombinant procaspase-8. In this r-DISC model, wild-type procaspase-8 is recruited to the r-DISC, auto-catalytically cleaved to its signature cleavage products and Caspase-8 proteolytic activity determined by cleavage of the fluorogenic substrate Ac-IETD.AFC (IETDase activity; Fig. 4c). Mutation of the catalytic site (C360A) did not block procaspase-8 recruitment but autocatalytic inter-domain cleavage and IETDase activity were inhibited (Fig. 4c). Additionally, F122G/L123G (Type I), D15R (Type III) and K148D/R149E (Type II in DED2) mutations markedly reduced procaspase-8 incorporation to the r-DISC and completely blocked generation of cleavage fragments and IETDase activity (Fig. 4c, Lanes 3, 4 & 6). The R52E mutant (Type II in DED1) was recruited to the r-DISC, but was only marginally active as judged by generation of cleavage fragments and IETDase activity which was less than a third of the activity observed with wild-type Caspase-8 (Fig. 4c, Lane 5). Thus, mutation of the canonical Type I interaction mediated via the FL motif not only blocks filament formation but also totally prevents Caspase-8 activation. Disruption of contacts that play a role in inter-strand interactions (Type II and III) also clearly influence tDED helical filament architecture. We earlier determined that catalytic domains from the aligned Strands 1 and 2 within the triple helix dimerize, thus disruption of the helical architecture markedly abrogates formation of the Caspase-8 catalytic dimer active site and enzymic activation. To extend this, we assessed the ability of Caspase-8 Type 1, II and III contact mutants to rescue CD95-induced cell death in Caspase-8-deficient Jurkat cells (Supplementary Fig. 13). Importantly, in Jurkat T cells which are termed ‘Type 2 cells’ as they additionally rely on mitochondrial amplification to successfully execute apoptotic cell death^25^, only wild-type Caspase-8 was able to rescue CD95-induced apoptosis. Thus, although the Caspase-8 R52 mutant had retained albeit low levels of caspase activity in a pure reconstituted CD95 DISC (Fig. 4c), this low level DISC activity alone is not sufficient to drive CD95-induced cell death in ‘Type 2 cells’.

### c-FLIP_S_ alters the architecture of the FADD:Caspase-8 complex thereby preventing Caspase-8 filament elongation

**Fig. 5:**
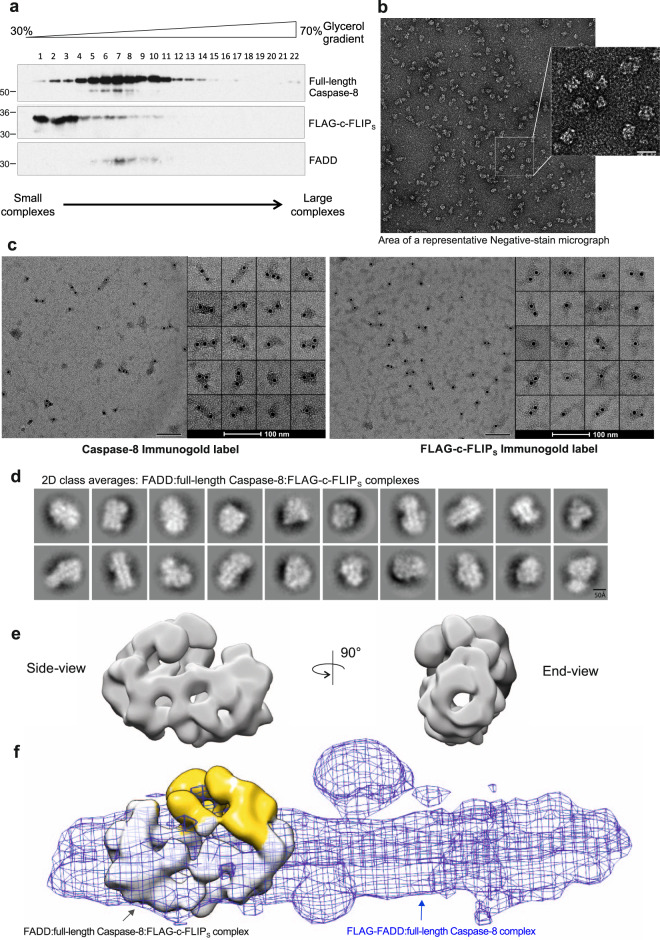
c-FLIP short (c-FLIP_S_) prevents Caspase-8 filament elongation altering the architecture of the complex. **a** Western blot of Glycerol Density Gradient profiles of FADD:full-length Caspase-8:FLAG-c-FLIP_S_ complexes. Western blots are representative of 3 independent experiments. **b** Representative negative stain micrograph of FADD:full-length Caspase-8:FLAG-c-FLIP_S_ complexes (Scale bar, 100 nm; inset, 25 nm). Micrographs are representative of 3 independent experiments. **c** Immuno-gold labeling of p18 subunit of Caspase-8 and FLAG-tag on purified FADD:full-length Caspase-8:FLAG-c-FLIP_S_ complexes (Fraction 6), visualized by EM. Micrographs are representative of 3 independent experiments. **d** Reference-free 2D class averages generated in CryoSPARC of negative-stain EM of FADD:full-length Caspase-8:FLAG-c-FLIP_S_ complexes. **e** Different orientations of low-resolution EM volume of FADD:full-length Caspase-8:FLAG-c-FLIP_S_ complexes; side-view (left) and end-view (right). **f** Overlay comparison of FADD:full-length Caspase-8:FLAG-c-FLIP_S_ complex negative-stain EM volume and FADD:full-length Caspase-8 complex EM volume; additional EM volume only present in c-FLIP_S_-inhibited complex is shown in yellow. Source data are provided as a Source Data file.

Various tDED-containing proteins are known to bind to FADD:Caspase-8 complexes and thereby modulate Caspase-8 activity (reviewed in^6^). One of the best characterized of these is c-FLIP which predominantly exists as two isoforms, c-FLIP_L_ and c-FLIP_S_. c-FLIP_S_ functions as a key inhibitor of Caspase-8 activation in several FADD:Caspase-8 signaling platforms, including the DISC, ripoptosome and necrosome^3,4,24^. The mechanism whereby c-FLIP_S_ disrupts Caspase-8 activation is unknown; we therefore wished to determine the effect of c-FLIP_S_ on the architecture of the FADD:Caspase-8 complex to uncover how c-FLIP_S_ inhibits Caspase-8 activity. To ensure the complexes examined all contained c-FLIP_S_, we transiently co-expressed FLAG-tagged c-FLIP_S_ with untagged full-length FADD and full-length Caspase-8 using a FADD:Caspase-8:FLAG-c-FLIP_S_ ratio of 1:3:6. This generated c-FLIP_S_-containing FADD:Caspase-8 complexes which were affinity purified using FLAG affinity resin, eluted and further purified using glycerol density gradient centrifugation. Analysis of the gradient fractions revealed that the addition of c-FLIP_S_ resulted in complexes which equilibrated in lower density fractions towards the top of the gradient, indicating they were smaller (Fig. 5a) than the FADD:Caspase-8 filaments (Fig. 1b). Consistent with this, analysis of the complexes by negative-stain EM revealed that the FADD:Caspase-8:FLAG-c-FLIP_S_ complexes were shorter and more globular (Fig. 5b) than the FADD:Caspase-8 complexes (Fig. 1c). Immuno-gold labeling of Caspase-8 in the FADD:Caspase-8:FLAG-c-FLIP_S_ complexes suggests that short filaments are formed with multiple Caspase-8 proteins (Fig. 5c). Unlike Caspase-8, c-FLIP_S_ was positioned at one or both ends of the complex (Fig. 5c). Importantly, this suggests that once c-FLIP_S_ is recruited into the complex further Caspase-8 molecules do not bind.

Reference-free 2D class averages of the negative-stain complexes (Fig. 5d and Supplementary Fig. 14) revealed that a Caspase-8 filament begins to extend with similar characteristics to that observed previously when Caspase-8 was co-expressed with FADD alone (compare Fig. 5d with Fig. 2a). In addition, some extra density is clearly visible along the filament reminiscent of what we have shown previously in the FLAG-FADD:Caspase-8 complex to be the catalytic domains of Caspase-8. Interestingly, with these smaller complexes, there are clear end-views of the complex visible down the length of the filament. These views were used to generate an initial low-resolution 3D model of the FADD:Caspase-8:FLAG-c-FLIP_S_ complex (Fig. 5e and Supplementary Fig. 15). Comparison of the negative-stain EM volumes of FADD:Caspase-8:FLAG-c-FLIP_S_ and FLAG-FADD:Caspase-8 (Fig. 2b), clearly shows that the presence of c-FLIP_S_ in the complex prevents tDED helical filament extension resulting in a shortened complex (Fig. 5f). Moreover, in the presence of c-FLIP_S_, the additional EM volume normally occupied by the catalytic domains of Caspase-8 is reduced and its overall architecture appears to be different (Fig. 5f, yellow region).

### c-FLIPs blocks Caspase-8 filament elongation by steric hindrance of the canonical tandem DED binding site

**Fig. 6:**
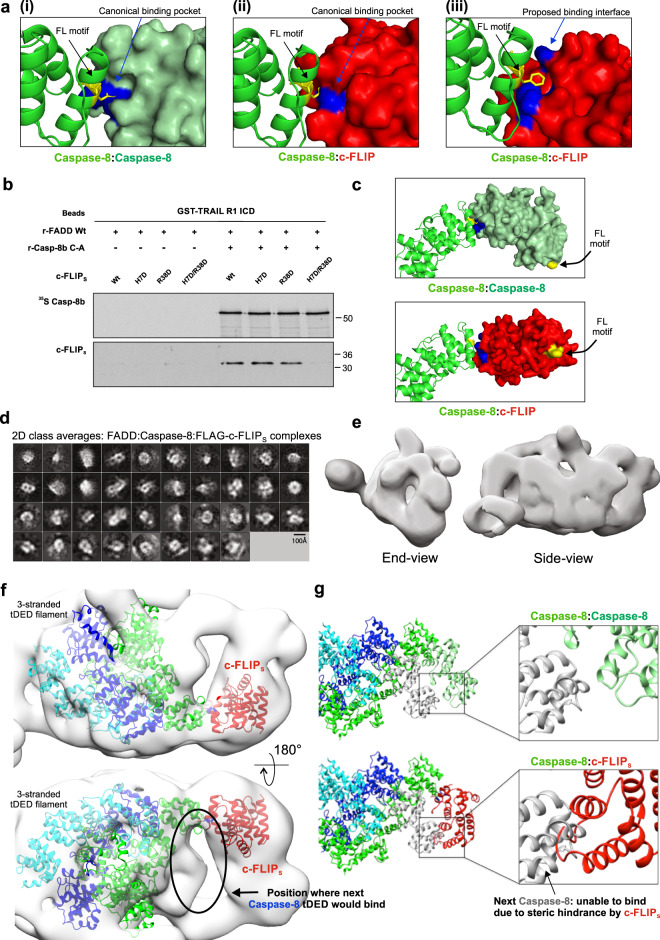
c-FLIP_S_ blocks Caspase-8 filament elongation by steric hindrance of canonical DED1 binding site. **a** HADDOCK modeling indicates c-FLIP DED1 pocket cannot bind Caspase-8 DED2 FL motif in the same way as Caspase-8:Caspase-8 (i), due to steric hindrance (ii), rather it binds via a predicted non-canonical interaction motif, including other key residues (iii). **b** Reconstituted DISC (r-DISC: GST-TRAIL-R1 ICD:FADD:Caspase-8:c-FLIP_S_), assembled with WT or mutant c-FLIP_S_. Data shown are from one experiment, representative of 3 independent experiments. **c** Structural model of the effect of the canonical *versus* predicted non-canonical interaction on position of c-FLIP DED2 FL motif. **d** Reference-free 2D class averages generated in RELION3 of Cryo-EM analysis of FADD:full-length Caspase-8:FLAG-c-FLIP_S_ complexes and **e** 3D model refined to 13 Å in RELION3. **f** Refined model with published tDED-only structure (5L08;^12^) fitted as a rigid body and the threaded structure of c-FLIP_S_ orientated relative to the Caspase-8 filament via the predicted non-canonical binding motif; position where next Caspase-8 would be recruited on the adjacent tDED strand is circled. **g** Structural model of how c-FLIP_S_ binding (to green strand) blocks further recruitment of Caspase-8 molecules to the adjacent, aligned strand (blue) within the helix. Source data are provided as a Source Data file.

We have previously shown that c-FLIP_S_ recruitment to FADD:Caspase-8 complexes is primarily mediated via co-operative binding of c-FLIP_L/S_ to Caspase-8^24^. In the case of c-FLIP_S_, this results in complete inhibition of Caspase-8 activity. The negative-stain EM data established that the presence of c-FLIP_S_ in the FADD:Caspase-8 complex prevents tDED filament extension resulting in a shortened complex (Fig. 5f). We next sought to obtain structural insight into how c-FLIP_S_ interacts with Caspase-8. Through analysis of the predicted fold of c-FLIP_S_ from the protein sequence, we identified key differences between the tDED domains of c-FLIP_S_ and Caspase-8 (Fig. 6a). Caspase-8:Caspase-8 filament elongation relies on the canonical ‘Type I’ tDED interaction between DED2 F122/L123 motif and the Y8 pocket on DED1 (Fig. 6a, part i). However, when this interaction is modeled for Caspase-8:c-FLIP_S_, it results in steric clashes between Caspase-8 DED2 and c-FLIP_S_ DED1 (Fig. 6a, part ii), suggesting an alternative ‘non-canonical’ interface is involved. Using HADDOCK (High Ambiguity Driven protein-protein DOCKing; Webserver: haddock.science.uci.nl, Supplementary Fig. 16), we identified a potential non-canonical binding orientation for Caspase-8:c-FLIP_S_ which, in addition to the canonical H7 pocket residue, involves residue R38 of DED1 in c-FLIP_S_ (Fig. 6a, part iii). Using the r-DISC model, we tested the effect of mutating these residues (Fig. 6b). When compared to WT c-FLIP_S_, the individual c-FLIP_S_ mutants, H7D and R38D, retained their ability to bind to Caspase-8. However, with the double mutant H7D/R38D, c-FLIP_S_ binding to Caspase-8 was abolished, confirming that both residues are involved in the DED binding interface between Caspase-8 and c-FLIP_S_. Consequently, binding of c-FLIP_S_ to Caspase-8 via this non-canonical interaction motif alters the orientation of the FL motif on DED2 of c-FLIP_S_. As a result, c-FLIP_S_ DED2 FL motif would not adopt the canonical orientation of Caspase-8 DED2 FL motif in a Caspase-8:Caspase-8 filament (Fig. 6c). This suggests that c-FLIP_S_ likely alters the overall topology of c-FLIP_S_-containing Caspase-8 complexes.

To further resolve the c-FLIP_S_ interaction with Caspase-8 within the FADD:Caspase-8 complex, we collected a Cryo-EM data set on the FADD:Caspase-8:FLAG-c-FLIP_S_ complex (Supplementary Figs. 17–20). Using a mask of 240 Å, complexes were picked using CrYOLO^26^ to generate templates for automatic picking in RELION3^27^. 2D reference-free class averages were then generated in RELION3 (Fig. 6d) and Cryo-EM 3D reconstruction resulted in a 13 Å EM map (Fig. 6e). We fitted the known protein structures into this map, choosing the hand of the envelope that gave the best fit. Further validation of our 3D reconstruction by tilt-pair analysis, however, was not possible due to the low signal-to-noise ratio in our raw data. Using tDED interactions from the Cryo-EM structure of tDEDs alone (5L08;^12^) and our threaded structure of c-FLIP_S_ orientated relative to Caspase-8 via our predicted non-canonical binding interface (H7/R38), the EM volume accommodates a short Caspase-8 triple helix filament with c-FLIP_S_ bound in the predicted binding site (Fig. 6f). This further strengthens our finding that c-FLIP_S_ binding to Caspase-8 occurs via a non-canonical interface. Moreover, the refined EM map shows that when c-FLIP_S_ is bound to the next available tDED (green strand) within the Caspase-8 filament, the next Caspase-8 molecule is not recruited to the adjacent aligned strand (blue) as there is no volume present in the reconstructed EM map of the complex (Fig. 6f, lower panel). It appears that Caspase-8 recruitment is inhibited at this site due to steric hindrance, as c-FLIP_S_ DED2 occupies the space where the next Caspase-8 molecule would bind on the adjacent strand of the filament (Fig. 6g). Thus, binding of c-FLIP_S_ abrogates chain elongation of the neighboring strand, preventing long filament formation.

### Binding of c-FLIP_S_ prevents Caspase-8 catalytic domain dimerization

**Fig. 7:**
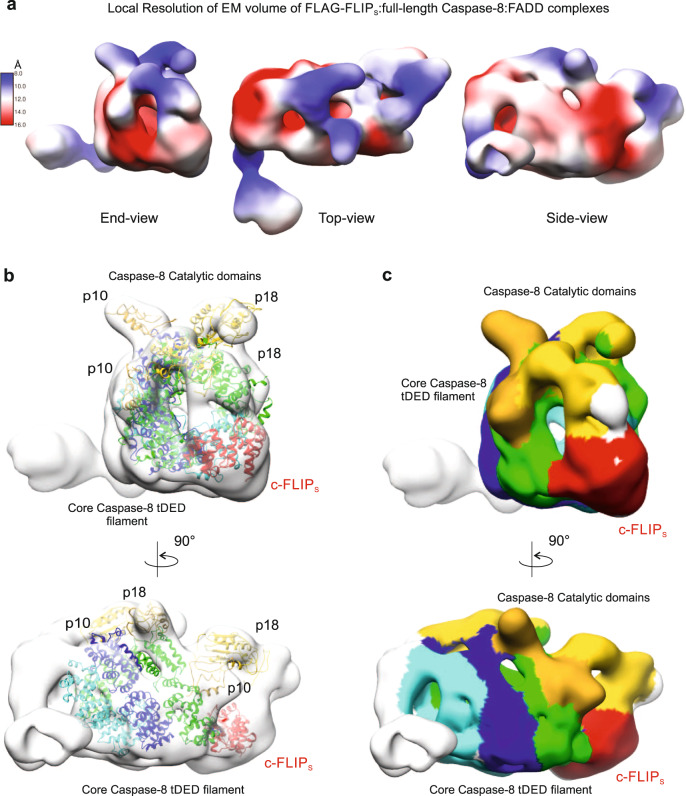
Binding of c-FLIP_S_ prevents Caspase-8 catalytic domain dimerization. **a** Analysis of local resolution of 3D refined Cryo-EM map of FADD:full-length Caspase-8:FLAG-c-FLIP_S_ complex. **b** Refined map with published tDED-only structure (5L08;^12^) and the p18 and p10 subunits of the intact catalytic domain dimer (modeled on NMR-based structure, 2k7z;^21^), fitted as a rigid body relative to the Caspase-8 filament. **c** EM volume of Caspase-8 DEDs, c-FLIP_S_ (red) and catalytic domain assembly. Each strand of the DED helix is coloured; tDEDs in blue and dark green strands are aligned, whereas tDEDs in the cyan strand are offset. Catalytic domains are coloured gold or yellow and from their positioning within the EM map have not formed a canonical catalytic dimer.

The Caspase-8 tDED filament (5L08;^12^) and our threaded c-FLIP_S_ structures populate the lower areas of the refined 3D EM map (Fig. 6f), however there is additional volume at the top of the map that could accommodate the catalytic domains of Caspase-8. These form narrow repeating volumes along the top of the map with local resolution estimates (calculated in RELION), indicating they are some of the best aligned features in the map (Fig. 7a). Due to the shape of these empty areas of EM volume it was clear that the catalytic domains were not in their active dimeric conformation as observed previously in the FLAG-FADD:Caspase-8 complex (Fig. 3). Rather, the catalytic domains appear to be monomeric, with the p18/p10 subunits positioned too far apart to allow dimerization (Fig. 7b,c). The positioning of the catalytic domains in the EM map in this ‘open conformation’ rather than in the dimeric antiparallel ‘active state’ now provides a mechanism to explain how c-FLIP_S_ inhibits the catalytic activity of Caspase-8.

## Discussion

Caspase-8 and FADD are key components of several multi-protein complexes that co-ordinate cell fate, including the canonical apoptosis-inducing DISC, the ripoptosome and necrosome^6,28^. We now describe the molecular architecture of the tDED-based filament complex that is formed between full-length procaspase-8 including its catalytic domains and FADD. Characterization of FADD:Caspase-8 complexes, by density gradient centrifugation and negative-stain EM, highlights several key observations. Firstly, the complexes are filament-like in shape similar to the death effector filaments that have been observed when GFP-tDEDs are expressed and visualized by confocal microscopy^7,13^. In addition, the length of these filament structures is heterogeneous. Consistent with this, density gradient centrifugation separated the complexes in order of their Caspase:FADD ratio (i.e., low to high; Fig. 1b). Furthermore, immuno-gold staining confirmed that the complexes are nucleated by FADD and extend by sequential addition of multiple Caspase-8 molecules. This results in filaments of different lengths and in cells it is therefore likely that, upon induction of extrinsic cell death, the exact length of the complex formed will vary depending on the local concentration of components available^7,8^, with cleavage of Caspase-8 serving as a negative feedback signal that triggers disassembly of the complex.

The complexes visualized by negative-stain EM were analyzed using a single particle approach to establish a 3D EM map. The initial reference-free 2D class averages revealed several notable features. Firstly, there was less EM density in the center of each complex, and secondly, extra EM density was observed periodically along the length of the central filament structure. We hypothesized and confirmed this repeating extra density to be the catalytic domains of Caspase-8 as it was absent in complexes containing the truncated Caspase-8 tDED-only construct. Finally, the two ends of the complex were structurally different; one (initiating) end was closed while the other (extending) end was open. Possibly due to heterogeneity in the length of the complexes the highest resolution was in the center of the complex. Refinement of the central region of the complex revealed that the central tDED core of the filament was a triple helix, as recently described when the tDEDs were expressed alone, with the published structure (5L08;^12^) fitting into the EM volume as a rigid body. The open end of the complex had the same shape as the end-view of the refinement of the central region, suggesting this end of the complex was open and extending. However, following FADD nucleation, the closed end of the complex appears to be tapered indicating that the helix does not form immediately. We, therefore, propose that, as implied by previous biochemical analysis of the DISC^7,8,24^, a single Caspase-8 molecule initially binds to FADD via a canonical FL motif (Type I) interaction - then subsequent Caspase-8 molecules bind via both intra- and inter-strand (Type I, Type II and Type III) interactions facilitating formation of the triple helix filament. Observation of thousands of complexes by negative-stain EM, and exemplified by the representative images in Fig. 1c, revealed that although the length of the complexes varied (Supplementary Fig. 2), they were all of uniform width. This suggests that all three strands of the triple helix extend simultaneously.

In terms of structural information on the intact catalytically inactive Caspase-8 p18-p10 monomer; our knowledge is solely based on analysis by NMR of a truncated (ΔDED) recombinant Caspase-8; this revealed that the inter-domain linker between the large (p18) and small (p10) subunits blocks the active site. The intact catalytic subunit dimer has a distinct shape (Fig. 3b; 2k7z,^21^), which when modeled as a rigid-body fits into the additional EM volume we have determined to be full-length Caspase-8 catalytic domains in the refined 3D map of the central region of the complex. There is, however, no EM volume for the flexible linker between the tDED domains and the p18 catalytic subunit of Caspase-8, which is likely due to a high level of movement in these regions of the protein. Interestingly, modeling of these known structures into the observed EM volume revealed the relative spatial orientation of the catalytic domains relative to the central tDED helix filament. Based on positioning of the end of Caspase-8 DED2 domain (S182) and initiation of the catalytic domain (D216), it appears that the two catalytic domains making up the active dimer belong to full-length Caspase-8 proteins on adjacent strands within the triple helix, rather than between Caspase-8 proteins positioned on the same strand within the helix. Characterization of the tDED-only triple helix by Cryo-EM^12^ established that within the helix, two of the strands interact so that the DED2 domains are “aligned” to one another, whereas the third strand interacts such that the DED2 domains are “offset”. In this context, we have found that the Caspase-8 catalytic domains originate from DED2 domains on the aligned Strands 1 and 2 within the triple helix. Furthermore, the position of both catalytic domain dimers reveals that Caspase-8 catalytic domain interactions occur repeatedly between the same two aligned strands (Strands 1 and 2), with each dimer being located on different faces of the helix (Fig. 3; Supplementary Movie 1).

To further define the role of the helical arrangement of the DED domains of full-length Caspase-8 in FADD:Caspase-8 complex assembly and subsequent Caspase-8 activation, we focused on key interactions, both within each strand (intra-strand; Type I) and between strands (inter-strand; Type II and III). Mutation of all these key interactions affected the distribution of FADD:Caspase-8 complexes purified by density gradient centrifugation and architecture of the resultant complex when visualized by negative-stain EM (Fig. 4). The canonical F122G/L123G (Type I) mutation predictably produced the most profound effect. However, mutation of the inter-strand contacts confirmed the importance of the overall filament structure and its role in Caspase-8 catalytic activation and induction of cell death. Due to the nature of the arrangement of the three tDED strands within the helix, each inter-strand mutant makes 2 different sets of contacts. Our data suggest that, in order to maintain Caspase-8 activity, it is the correct positioning of DED2 domains on Strand 1 and 2 that is essential to enable the p18-p10 catalytic domains to be correctly positioned to facilitate antiparallel dimerization and activation of Caspase-8^29^. Mutation of K148/R149 (Type II) directly disrupts the contact between the DED2 domains of the aligned Strands 1 and 2. However, it also disrupts the contact between DED2 on Strand 1 and DED1 on the offset Strand 3; a key interaction that correctly orientates the DED2 domain on the aligned Strand 2. Interestingly, mutating D15 on the offset Strand 3 disrupts this same stabilizing interaction between Strands 1 and 3. This further demonstrates the important role the offset Strand 3 plays within the triple helix to stabilize and maintain the correct position of the aligned Strands 1 and 2 for catalytic dimer activation. We, therefore, have uncovered two key aspects of the specific architecture of the tDED helix; firstly, that maintaining the DED2 domains aligned on Strands 1 and 2 is critical for Caspase-8 activity. Secondly, that Strand 3 has a scaffold role in maintaining the correct helical attributes, via Type II and III inter-strand interactions, confirming that all three types of interactions are essential to ensure the triple helix is formed with the correct molecular architecture. In the context of the DISC for example, we propose that upon death receptor activation and recruitment of the adapter protein FADD a Caspase-8 molecule is recruited via a Type I interaction. However, in order to drive Caspase-8 activation and cell death the triple tDED helix needs to form and simultaneously extend all three strands; this ensures that the catalytic domains of two Caspase-8 molecules are correctly aligned such that they are able to dimerize in an anti-parallel orientation forming the active site.

Additional tDED proteins can be recruited into the FADD:Caspase-8 complex and in many instances have been shown to alter the function/activity of the complex^30,31^. In this study, we have investigated the effect of c-FLIP_S_ recruitment on the structural architecture of the complex. As with the FADD:Caspase-8 complex, we observed a broad peak following density gradient purification, again suggesting that the FADD:Caspase-8:c-FLIP_S_ complex is very dynamic and highly heterogeneous. To aid analysis, we focused on a single fraction within the peak and found by negative-stain EM that the complexes were significantly smaller, although had a similar width to the FADD:Caspase-8 filaments characterized previously. Immuno-gold labeling revealed that multiple Caspase-8 molecules were recruited into each complex, whereas c-FLIP_S_ appeared to be at one/both ends of the complex. This is consistent with the fact that although c-FLIP_S_ is known to bind weakly to FADD, it is recruited primarily via co-operative binding to Caspase-8^24^. Taken together with the 2D class averages, these data suggest that, although a FADD:Caspase-8 filament is initiated, when c-FLIP_S_ is recruited to one of the strands within the helix the filament is terminated and the architecture of the c-FLIP_S_ complex is altered.

While exploring the mechanism of c-FLIP_S_ recruitment to FADD:Caspase-8, we identified through a combination of in silico docking and structure-guided mutagenesis a non-canonical binding site, via which c-FLIP_S_ DED1 interacts with Caspase-8 (Fig. 6). Computational modeling of c-FLIP_S_ recruitment via this site onto either of the two aligned Strands 1 or 2 of the Caspase-8 triple helix results in steric hindrance of the adjacent strand; crucially, this would stop recruitment of additional Caspase-8 molecules to further extend the helix. Furthermore, due to the alteration in the orientation of the FL motif on DED2 of c-FLIP_S_ as a result of binding via the DED1 non-canonical binding site, no more Caspase-8 molecules can be recruited to the c-FLIP_S_-containing strand of the helix. We, therefore, propose that this is one mechanism by which c-FLIP_S_ functions to terminate DED filament assembly and inhibit Caspase-8 activity. It is also clear from our data that, as a result of c-FLIP_S_ binding and termination of the FADD:Caspase-8 filament, the catalytic domains of the Caspase-8 are not positioned in an active conformation as a Caspase-8 catalytic dimer has not formed (Fig. 7; Supplementary Movie 2). This provides a hitherto unidentified explanation to explain the profound decrease in Caspase-8 activity observed when c-FLIP_S_ is recruited into the DISC^24,32^. The failure of Caspase-8 catalytic domains to correctly align in the presence of c-FLIP_S_ is most likely due to interruption of the long-range helical filament topology, which in turn prevents correct positioning of Caspase-8 catalytic domains for dimerization and enzymatic activation. Alternatively, it could be due to a non-structural constraint such as post-translational modifications as a result of c-FLIP_S_ binding, however further work would be required to explore this.

**Fig. 8:**
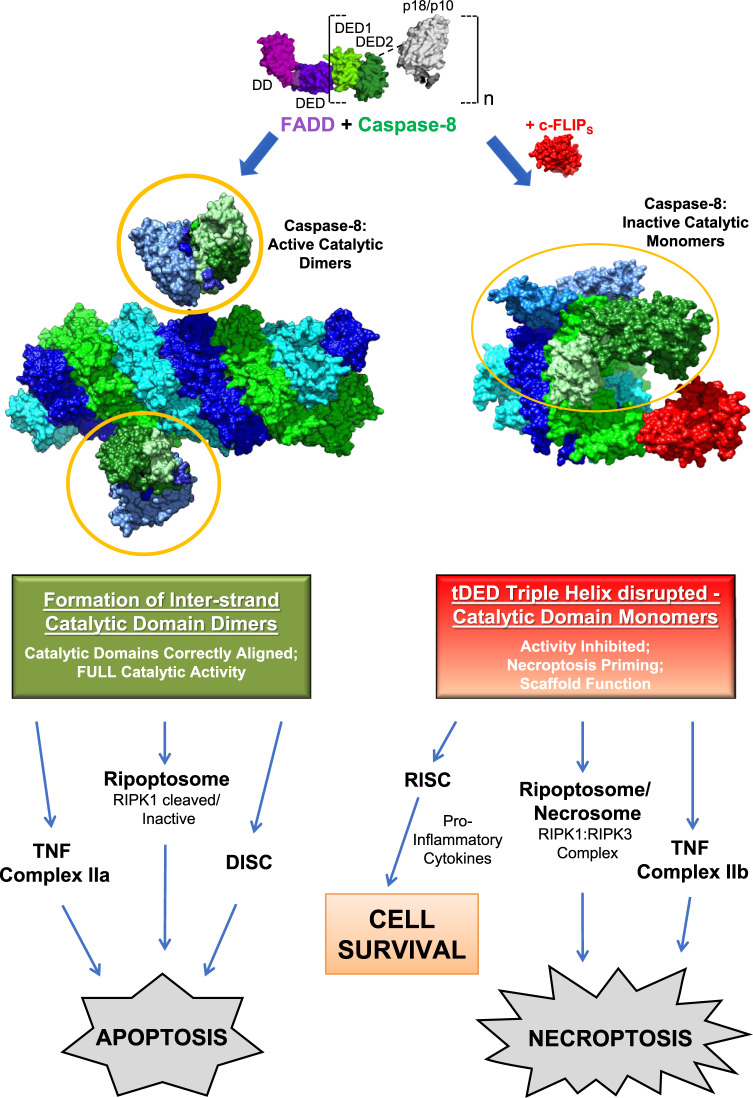
Cryo-EM structural analysis of FADD:Caspase-8 complexes defines the catalytic dimer architecture required for co-ordinated control of cell fate. FADD-nucleated complexes extend by sequential addition of Caspase-8 molecules, with all 3 strands within the tDED helix extending simultaneously. The filament extends in such a way that only the catalytic domains of Caspase-8 molecules on aligned strands of the triple helix (Strand 1, blue and Strand 2, green) are orientated correctly to enable antiparallel dimerization and full catalytic activation. The resultant catalytically active FADD:Caspase-8 complex triggers apoptosis via several key signaling platforms, namely TNF Complex IIa, the Ripoptosome and the DISC (Death-Inducing Signaling Complex). Recruitment of the tDED regulator, c-FLIP_S_, to the FADD:Caspase-8 complex significantly alters the molecular architecture of the complex and inhibits Caspase-8 enzymatic activity via a hitherto unknown dual mechanism. A non-canonical interaction of c-FLIP_S_ tDEDs within the helical filament alters the orientation of the aligned strands, thus preventing recruitment of further Caspase-8 molecules to adjacent strands and causing tDED filament termination. Due to the altered topology of the complex, the catalytic domains of Caspase-8 cannot align correctly or dimerize and therefore remain as inactive monomers. This signifies a pivotal switch in the canonical role of Caspase-8, whereby Caspase-8 instead functions as a scaffold for complexes signaling for RIPK1/RIPK3-driven necroptosis (via the Ripoptosome, Necrosome and TNF Complex IIb) or alternatively promotes cell survival, e.g., via the RISC (Receptor-Induced Signaling Complex).

Given that the core tDEDs of c-FLIP_S_ and c-FLIP_L_ are identical, the additional DED interaction interface we have identified here will be critically important in order to understand c-FLIP_L_ binding to the DED filament. This then raises the intriguing question as to the role this non-canonical interface may play in enabling the proposed heterodimerization of c-FLIP_L_ pseudocaspase domain with Caspase-8 catalytic domain; a process we and others have shown can either stabilize or inhibit caspase-8 activity, depending on the c-FLIP_L_/Caspase-8 ratio^24,33–36^. Importantly, in addition to its key role in apoptosis/necroptosis, Caspase-8 has a survival role as well as a recently reported inflammatory role that appears dependent on its ability to oligomerize^37^. The profound changes in the molecular architecture of c-FLIP_S_-containing complexes reported here therefore provide insights into how incorporation of tDED proteins, and in particular c-FLIP_L/S_, modulates Caspase-8 activation, thereby promoting either apoptosis (via Caspase-8:c-FLIP_L_ heterodimer)^38–40^, necroptosis (Caspase-8:c-FLIP_S_)^40,41^, or in some settings cell survival (c-FLIP-inhibited Caspase-8 still retains Caspase-8 ‘scaffold function’)^41–46^ (Fig. 8).

The model we now propose for initiation and extension of the FADD:Caspase-8 filament is that upon nucleation of complex formation initiated by FADD, the 3 strands within the tDED helix extend simultaneously (Fig. 8). This mechanism provides an additional level of regulation before a cell is committed to death. The requirement for recruitment of a minimum number of Caspase-8 molecules to ensure two Caspase-8 catalytic domains are correctly orientated to support antiparallel activation, prevents aberrant Caspase-8 activation. Once the filament has achieved the minimum length, the catalytic domains on the aligned Strands 1 and 2 of the triple helix are able to dimerize and become activated. By contrast, recruitment of c-FLIP_S_ to the FADD:Caspase-8 complex significantly alters the molecular architecture of the complex. Importantly, we identify that the interaction pocket of c-FLIP_S_ DED1 into which the FL motif of Caspase-8 DED2 binds includes additional interaction residues. This non-canonical interaction interface in c-FLIP_S_ DED1 alters the orientation of c-FLIP_S_ tDEDs within the helical filament. Consequently, c-FLIP_S_ functions to inhibit Caspase-8 activity by at least two mechanisms; first, by preventing recruitment of Caspase-8 molecules to adjacent strands of the helical filament thereby causing filament termination and second, by altering the topology of the complex thereby preventing dimerization of Caspase-8 catalytic domains. These profound changes in the molecular architecture of c-FLIP_S_-containing complexes not only provide insights to explain c-FLIP regulation of FADD:Caspase-8, but also how the incorporation of other tDED proteins could result in similarly significant changes to the architecture and signaling output of FADD:Caspase-8 complexes involved in the determination of cell fate. Crucially, since efforts are currently underway to target c-FLIP_L/S_ therapeutically using small molecule inhibitors, our findings also provide the molecular basis for targeting previously unidentified structural interactions.

## Methods

### Materials

Media and serum were purchased from Invitrogen (Paisley, UK). Antibodies were sourced as follows: FADD mouse monoclonal antibody (mAb)(610400, 1:500) was from BD Transduction Laboratories; Myc mouse mAb (2276, 1:1000) was from Cell Signaling; Caspase-8, p18-specific mouse mAb (AG-20B-0057-C050, 1:500) and c-FLIP mouse mAb (AG-20B-0056-C050, 1:500) were from Adipogen Corp. Caspase-8, N-terminal specific mouse mAb (04-574, 1:500) was from Millipore. Caspase-10 mouse mAb (M059-3, 1:1000) was from MBL. Vinculin mouse mAb (ab130007, 1:1000) was from Abcam. Anti-FLAG M2 mouse mAb (F3165, 1:1000) was from Sigma Aldrich. HRP-conjugated goat anti-mouse secondary antibody (A8924, 1:2000) was from Sigma Aldrich and HRP-conjugated goat anti-rabbit secondary antibody (P0448, 1:2000) was from DAKO. The Caspase-8 substrate, Ac-Ile-Glu-Thr-Asp-amino-4-trifluoromethyl coumarin (Ac-IETD.AFC) was from MP Biomedicals. All other chemicals were of analytical grade and obtained from Sigma-Aldrich or Fisher.

### Expression constructs

Full-length FADD, Caspase-8 and c-FLIP_S_ were cloned into pcDNA3 vectors with or without a non-cleavable 1xFLAG Tag. The constructs were co-transfected into HEK293F cells with polyethylenimine (PEI; Sigma) and harvested after 16 h^47^. GST-TRAIL-R1/R2-IcD and CD95-IcD were cloned into pGEX4T (GE Healthcare) and their respective fusion proteins produced in *E coli*^2,48^. Full-length FADD was cloned into the vector pTYB1 (NEB Inc.) and recombinant FADD (r-FADD) generated in *E coli*^19^. Full-length procaspase-8b (MACHα2/Mch5b) was cloned in pcDNA3.1 (Invitrogen) and untagged proteins produced by in vitro transcription/translation (IVT) (Insect System; Qiagen), incorporating ^35^S-methionine (GE Healthcare)^19^. Mutations were made using the Stratagene QuikChange Site-Directed Mutagenesis kit and confirmed by DNA sequencing. Proteins from c-FLIP_S_·Myc in pcDNA6.1 were produced by IVT (TNT T7-coupled reticulocyte lysate system; Promega).

### Cell culture

Adherent HEK293H and suspension HEK293F cells (both from Invitrogen), were maintained in FreeStyle 293 F or DMEM medium, supplemented with 10% fetal bovine serum (FBS). Cells were grown in an atmosphere of 10% CO_2_ in air at 37 °C (for 293F cells, shaking at 120 rpm) and maintained in logarithmic growth phase by routine passage every 3–4 days. Caspase-8-deficient Jurkat T cells (gift from J Blenis) were cultured in RMPI media, supplemented with 10% FBS and 2 mM Glutamax, and maintained in 5% CO_2_ at 37 °C.

### Generation of CRISPR/Cas9 Caspase-8-null 293F cells

Caspase-8-deficient 293F cells were generated using the CRISPR-Cas9 system. 293H cells were transiently transfected using Lipofectamine™ 3000 Transfection Reagent (Thermo Fischer Scientific, Waltham, MA, USA) according to manufacturer’s instructions. gRNAs were inserted into the all-in-one guide RNA/Cas9 expression vector, pLEIC-97 (Protein Expression Laboratory (PROTEX), University of Leicester). gRNA sequences (Supplementary Table 2) were used as described in^49^ to target the 5’ end of the gene, and thus all isoforms of Caspase-8. One-day post-transfection, puromycin was added for two days to select for transfected cells. Single clones were isolated, analyzed for successful Caspase-8 knockout and adapted for suspension culture.

### DISC reconstitution

Complete reconstitutions were carried out as described^19^. Briefly, glutathione sepharose beads coated with purified GST-CD95-IcD or GST-TRAIL-R1/R2-IcD (10 µg)^2,48^ were incubated with recombinant FADD (r-FADD; 5 µg), and the indicated amounts of IVT-produced wild-type or mutant procaspase-8b (^35^S-labeled) for 16 h at 20 °C. Control reconstitutions contained glutathione sepharose beads coated with GST-alone. Bead-associated complexes were analyzed by SDS-PAGE/Western blotting. Reconstituted DISC-associated Caspase-8 cleavage activity was measured using the fluorogenic substrate Ac-IETD.AFC (40 µM) at 37 °C on a Victor X4 fluorimeter.

### Western blot analysis

Proteins were resolved by SDS-PAGE using the Mini-Protean III or Criterion TGX Precast gel systems (Biorad Laboratories) and transferred to ‘Hypond C Extra’ nitrocellulose membrane (GE Healthcare) using the Mini-Trans Blot system (Biorad Laboratories). Blots were then probed with the indicated primary antibodies, followed by the appropriate HRP-conjugated secondary antibody. Immunolabelled proteins were visualized using ECL enhanced chemiluminescence (GE Healthcare) according to the manufacturer’s instructions.

### Transient co-transfection of Caspase-8 mutants with GFP and analysis of cell death

For co-transfection of catalytically active Caspase-8 wild-type or Type I/II/III mutants with GFP, Caspase-8-deficient Jurkat cells were plated 24 h prior to transfection with a 5:1 ratio of Caspase-8: EGFP, using PEI (Sigma). 24 h post-transfection, cells were treated with or without ant-CD95 (clone CH11; Cell Signaling) for 4 h and cell death measured by FACS analysis. By using EGFP as a transfection marker, cell death in transfected cells was specifically quantified on a BD FACSCanto II flow cytometer using Annexin V-APC (Cambridge Bioscience) and Draq 7 co-labeling and gating of GFP-positive cells only. Data were analyzed using BD FACS Diva v8.0.01; Annexin V labeling was used as a marker of early apoptotic cells, double-labeled Annexin V/Draq 7 cells represented late apoptotic cells and double-negative cells represented viable cells. Graphical representation of FACS sequential gating strategy is provided in Supplementary Fig. 21.

### Quantitative mass spectrometry

LC-MS/MS was used to quantify the proteins present in the purified FADD-nucleated Caspase-8 complexes. Purified protein complexes were separated using 30–70% glycerol density gradients. Proteins in each gradient fraction were precipitated using the UPPA protein precipitation kit (gBioscience) according to the manufacturer’s Instructions. The precipitated proteins were re-suspended and then digested with trypsin. Extracted tryptic peptides were concentrated to dryness and re-suspended in 5% formic acid (FA) and acetonitrile (9:1), spiked with 20–40 fmol/µl ADH1 and BSA MassPREP standards (Waters Corporation, Manchester, UK). Aliquots (2–4 µl) were applied to a reverse-phase BEH130 C18 column (25 cm × 75 µm × 1.7 µm I.D.) using a Waters nanoAcquity UPLC system interfaced to a Synapt G2-S HDMS mass spectrometer. Peptides were eluted (0.3 µl/min) with 50 min, 3–40 % (0.1% FA/acetonitrile) gradients and analyzed in data-independent acquisition (DIA) and ion mobility (HDMS^E^) modes using a T-wave velocity of 650 m/s^50^. Stepped 4 eV and 20–50 eV voltage switching generated collision induced (CID) peptide fragmentation. Low energy and CID LC-MS/MS data were acquired (1 s cycle scan time and 50–2000 m/z mass range) and processed using Waters ProteinLynx Global SERVER (PLGS 3.0) using the UniProt Human database (UniProtKB release 2014_11, 20,265 entries). Peptide mass and fragment mass tolerances were set to auto, with one missed cleavage and variable modifications for methionine oxidation and carbamidomethylation of cysteines. The PLGS “TOP 3” method with a false discovery rate of 1% was used for absolute protein quantification^24,51^.

### Preparation of FADD:Caspase-8 and FADD:Caspase-8:c-FLIP_S_ complexes

Caspase-8 (C360A) and FLAG-FADD or Caspase-8 (C360A), FADD and FLAG-c-FLIP_S_ were co-expressed in Caspase-8-deficient HEK 293F cells and purified using FLAG-affinity beads followed by 30–70% glycerol density gradient purification. Single fractions from the glycerol gradients (centrifuged using SW60Ti rotor 125,812 × *g* at 4 °C for 17 h) were analyzed by Western blotting and negative stain and/or Cryo-electron microscopy.

### Grid preparation for negative-stain and cryo-electron microscopy

Purified protein complexes were chemically fixed and the glycerol concentration diluted by mixing gradient fraction with equi-volumes of 2% (negative stain) or 0.1% (Cryo) glutaraldehyde in 150 mM NaCl, 20 mM HEPES-KOH (pH7.4) buffer. Negative staining was carried out according to conventional procedures. Briefly, a small amount (3 µl) of the sample was loaded onto carbon-coated grids (300 mesh gold, EM resolutions Ltd, Sheffield UK), which had been glow-discharged for 30–60 s at 30 mA using a GloQube instrument (Quorum Technologies Ltd., East Sussex UK). After incubation at 5 °C overnight, the excess volume was removed by blotting with filter paper. Grids were stained with two drops of 2.5 % (w/v) uranyl acetate solution for 60 s, which was filtered to remove aggregates and then blotted with filter paper to stop the staining and dry the grids. Anti-capillary self-crossover tweezers (N5AC, Dumont, Montignez Switzerland) were used for all procedures. For Cryo-EM, 3 µl fixed sample was applied to glow-discharged Quantifoil R1.2/1.3 gold mesh (Quantifoil Micro Tools GmbH, Germany) graphene oxide (Sigma-Aldrich)-coated holey carbon grids. After incubation for 1 min, excess volume was blotted with filter paper and sample-loading repeated 3X times prior to plunge-freezing using a Vitrobot (FEI).

### Immunogold-labeling of complexes

For immunogold-labeling of purified complexes, the samples were adsorbed on to the carbon-coated grids (300 mesh size, Gold). Protein samples were blocked with 2% or 0.2% BSA in HEPES buffer for 30 min at room temperature and specific proteins within the complexes were then labeled by incubating with anti-caspase-8 C15 mouse mAb (AG-20B-0056-C050, 1:100; Adipogen Corp.), anti-FLAG rabbit polyclonal (F7425, 1:100; Sigma-Aldrich), or anti-FLAG M2 mouse mAb (F3165, 1:100; Sigma-Aldrich) in 0.2 % BSA HEPES buffer for 2 h at RT in a moisture chamber. After washing with three drops of 0.2 % BSA-HEPES buffer, the grids were incubated with 5 nm or 10 nm gold-conjugates with anti-IgG antibody (EM.GAR5/10, EM.GAF5 or EM.GMHL10, 1:20; BBI Solutions, Newport UK) or protein-G (AC-5-18-05, 1:20; Cytodiagnostics Inc., ON Canada) in 0.2 % BSA HEPES buffer for 2 h at RT. After washing with 3 drops of HEPES buffer, specimens were stained with uranyl acetate.

### Negative-stain EM data collection and image processing

Negative stain TEM data were collected on well-dried specimens using a Talos electron microscope (FEI), operated at 200 kV acceleration. Images were recorded using the EPU system (Thermo Fisher Scientific, MA USA) at room temperature using a 4096 × 4096 pixel CMOS camera Ceta16M (FEI), at a magnification of x 57,000 (pixel size 1.83 Å), 1 s exposure per micrograph. FLAG-FADD complexes were manually picked from 620 micrographs yielding a dataset of 3200 particles, as summarized in Supplementary Table 1. The single-particle analysis was then performed using RELION^27,52^ and initial models generated and refined in CryoSPARC^53^, as summarized in Supplementary Figs. 4, and 7.

### Cryo-EM data collection and image processing

Cryo-EM data optimization and screening was performed on a Talos (200 kV using a Falcon 2 direct electron detector). Cryo-EM data collection was performed on a Titan Krios electron microscope (FEI) operated at an acceleration voltage of 300 kV using a Falcon 3 direct electron detector camera (FEI). Images were recorded with a pixel size of 1.08 Å, 1 s exposures at 93 electrons/Å^2^/sec dose rate, 39 fractions. A total of 534 images were used for analysis and processing. Motioncorr 2 was used for motion correction^54^ and GCTF was used for CTF estimation as implemented in RELION 3. FLAG-c-FLIP_S_:FADD:Caspase-8 complexes were initially picked using CrYOLO^26^, yielding a dataset of 12918 particles. Reference-free 2D Class averages were generated from these data in RELION 3 and 10 good classes then used as templates for auto-picking of particles in RELION 3, yielding a dataset of 46023 particles (Supplementary Table 1). The single-particle analysis was performed in RELION 3^27^, as outlined in the flowchart in Supplementary Fig. 17, resulting in a final post-processed EM map with a final resolution of ~13 Å.

### Atomic structure fitting

Docking was done using published structures; the Cryo-EM structure of Caspase-8 tDED filament (5L08;^12^), and the NMR structure of monomeric un-cleaved Caspase-8 catalytic domain (2k7z;^21^). A homology model of c-FLIP_S_, obtained using Phyre2^55^ was used for fitting into the FLAG-c-FLIP_S_:FADD:Caspase-8 reconstruction. Structures were automatically fitted as rigid-bodies into the generated EM maps using the UCSF Chimera package^56^.

### HADDOCK docking

HADDOCK 2.0 High Ambiguity Driven protein-protein DOCKing^57^, developed by Dominguez et al.^58^, was used for docking of c-FLIP_S_ DED1 with Caspase-8 DED2 as it enabled the use of experimental mutagenesis data to drive the sampling around the contact regions of the interacting proteins by introducing interaction restraints. This enabled new residues involved in the binding interface to be identified (Supplementary Fig. 16).

### Statistics and reproducibility

The number of independent experiments and of replicates (*n*) is indicated in the figure legends. All the Western blotting and micrograph data were repeated independently three times with similar results. Statistical analysis was performed using Prism 7 or 8 software (GraphPad).

### Reporting summary

Further information on research design is available in the Nature Research Reporting Summary linked to this article.

## Supplementary information

Supplementary Information

Description of Additional Supplementary Files

Supplementary Movie 1

Supplementary Movie 2

Reporting Summary

## Data Availability

Data supporting the findings of this manuscript are available from the corresponding authors upon reasonable request. A reporting summary for this Article is available as a Supplementary Information file. Source data are provided with this paper. Accession Codes for the EMDB datasets (http://www.wwpdb.org) are as follows: https://www.ebi.ac.uk/pdbe/entry/emdb/EMD-11938 https://www.ebi.ac.uk/pdbe/entry/emdb/EMD-11939 https://www.ebi.ac.uk/pdbe/entry/emdb/EMD-11940 https://www.ebi.ac.uk/pdbe/entry/emdb/EMD-11941 The mass spectrometry proteomics data have been deposited to the ProteomeXchange Consortium via the PRIDE partner repository (http://www.ebi.ac.uk/pride) with the dataset identifier PXD022408 and 10.6019/PXD022408. Source data are provided with this paper.

## Source data

Source Data
