# Supplementary material for: Cryo-EM structural analysis of FADD:Caspase-8 complexes defines the catalytic dimer architecture for co-ordinated control of cell fate
Source: Nat Commun. 2021 Feb 5;12:819. doi: 10.1038/s41467-020-20806-9 (PMC7864959; doi:10.1038/s41467-020-20806-9)
Supplement: Supplementary file 1 — Supplementary Information [file 41467_2020_20806_MOESM1_ESM.pdf]

Supplementary Information

Cryo-EM structural analysis of FADD:Caspase-8 complexes defines the catalytic dimer architecture for co-ordinated control of cell fate

Joanna L Fox[1,2*†], Michelle A Hughes[1], Xin Meng[1], Nikola A Sarnowska[1], Ian R Powley[1], Rebekah Jukes-Jones[1], David Dinsdale[1], Timothy J Ragan[2], Louise Fairall[2], John WR Schwabe[2], Nobuhiro Morone[1], Kelvin Cain[1] & Marion MacFarlane[1*†]

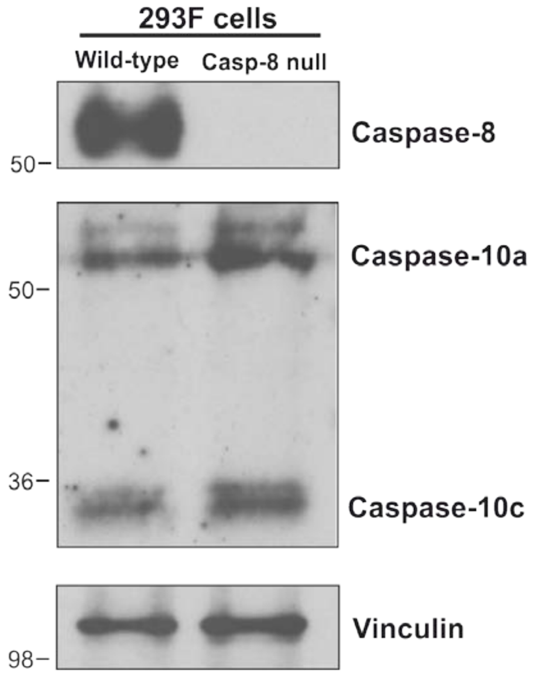

Supplementary Fig. 1: Representative western blot, from 3 independent experiments, showing expression of Caspase-8 and Caspase-10 in 293F parental cells (Wild-type) and Caspase-8 null cells (Casp-8 null;1D3). Vinculin served as loading control. These data confirm that 1D3 cells completely lack endogenous Caspase-8, but retain expression of endogenous Caspase 10a/c. Source data are provided as a Source Data file.

a)

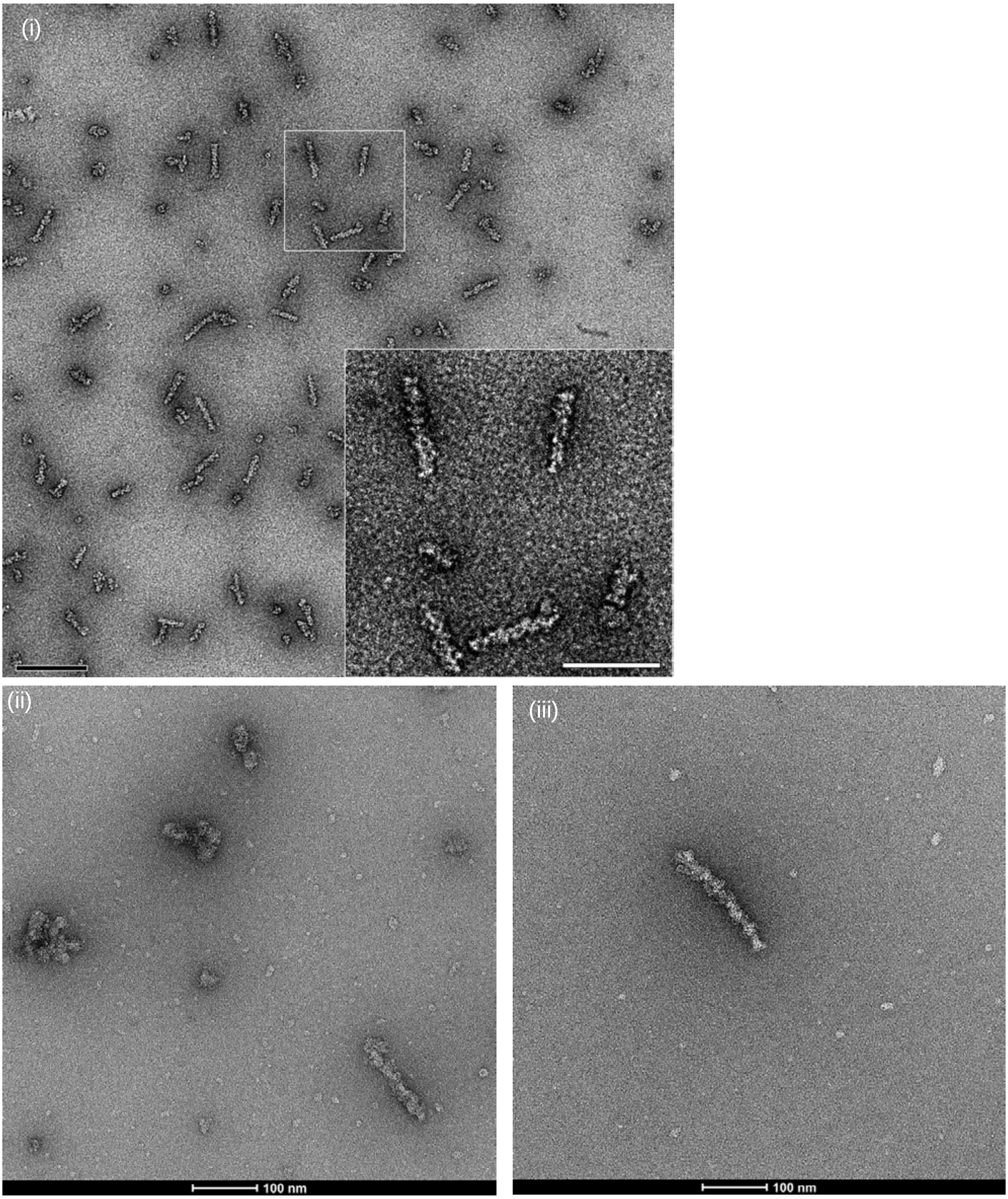

b)

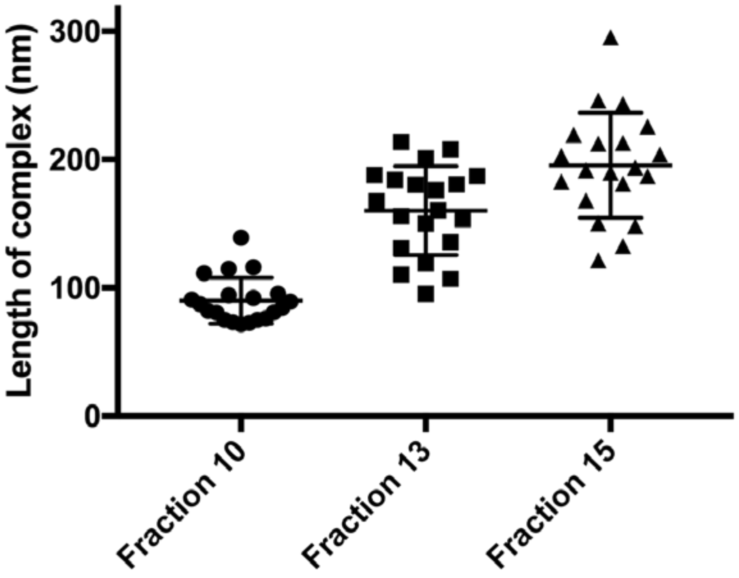

Supplementary Fig. 2: a) Representative micrographs, from 3 independent experiments, of Negative-stain complexes from glycerol density gradient fractions 10 (i)(Scale bar, 200 nm; inset, 100 nm), 13 (ii) and 15 (iii). These fields highlight the decreasing protein complex concentration across the glycerol density gradient fractions and heterogeneity within each fraction, with complexes of multiple sizes visible in each field. **b)** Quantitation of the length of 20 independent complexes from multiple micrographs within one representative experiment, for each glycerol density gradient fraction (10, 13 and 15), measured in nanometres (graph shows Mean ± SEM). Source data are provided as a Source Data file.

a)

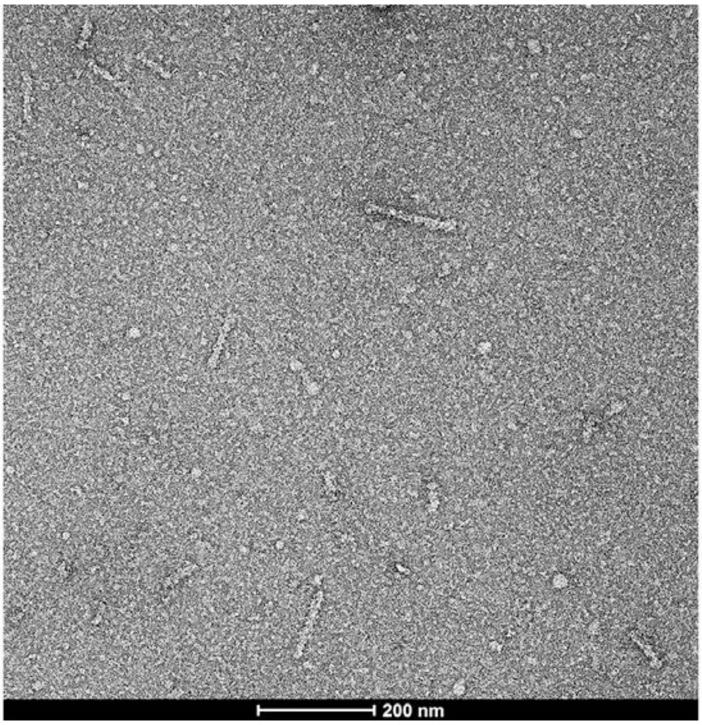

b)

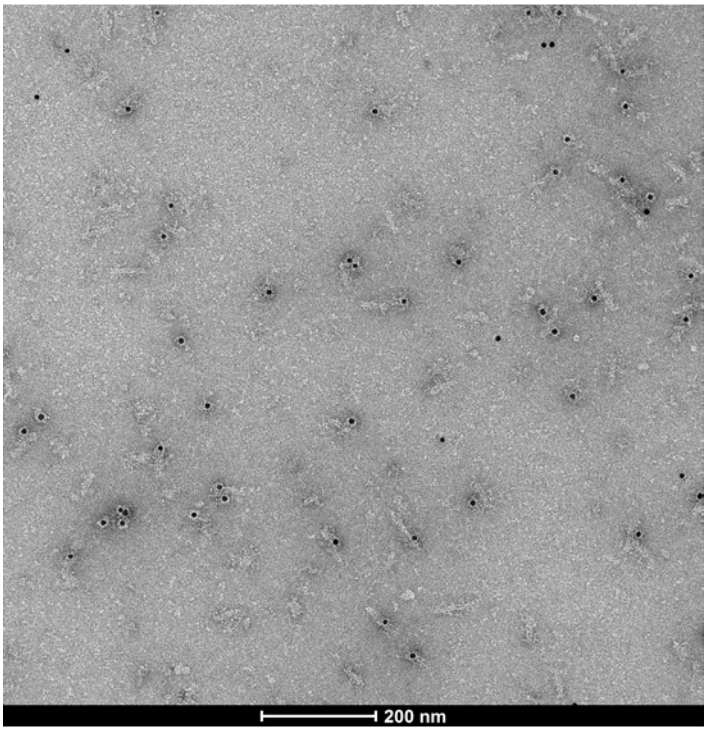

Supplementary Fig 3: a) Gold-tagged anti-IgG control for Immuno-gold labelling of FLAG-FADD:Caspase-8 Negative-stain complexes. Micrograph representative of 3 independent experiments. **b)** Representative micrograph, from 3 independent experiments, showing a field of Immuno-gold labelled FLAG tag on purified FLAG-FADD:full-length Caspase-8 complexes (Fraction 10), visualized by EM.

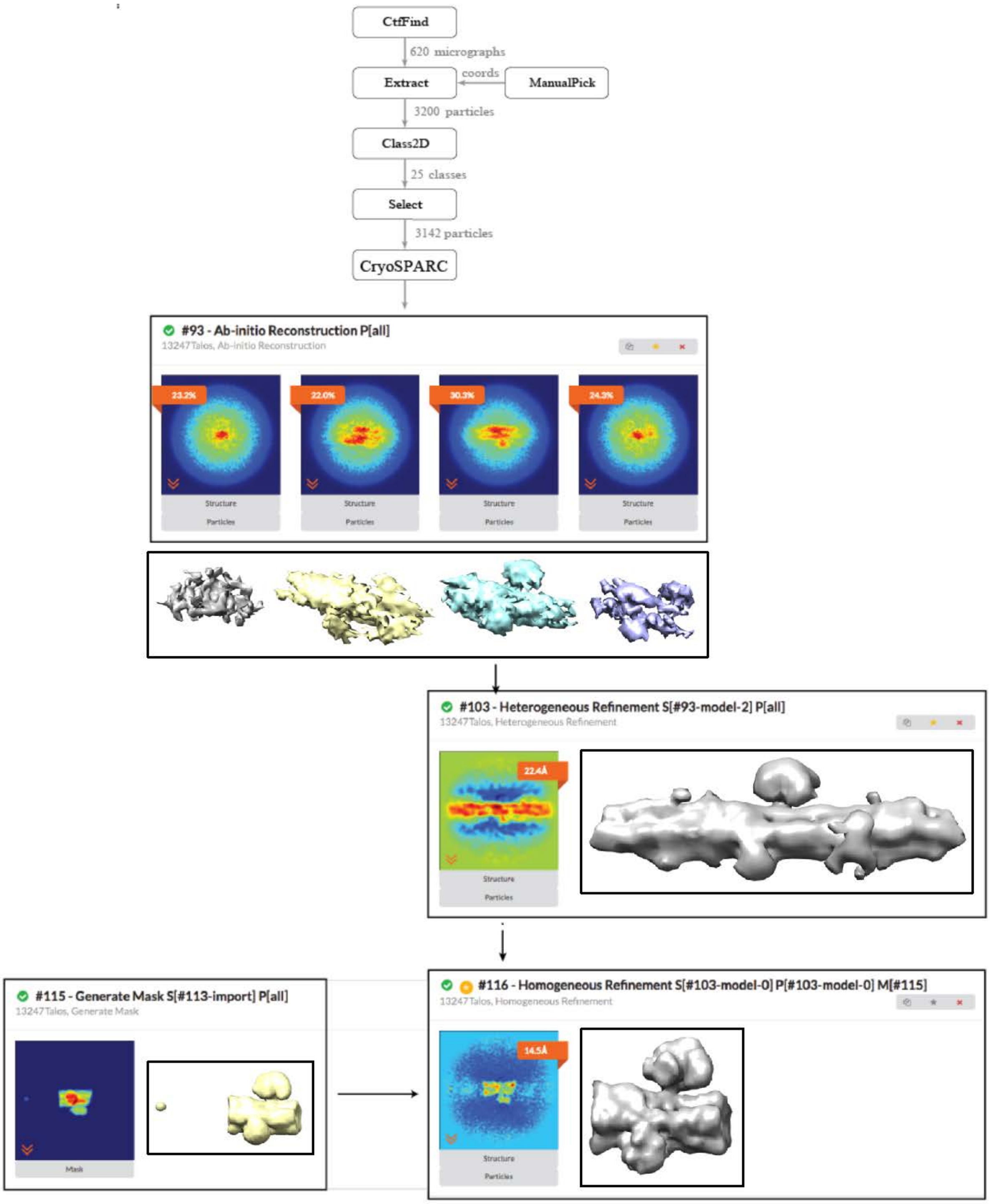

Supplementary Fig. 4: Flowchart of single particle analysis of Negative-stain dataset of FLAG-FADD:Caspase-8 complexes in RELION and CryoSPARC. 3D Volumes are shown in grey.

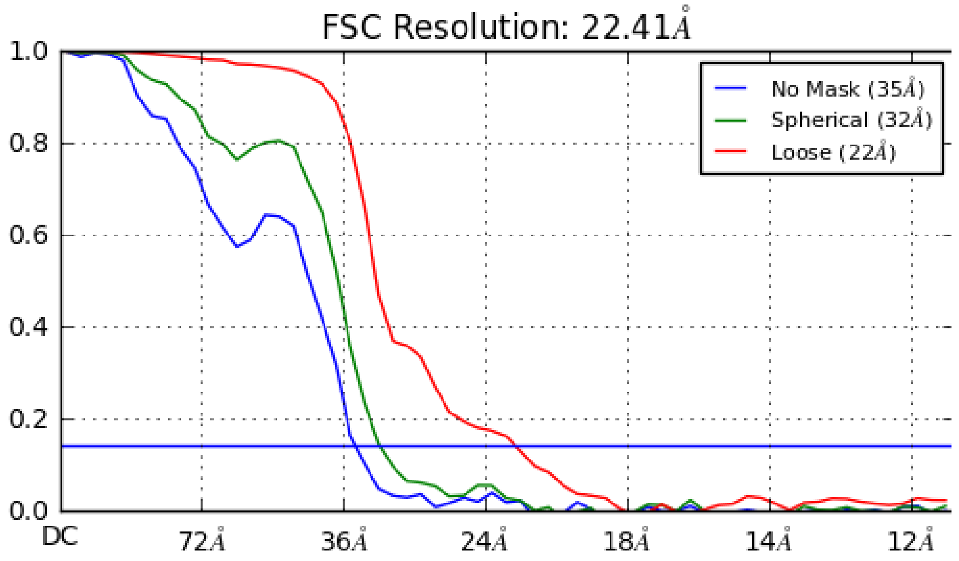

Supplementary Fig. 5: FSC Curve for Negative-stain dataset of FLAG-FADD:Caspase-8 complexes analysed using CryoSPARC v2.1.

a)

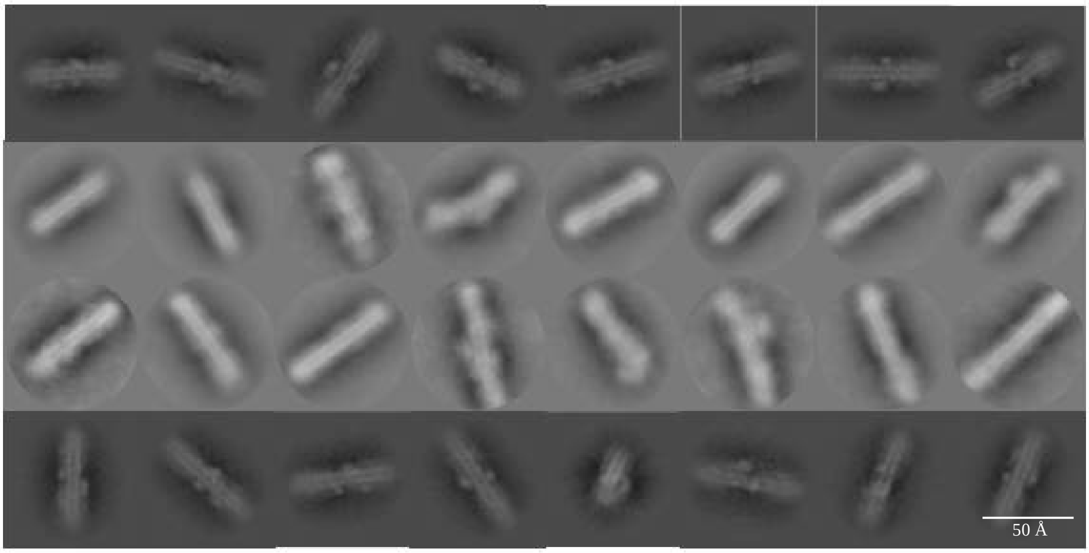

Supplementary Fig. 6: a) Comparison of negative-stain reference-free 2D class averages (central two rows), and 2-D re-projections of the 3D reconstruction in the corresponding orientation (top and bottom rows) of the FLAG-FADD:Caspase-8 complex.

b)

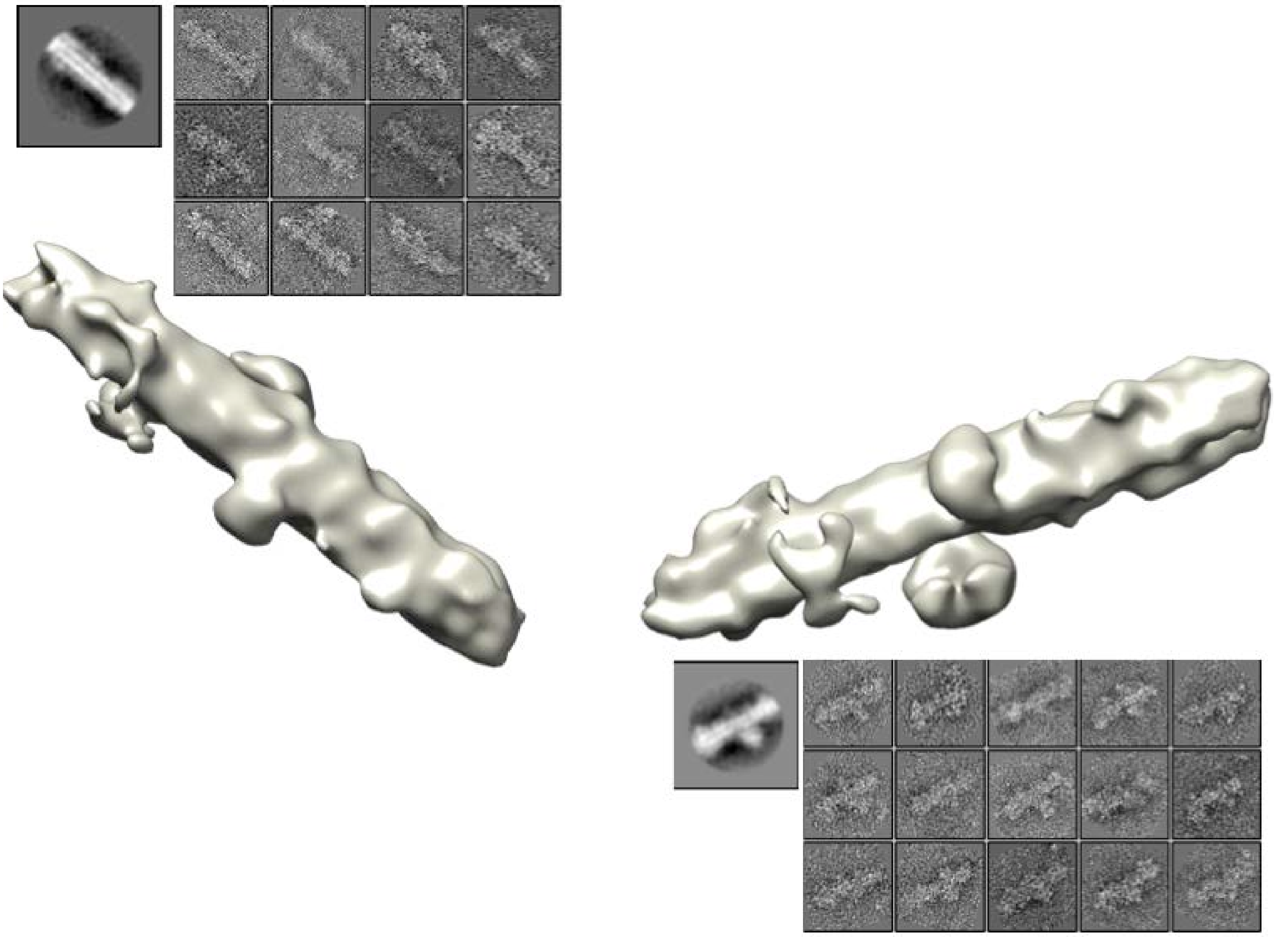

Supplementary Fig. 6: b) Comparison of representative negative-stain reference-free 2D class averages and 3D reconstruction with the individual particles in each class correctly orientated with the class average, highlighting key features in the FLAG-FADD:Caspase-8 complex.

c)

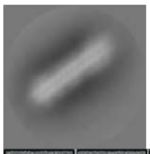

2D Class: 1

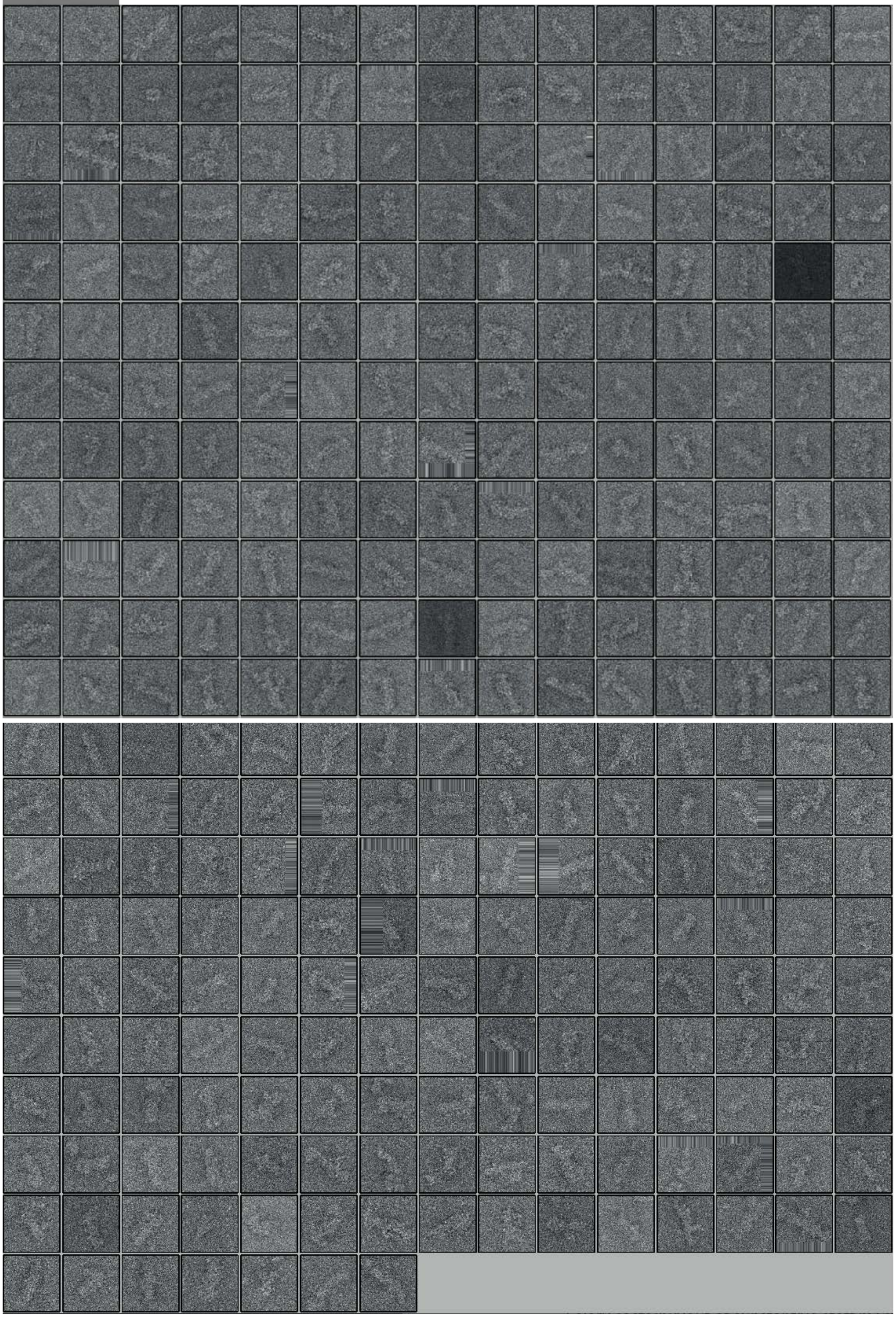

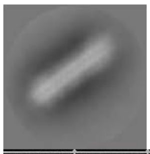

2D Class: 1, continued

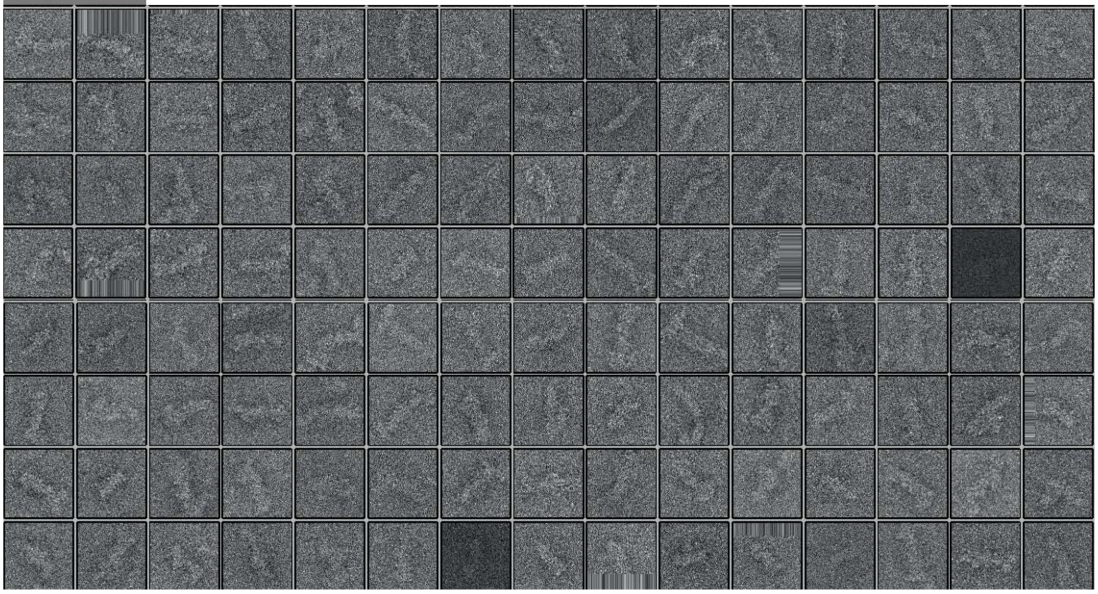

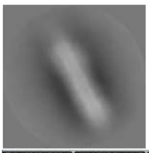

2D Class: 2

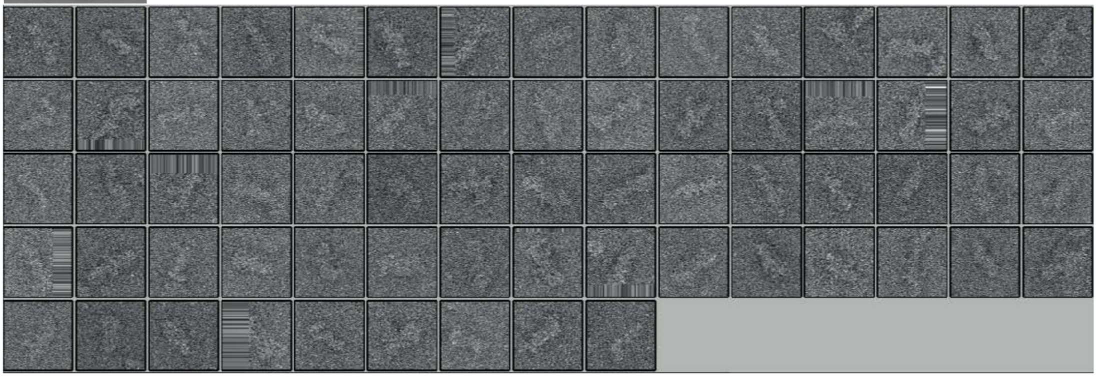

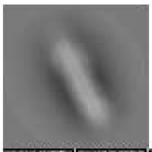

2D Class: 2, continued

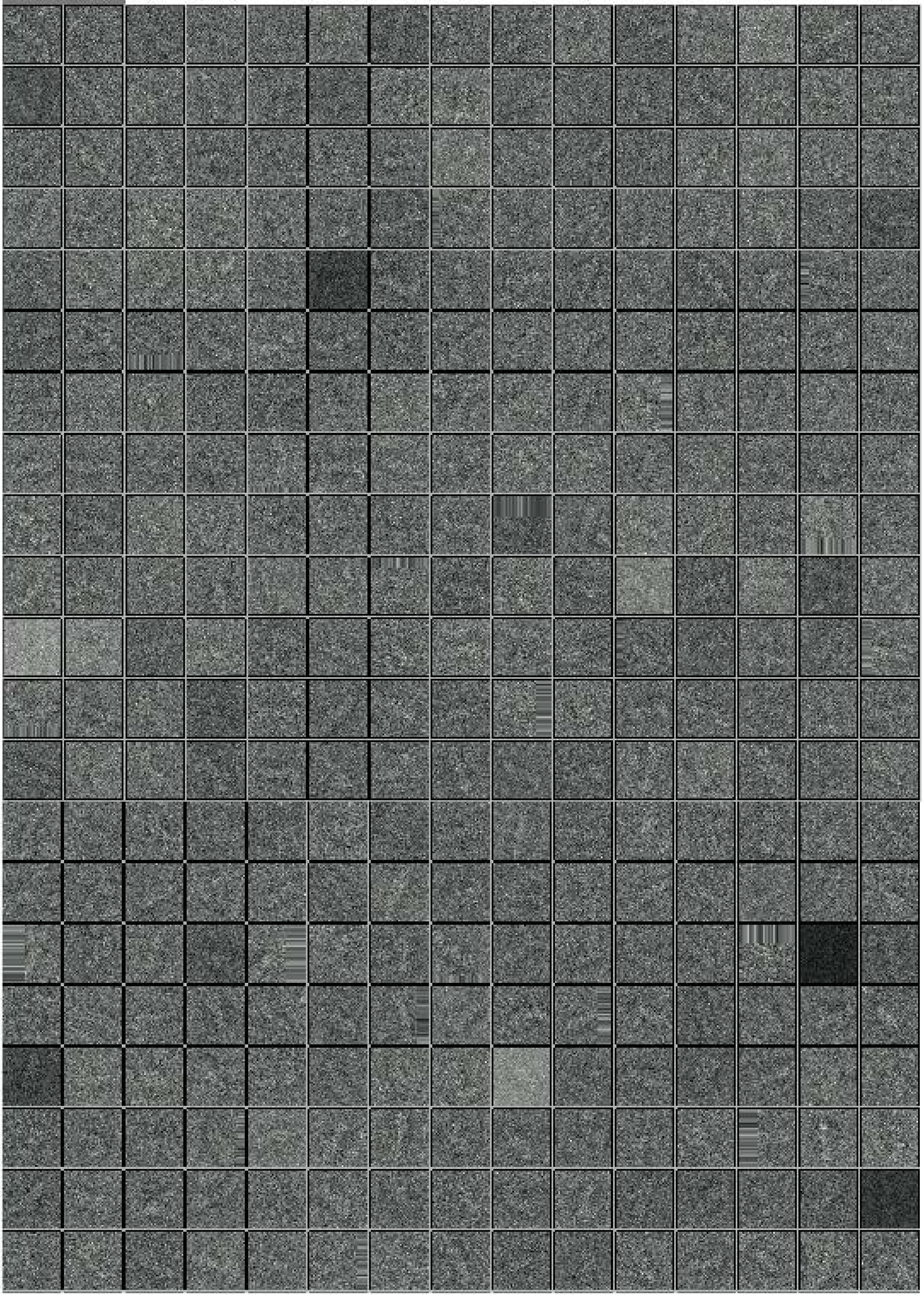

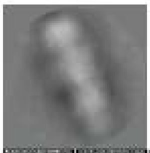

2D Class: 3

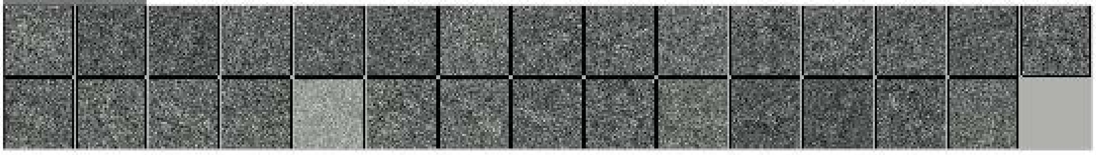

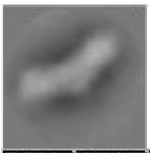

2D Class: 4

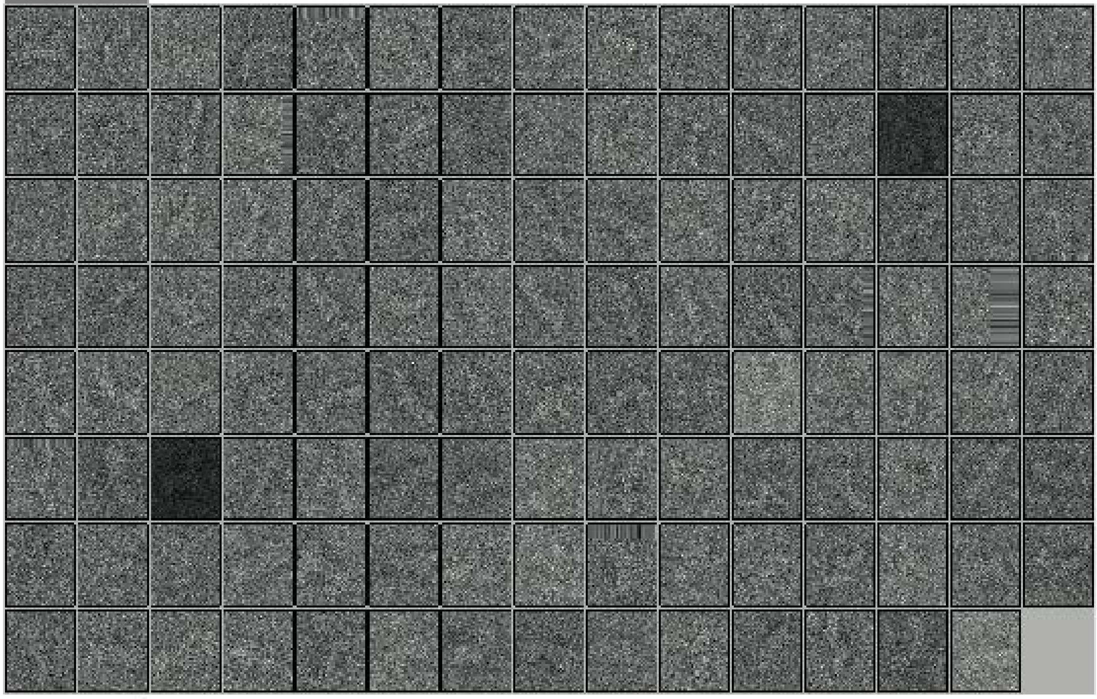

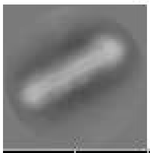

2D Class: 5

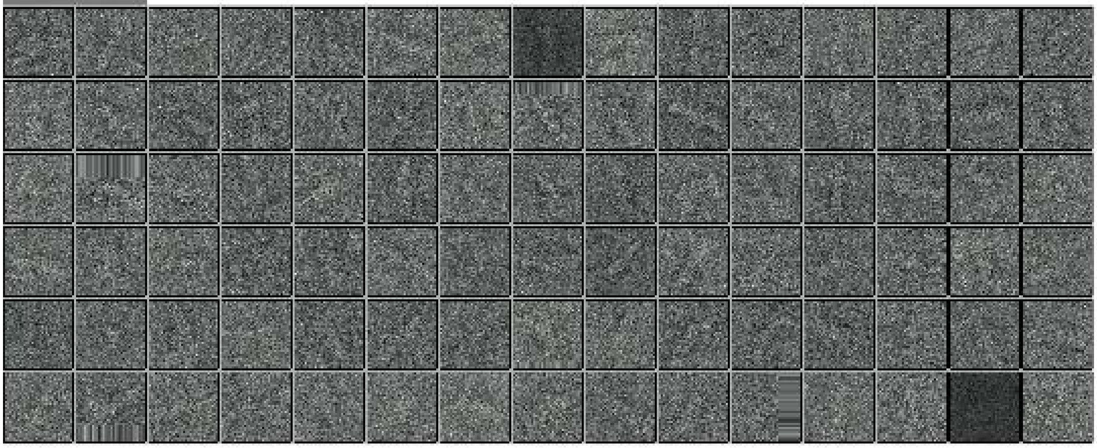

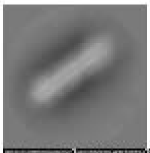

2D Class: 6

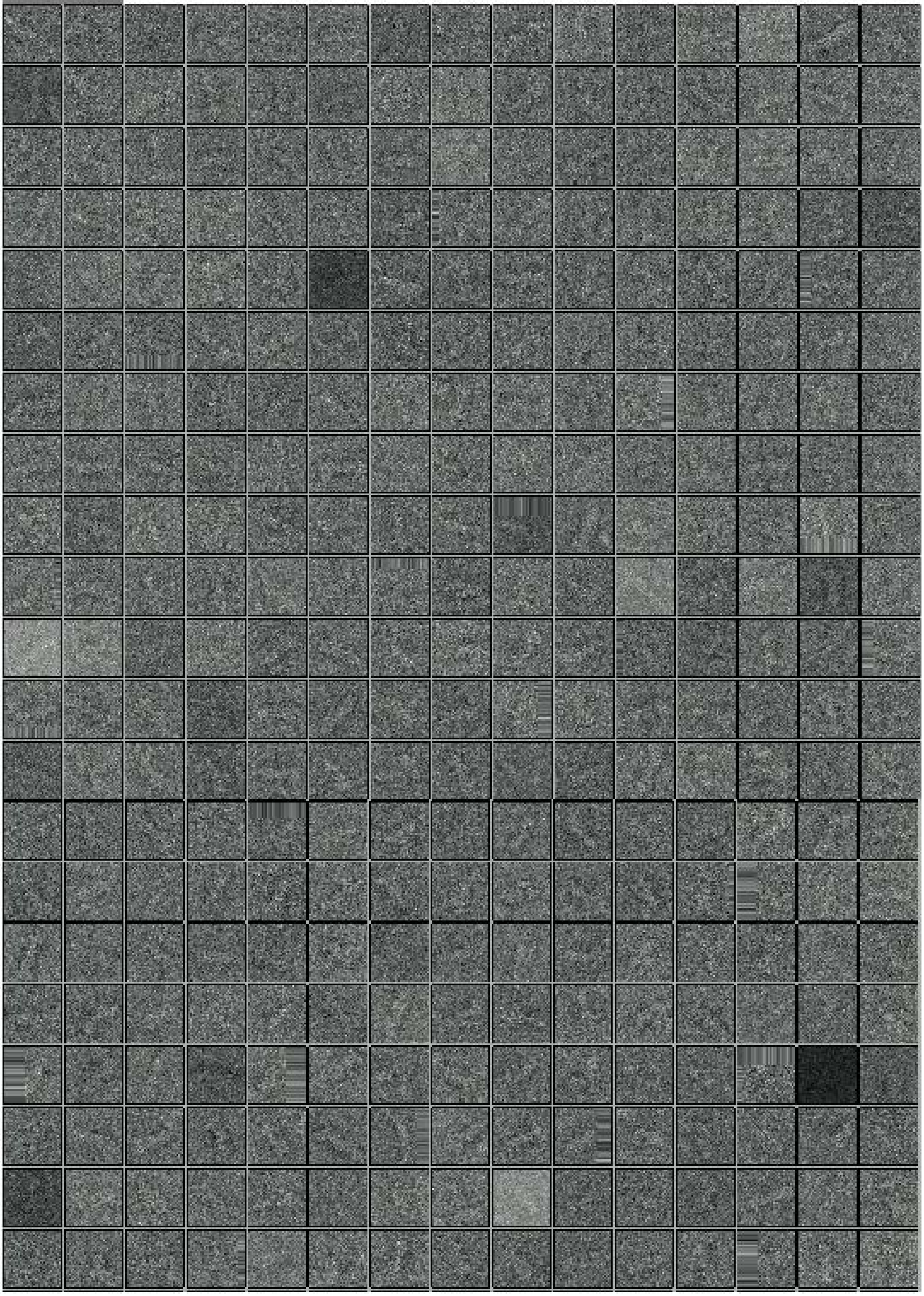

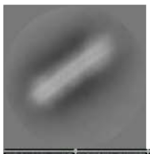

2D Class: 6, continued

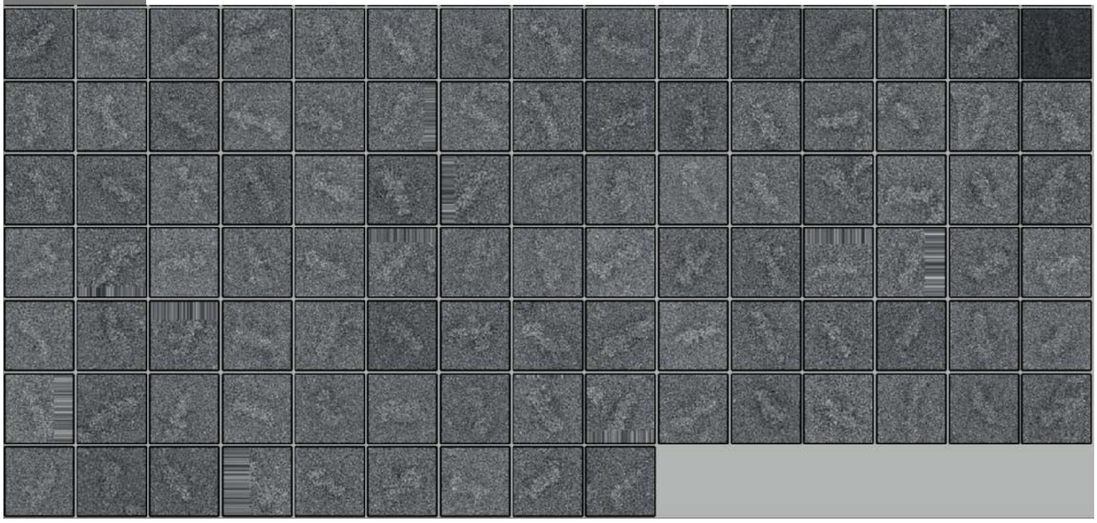

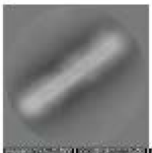

2D Class:7

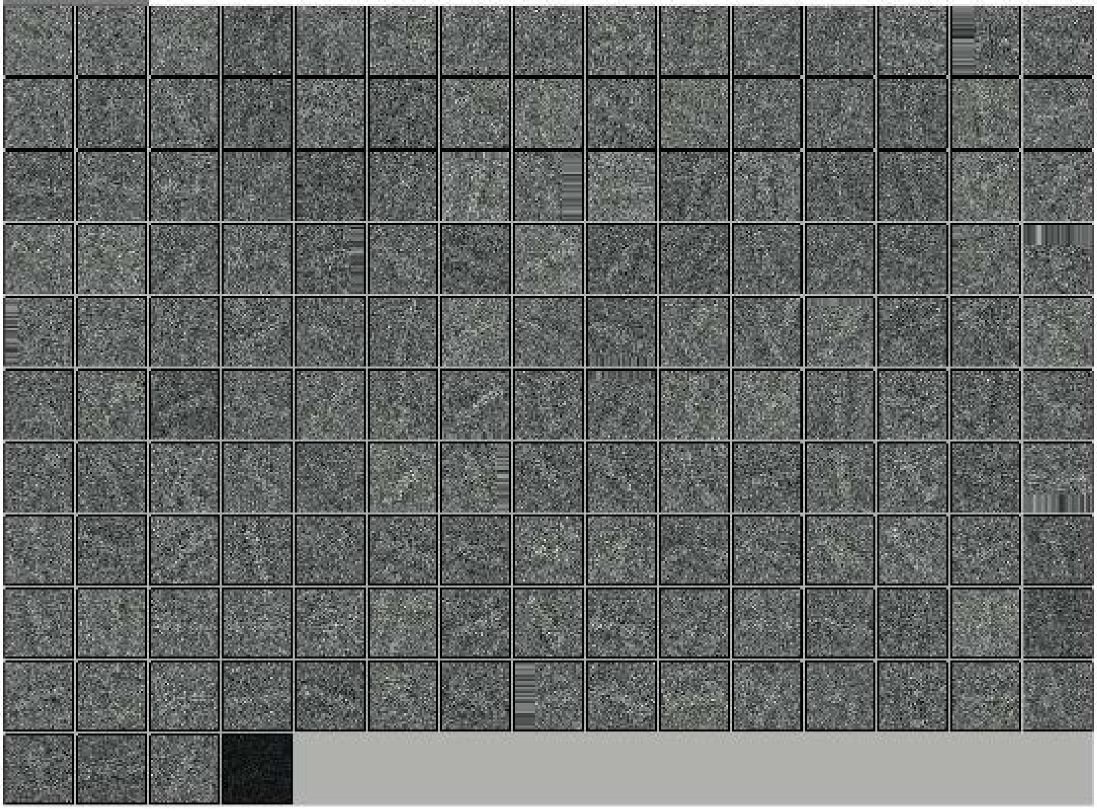

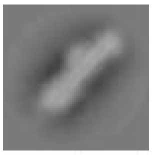

2D Class: 8

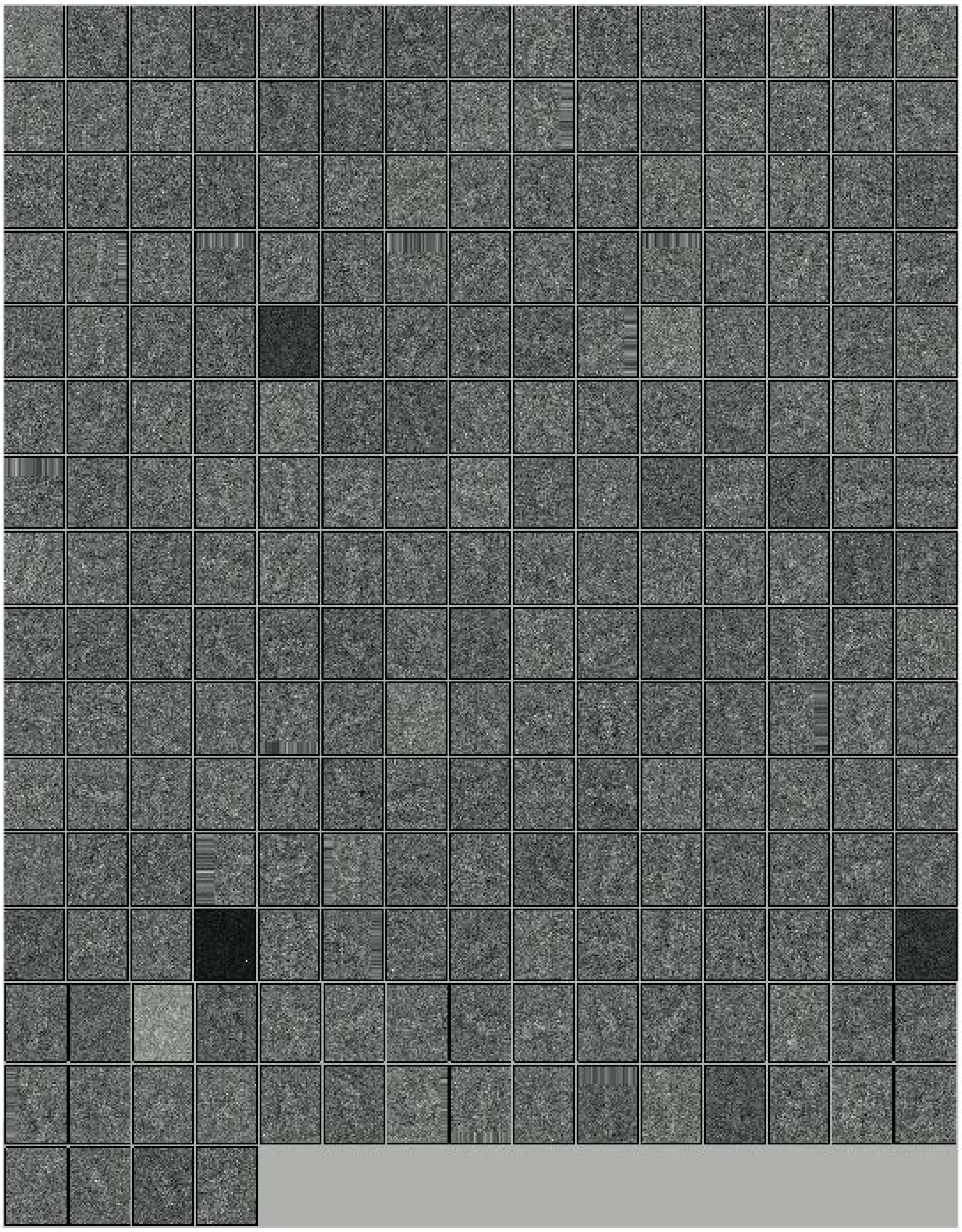

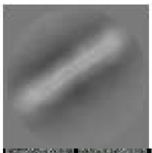

2D Class: 9

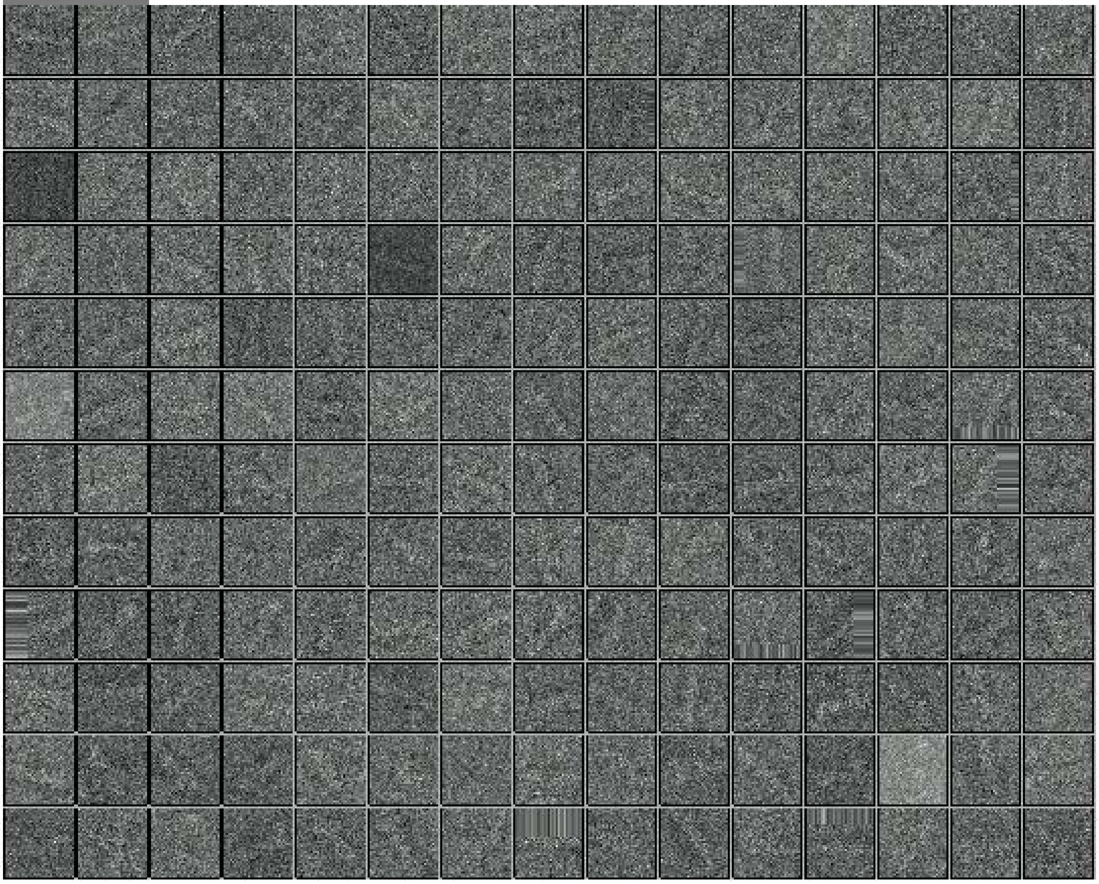

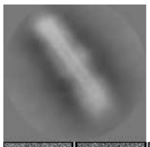

2D Class: 10

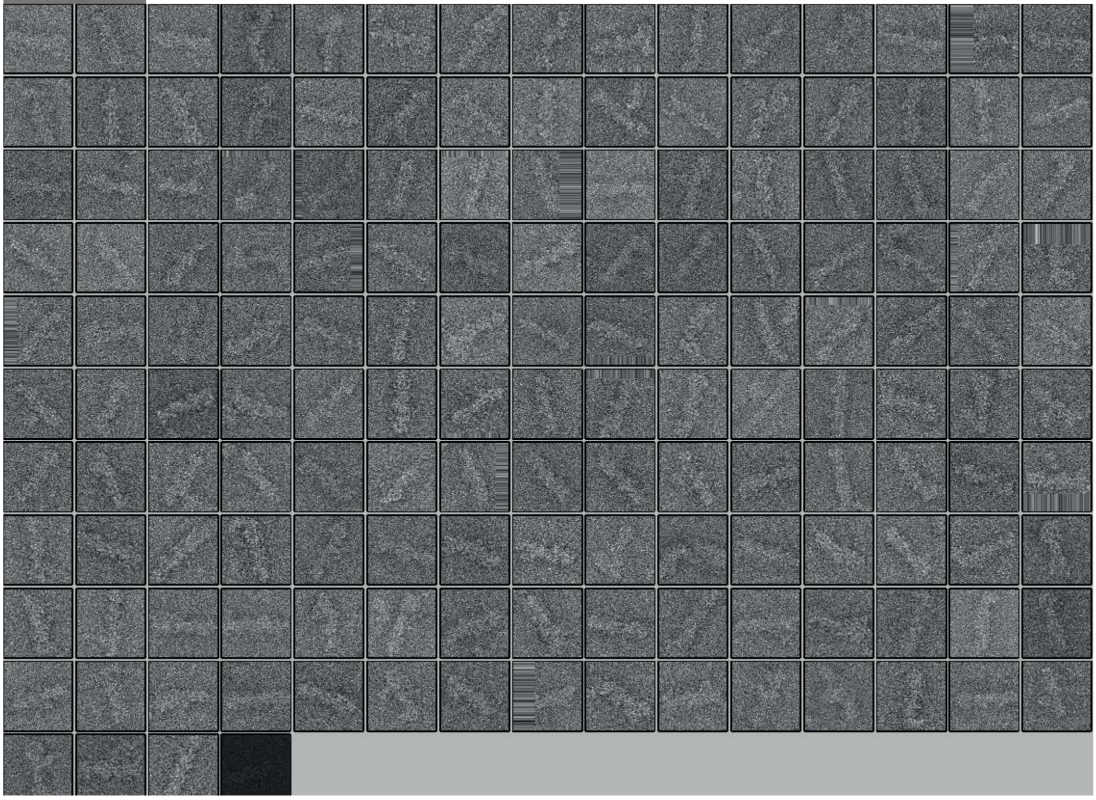

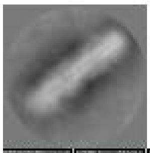

2D Class: 11

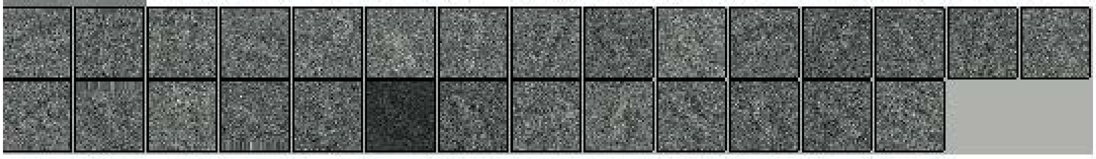

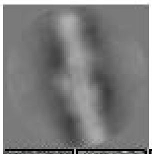

2D Class: 12

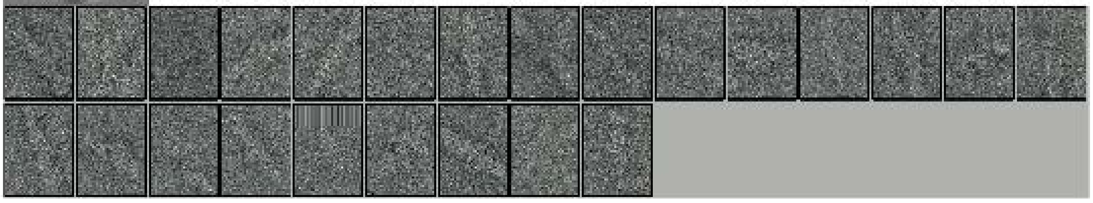

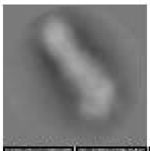

2D Class: 13

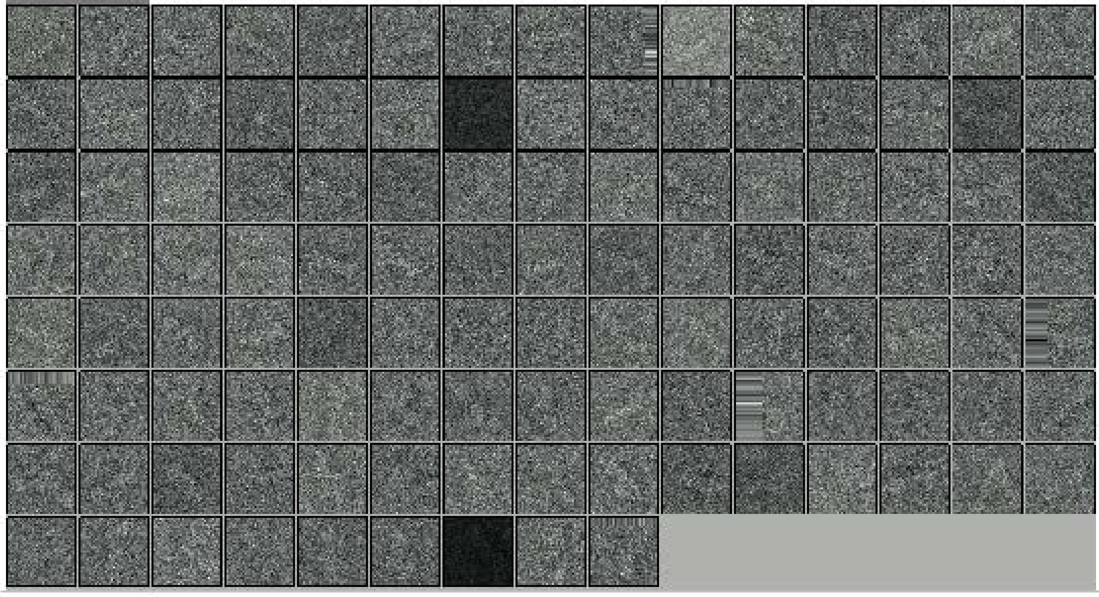

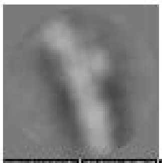

2D Class: 14

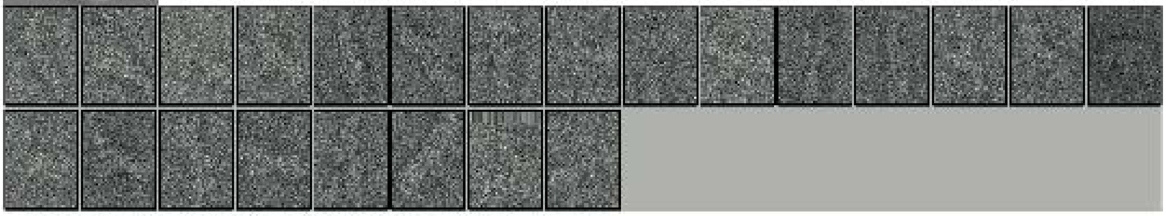

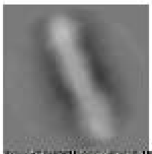

2D Class:15

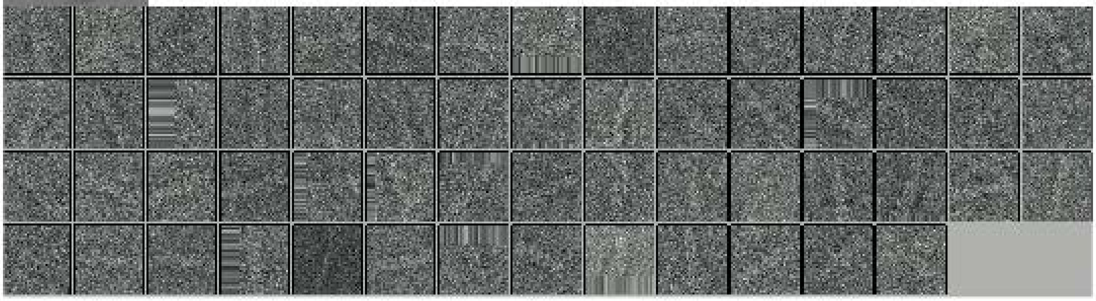

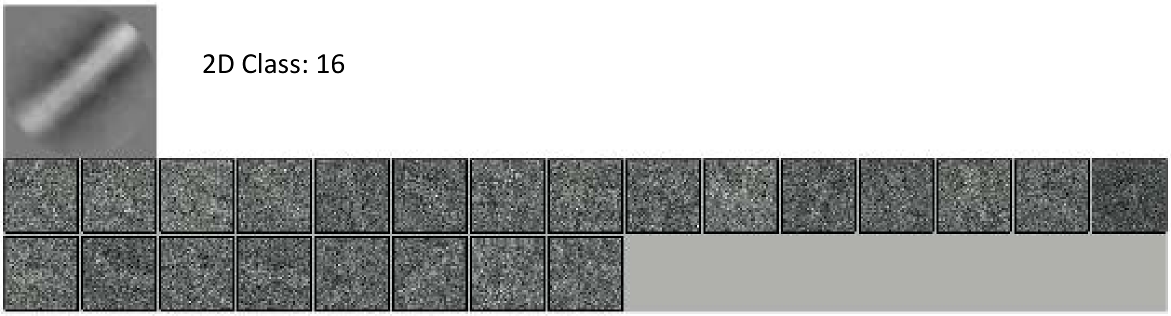

Supplementary Figure 6: c) Individual particles that went into the reference-free 2D class averages of the FLAG-FADD:Caspase-8 complex. Below each individual 2D class average are the particles for that class.

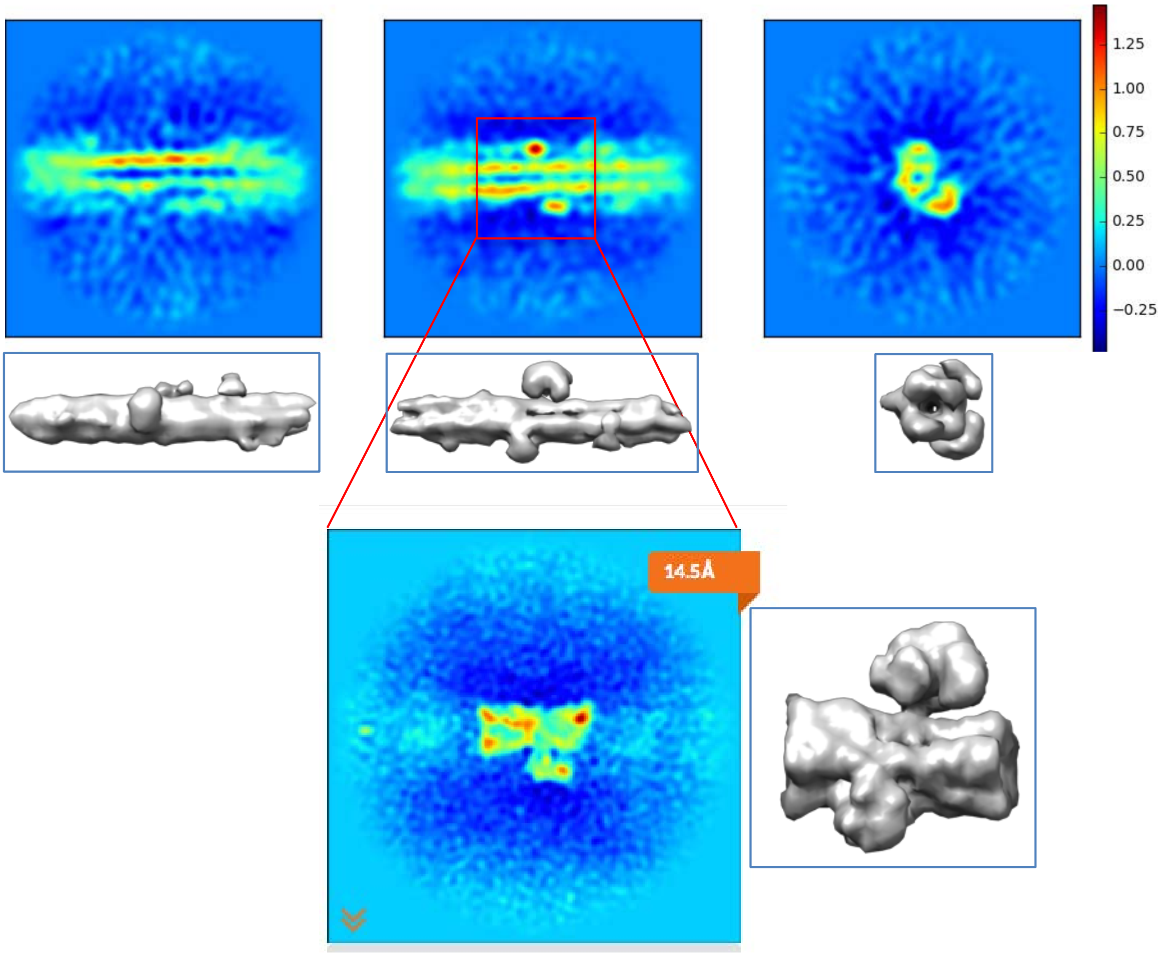

Supplementary Fig. 7: CryoSPARC 3D refinement of FLAG-FADD:Caspase-8 complex. Area highlighted with red box shows the area where a mask was applied to enable further refinement of the central region of the FLAG-FADD:Caspase-8 complex, including both tDEDs and catalytic domains shown in the call-out. 3D Volumes are shown in grey.

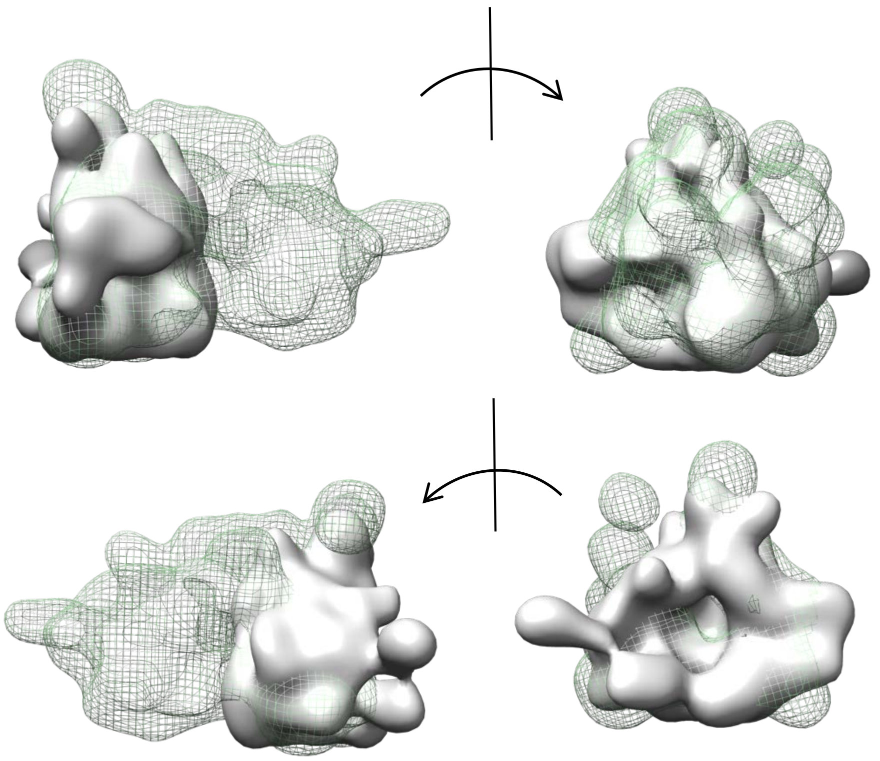

Supplementary Fig. 8: Comparison of refined initiating end (green mesh) and extending end (solid grey) of the FLAG-FADD:Caspase-8 complex.

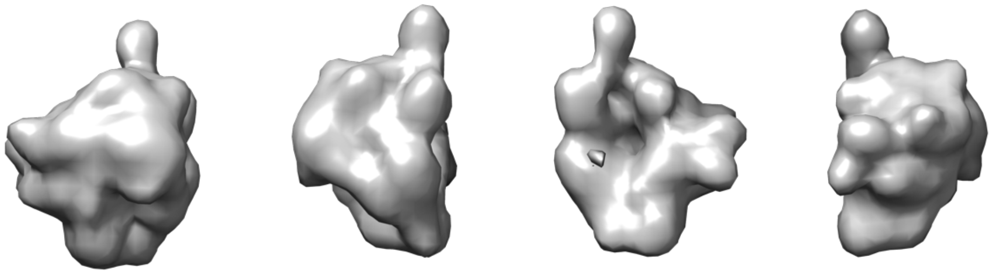

Supplementary Fig. 9: Refinement of extending end of the FLAG-FADD:Caspase-8 complex using a mask in CryoSPARC (~17Å).

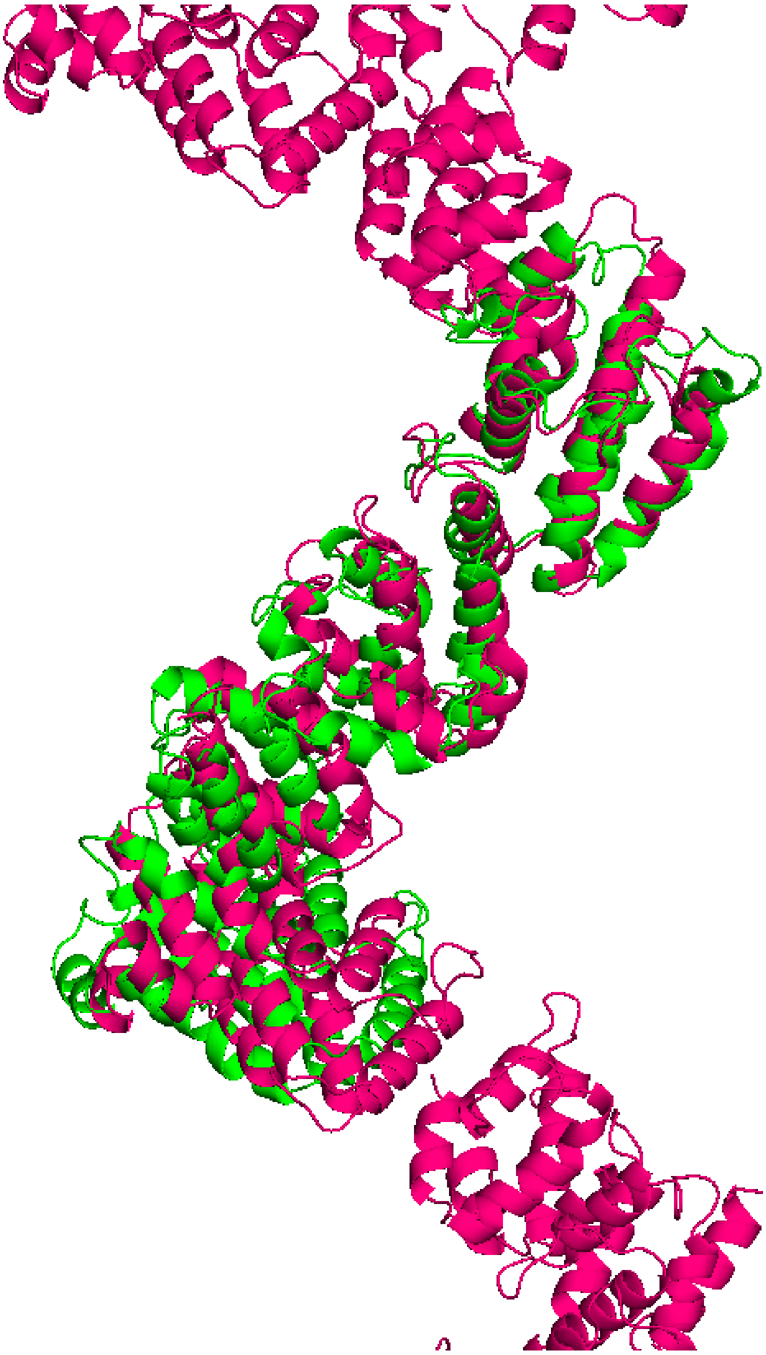

Supplementary Fig. 10: Comparison of helical Caspase-8 model based on F122/L123:Y8 canonical (Type I) tDED interactions proposed in Dickens *et al* [1; green]) and a single strand formed *via* the Type I interaction from the published tDED-only Cryo-EM structure 5L08, [2; red]).

a)

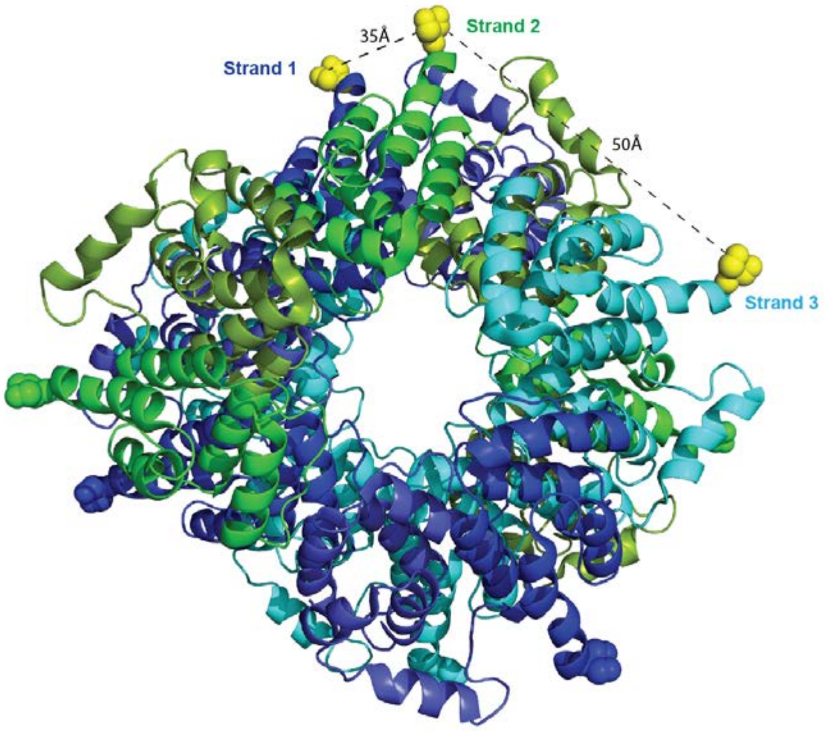

b)

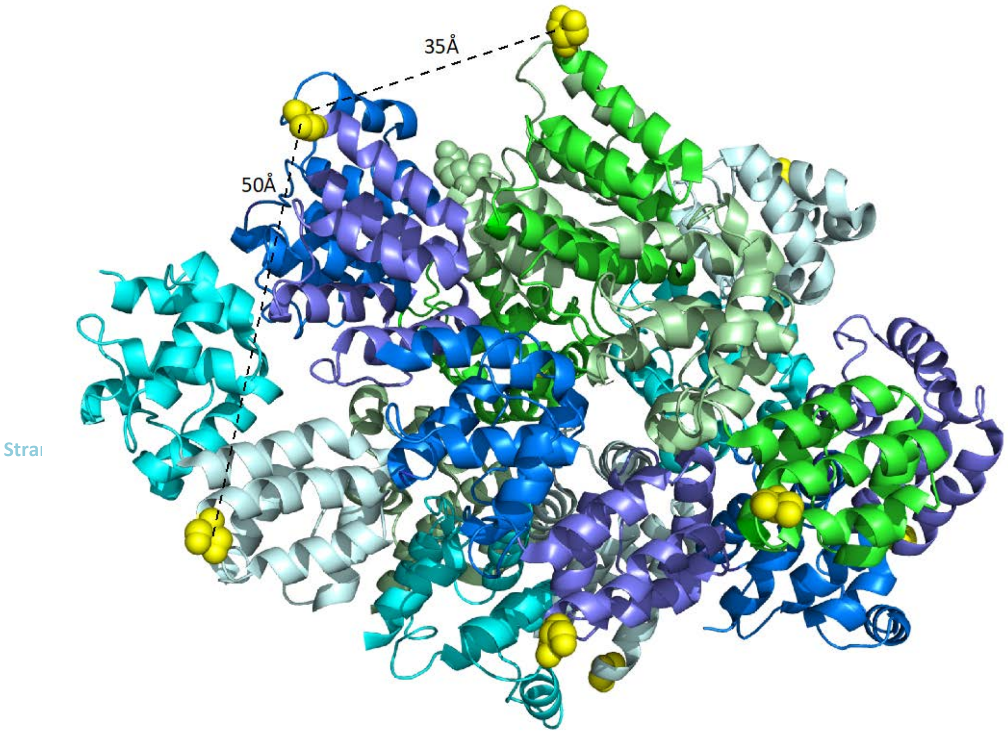

Supplementary Fig. 11: a) End- and **b)** side-views showing positions of last residue (S182) on each DED within the published tDED-only Cryo-EM structure 5L08; [2] shows relative position of the strands within the triple helix. tDEDs within Strand 2 (green) are rotated 20˚ relative to those in Strand 1 (blue), whereas Strand 3 (cyan) is rotated 70˚ relative to Strand 1. The distance between S182 residues on the different strands therefore differs, ~35Å between the aligned Strands 1 and 2, ~ 50Å between Strand 2 and the offset Strand 3.

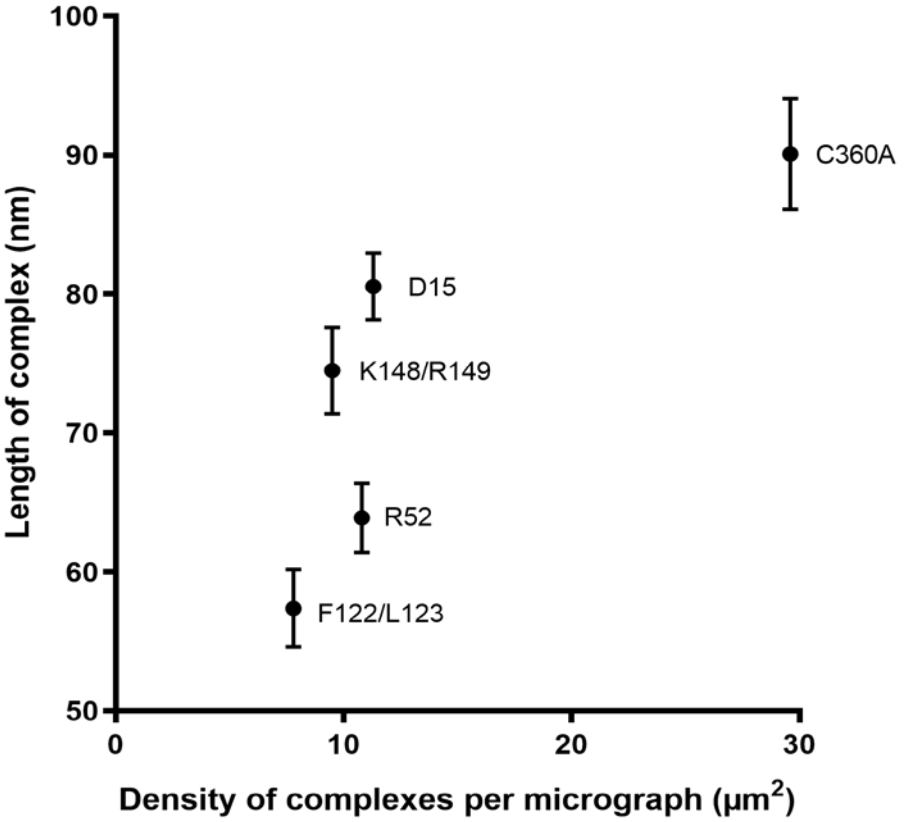

Supplementary Fig. 12: Effect of Caspase-8 Type I, II and III mutations on the average length of FLAG-FADD:Caspase-8 complexes formed, and in each case quantitation of the density of complexes visualized on the negative-stain micrographs. Quantitation of independent complexes of C360A (n=20), F122/L123 (n=18), D15 (n=26), K148/R149 (n=22), R52 (n=25), from multiple micrographs within one representative experiment from 3 biologically independent experiments (graph shows Mean ± SEM). Source data are provided as a Source Data file.

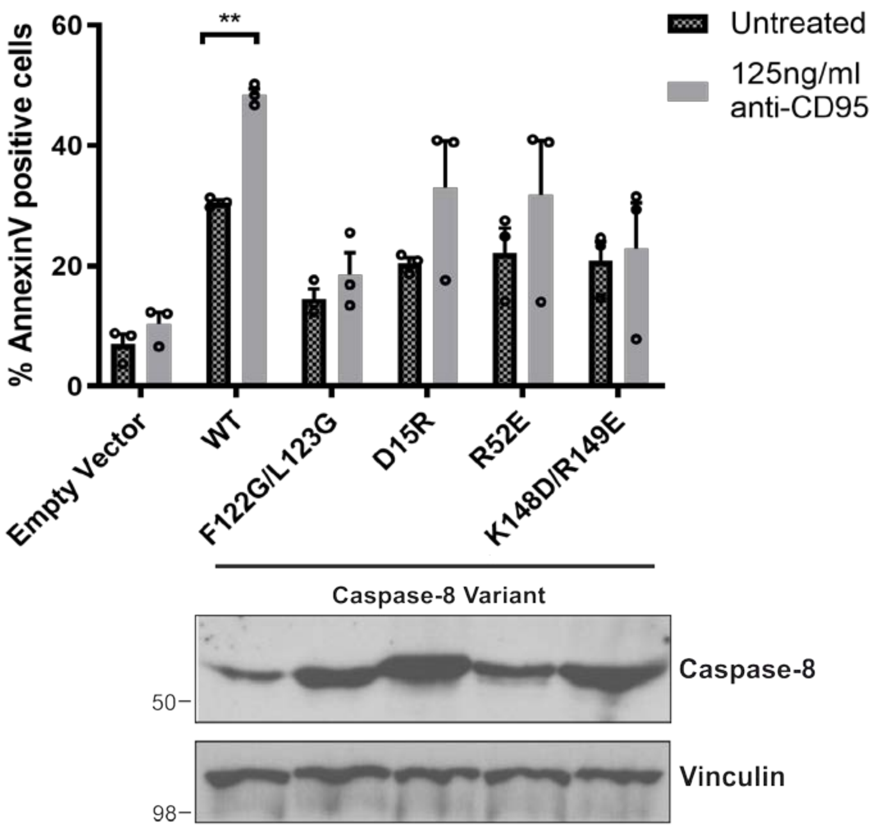

Supplementary Fig. 13: Effect of Type I, II and III mutations on the ability of re-expressed catalytically active Caspase-8 (WT) to rescue anti-CD95-induced cell death in Caspase-8-deficient Jurkat T cells. Cells were co-transfected with Caspase-8 variant together with GFP and apoptotic cell death determined by Annexin V-APC labelling of GFP-positive cells only (n=3 biologically independent experiments; Mean + SEM); background cell death induced by transient transfection alone is subtracted (~20%). Graphical representation of the FACS sequential gating strategy is shown in Supplementary Fig. 21; Annexin V-APC single labelling was used as a marker of early apoptotic cells while double-labelled Annexin V-APC/Draq 7 cells represented late apoptotic cells. Notably, a significant rescue of anti-CD95-induced cell death was only detected in cells re-expressing WT Caspase-8 (P=0.001 by paired t-test with 4df using a 1%FDR). Western blot, representative of 3 independent experiments, confirmed transient re-expression of Caspase-8 WT or Type I, II or III mutants in Caspase-8-deficient Jurkat T cells. Vinculin served as loading control. Source data are provided as a Source Data file.

a)

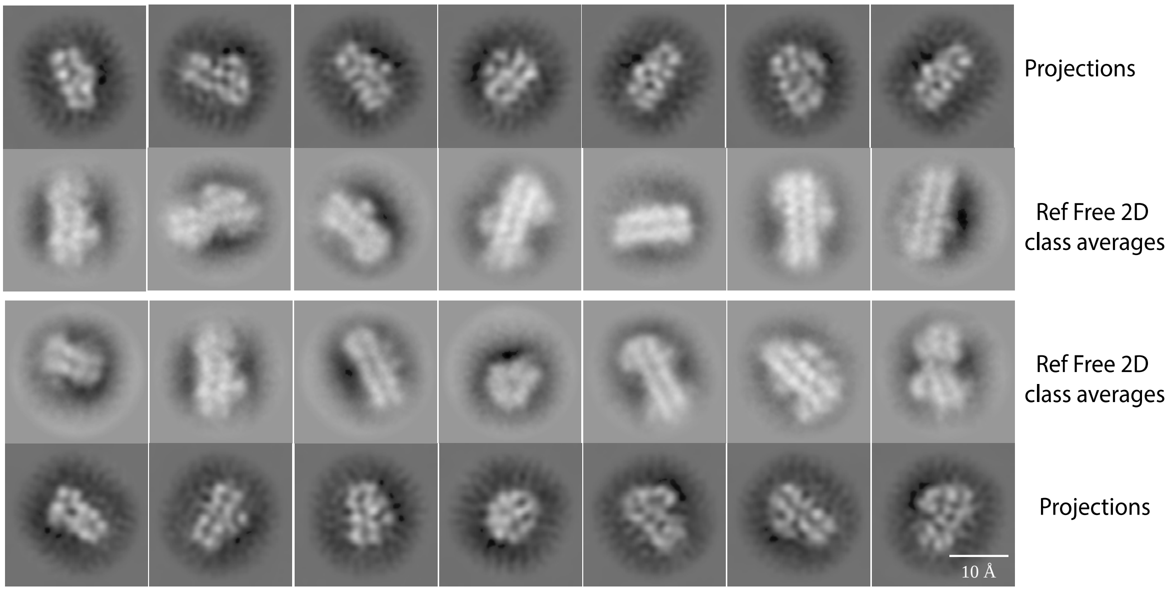

Supplementary Fig. 14: a) Comparison of negative-stain reference-free 2D class averages (central two rows), and 2-D re-projections of the 3D reconstruction in the corresponding orientation (top and bottom rows) of the FLAG-FLIP$_S$:FADD:Caspase-8 complex.

b)

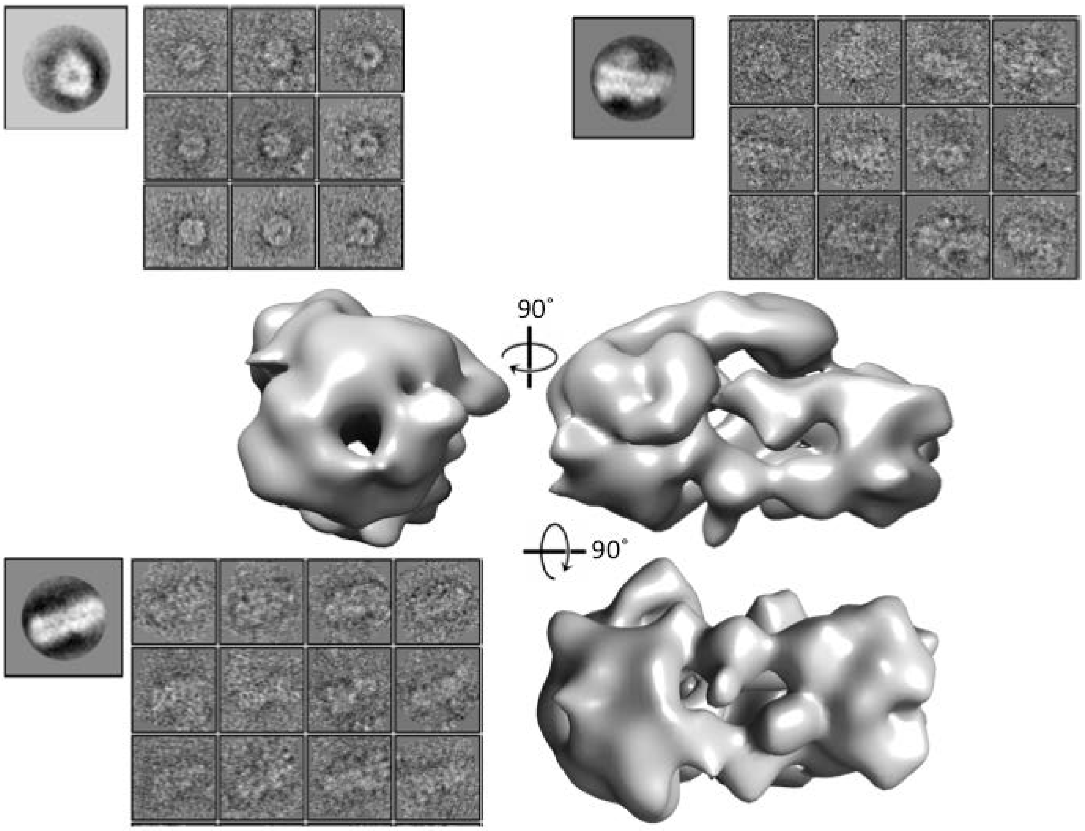

Supplementary Fig. 14: b) Comparison of representative negative-stain reference-free 2D class averages and 3D reconstruction with the individual particles in each class correctly orientated with the class average, highlighting key features in the FLAG-FLIPS:FADD:Caspase-8 complex.

c)

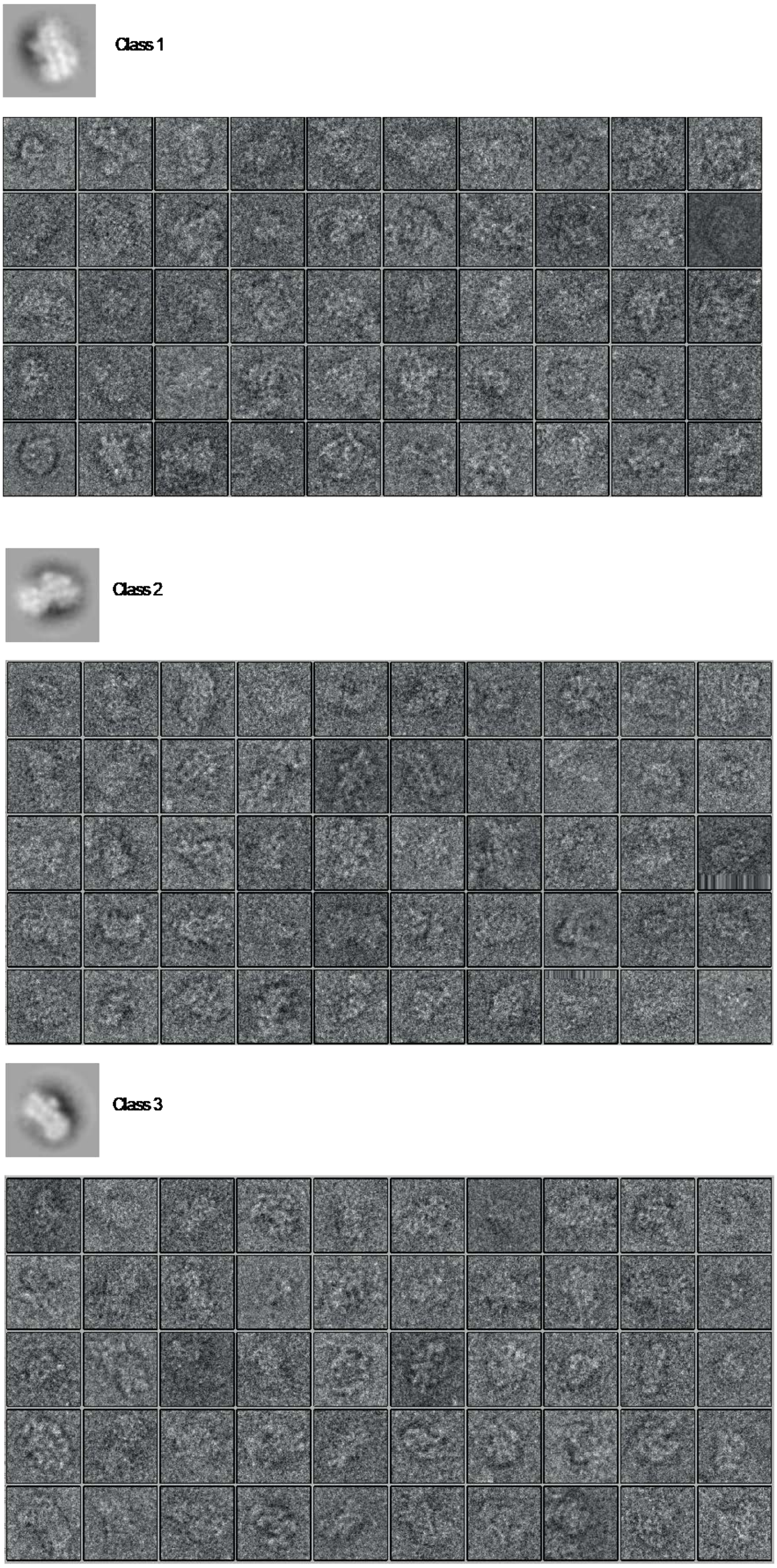

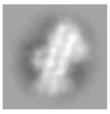

Class 4

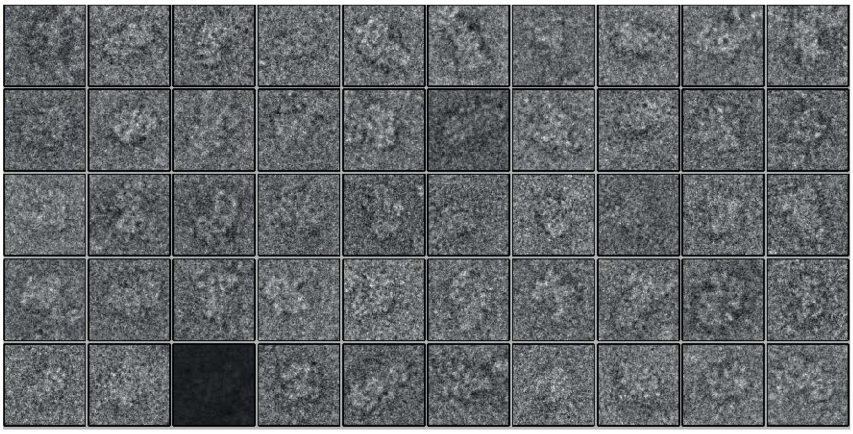

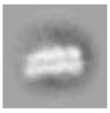

Class 5

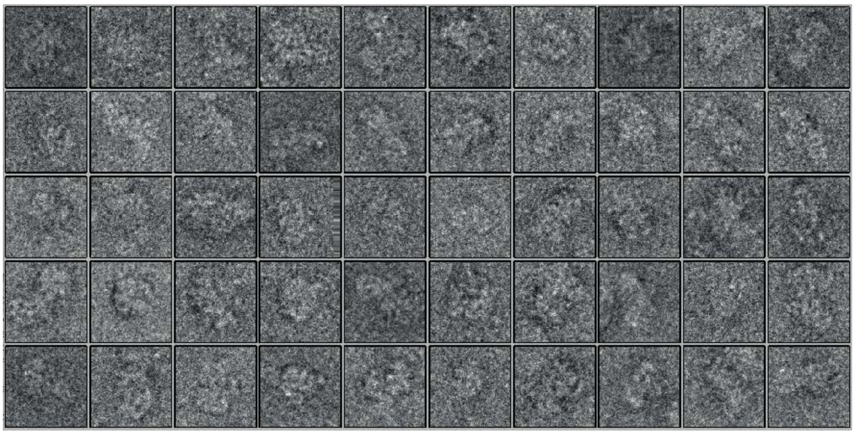

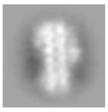

Class 6

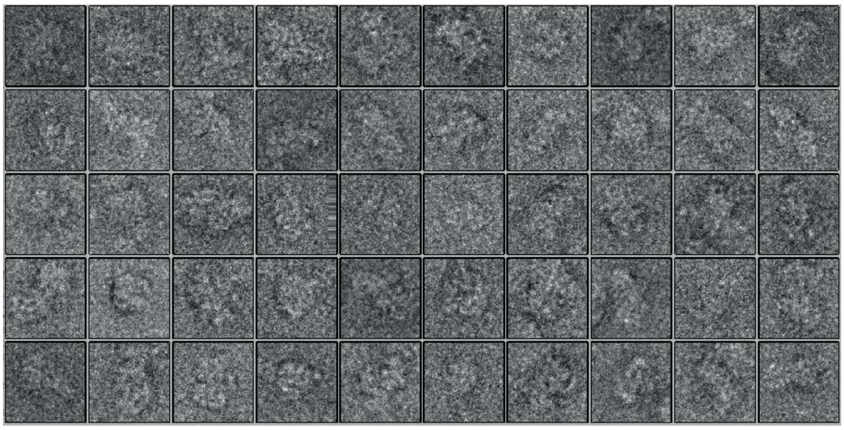

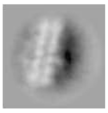

Class 7

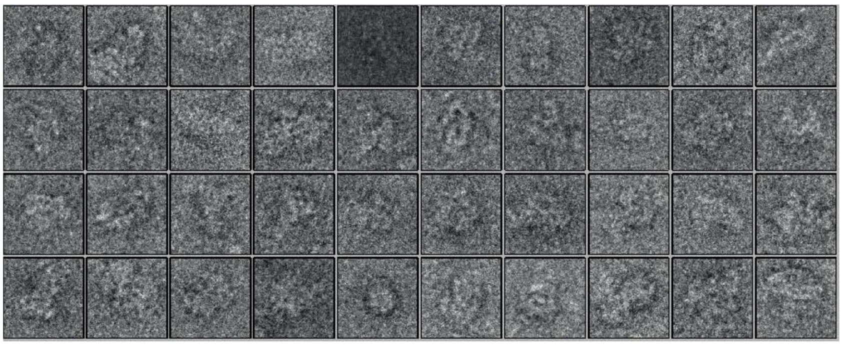

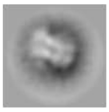

Class 8

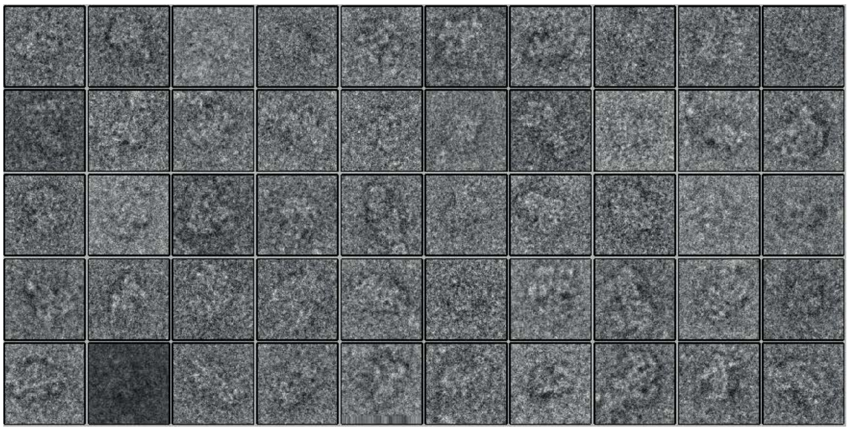

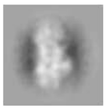

Class 9

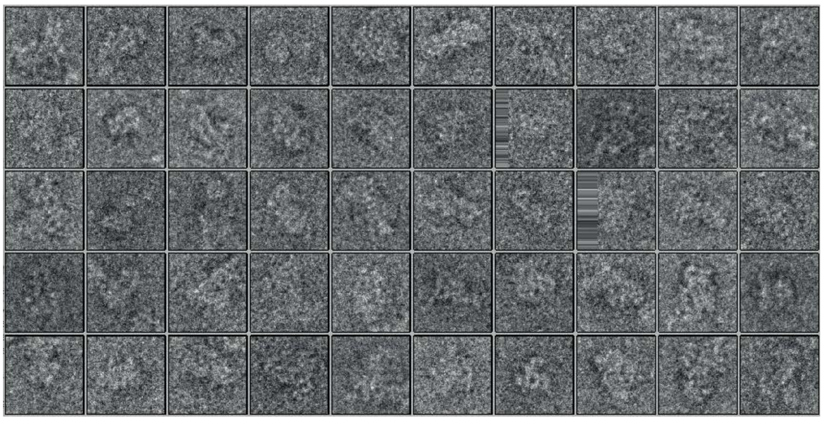

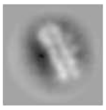

Class 10

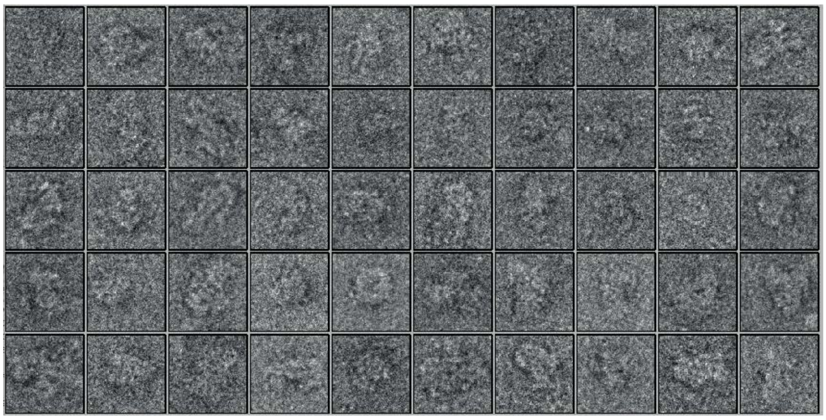

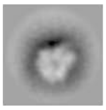

Class 11

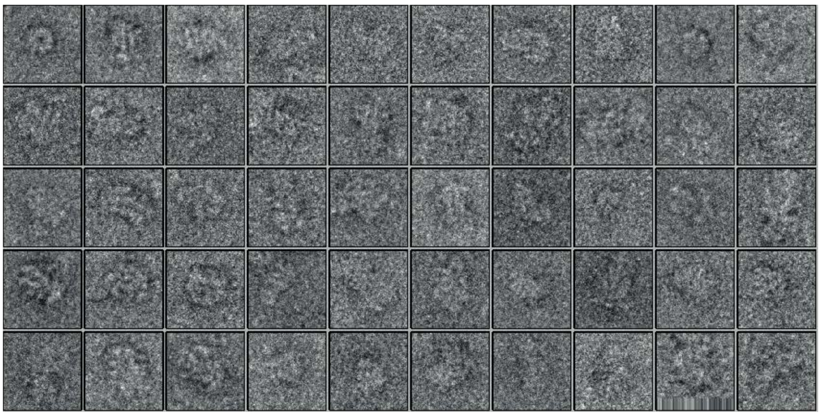

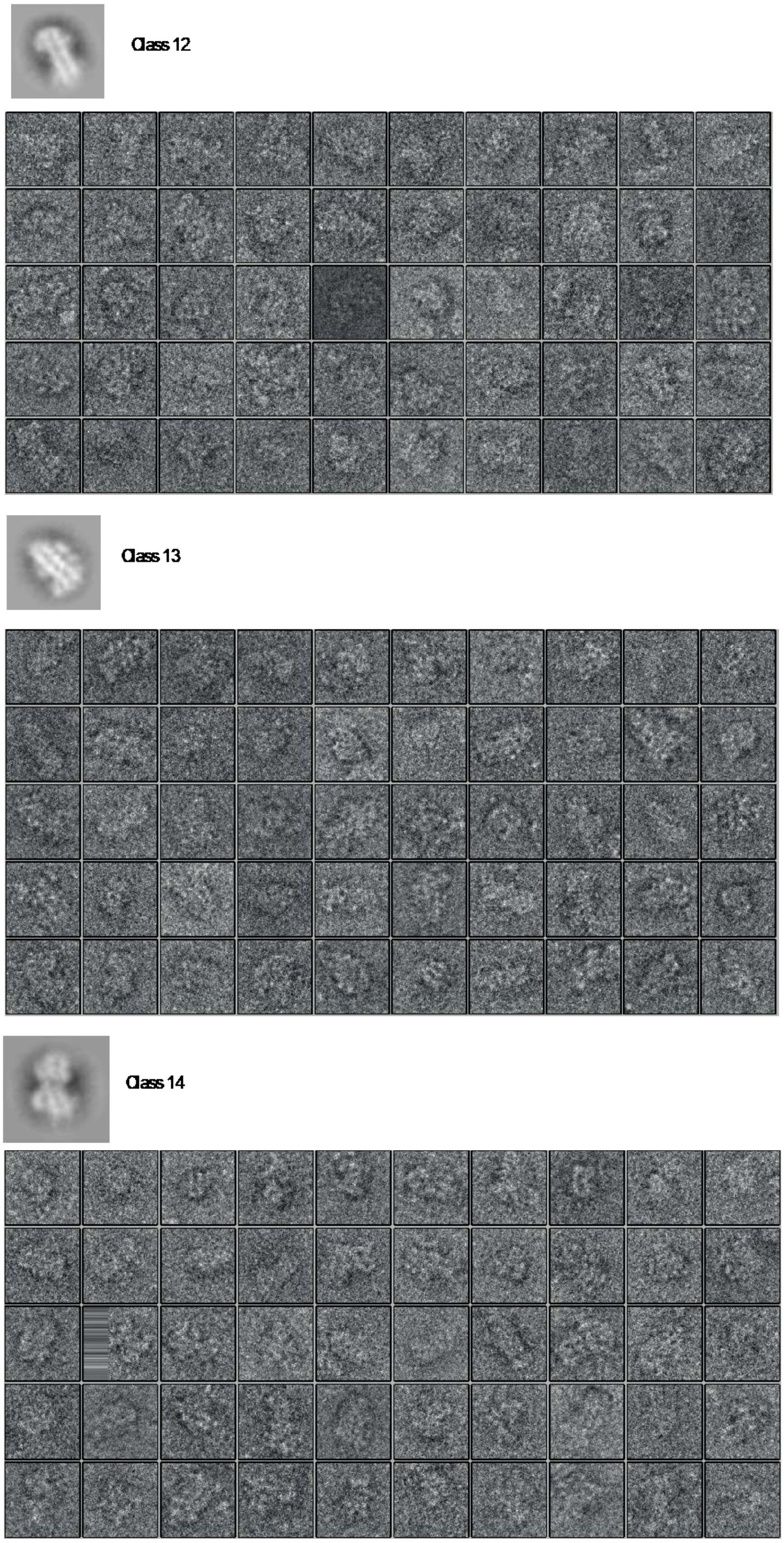

Supplementary Figure 14: c) Individual particles that went into each of the negative-stain reference-free 2D class averages of the FLAG-FLIP$_S$:FADD:Caspase-8 complex. Below each individual 2D class average are the 50 representative particles for that class.

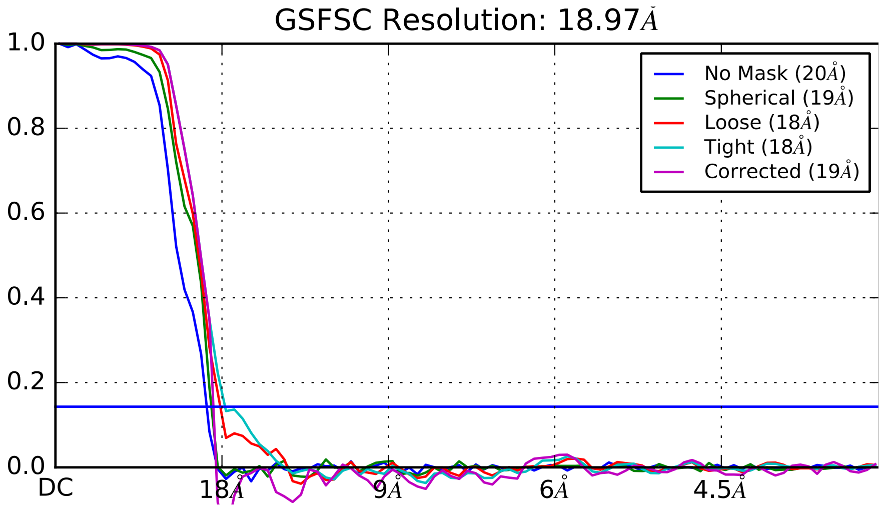

Supplementary Fig. 15: FSC Curve for Negative-stain dataset of FLAG-FADD:Caspase-8 complexes analysed using CryoSPARC v2.1.

The default settings were used for the HADDOCK webserver (v2.1) which are listed below:
Number of structures generated: Semi-flexible refinement:200
Ambiguous interaction restraints (AIRs) treatment: By default, 50% of the AIRs will be randomly deleted for each docking trial
Restraints validation: All restraints (distances, hbonds, dihedral angle, RDCs and diffusion anisotropy are submitted to a strict validation by the server. They should comply to CNS syntax.
Flexibility treatment: Semi-flexible residues are automatically defined from an analysis of intermolecular contacts(<5.0A)
Protonation state of histidines: The protonation state of histidine is automatically defined by querying the Whatif web server
Co-factors and ligands: Missing parameter and topology files for co-factors and small ligands are automatically obtained from the PRODRG web server

Clustering parameters: FCC cut-off = 0.75; minimum number of members in a cluster: 4
Final scoring: The reported scores and energies are averages calculated over the top four members of a cluster. The HADDOCK score is defined as: HADDOCK-score = $1.0*E_{VDW}+0.2*E_{ELEC}+1.0*E_{Desolvation}$

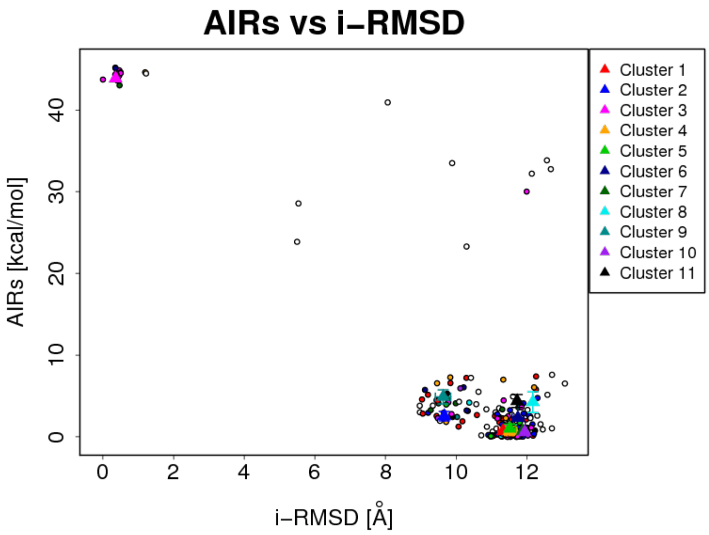

For the docking - Caspase-8 tDEDs was molecule A, residues F122/L123 were active. FLIP tDEDs (threaded using Phyre based on vFLIP) was molecule B, residues I6,H7,L41 were passive.

The clusters grouped into three positions on the AIRs vs iRMSD graph; Cluster 1, 2, and 3 are representative of the different potential interactions modelled.

	**Cluster 1**	**Cluster 2**	**Cluster 3**
HADDOCK score	-87.1 +/- 4.6	-65.9 +/- 3.2	-91.4 +/- 2.3
Cluster size	42	29	18
RMSD from overall lowest-energy structure	15.2 +/- 0.4	11.4 +/- 0.3	-0.3 +/- 0.2
Van der Waals energy	-21.7 +/- 8.8	-9.5 +/- 3.9	-24.0 +/- 3.1
Electrostatic energy	-173.5 +/- 23.6	-125.0 +/- 45.7	-230.9 +/- 9.9
Desolvation energy	-30.7 +/- 4.7	-31.6 +/- 10	-25.6 +/- 3.6
Restraints violation energy	0.7 +/- 0.84	2.5 +/- 1.15	43.9 +/- 0.56
Buried surface area	874.0 +/- 96.3	532.8 +/- 34.6	1009.8 +/- 25.4
	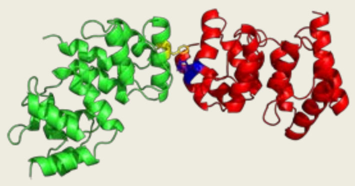	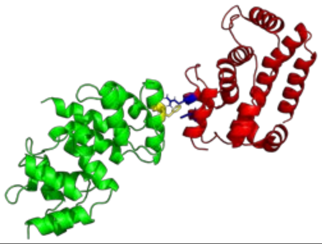	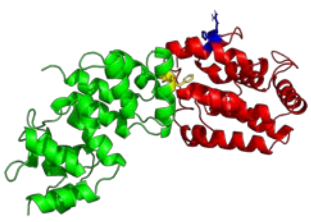

Supplementary Fig. 16: Results of the HADDOCK docking of the interface between Caspase-8:c-FLIP$_{S}$. Cluster 1 was selected representative of the majority of clusters; it also had the largest cluster size.

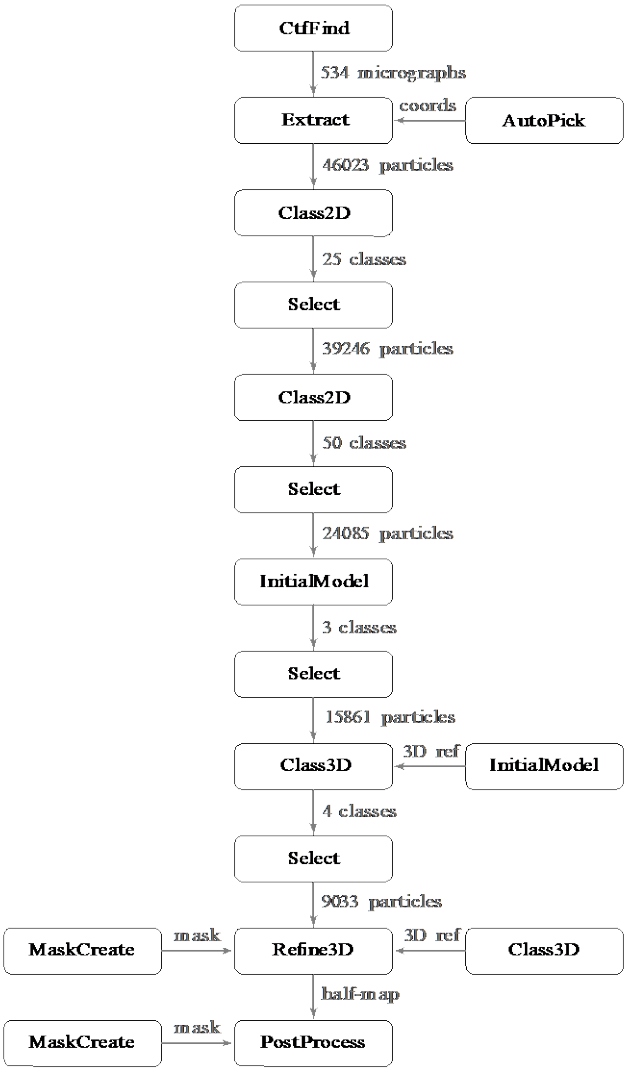

Supplementary Fig. 17: Flowchart of single particle analysis of FADD:Caspase-8:FLAG-c-$FLIP_S$ complex dataset collected by Cryo-EM in RELION 3.

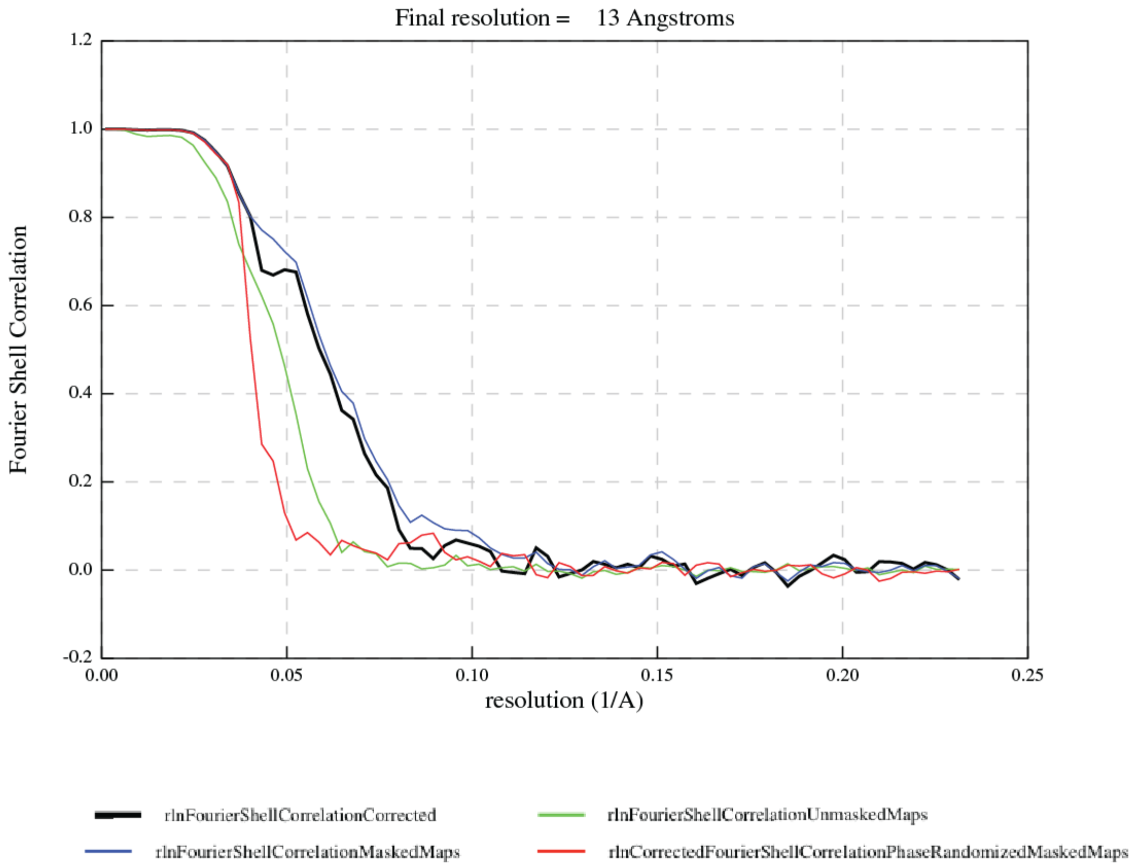

Supplementary Fig. 18: FSC Curve for single particle analysis of FADD:Caspase-8:FLAG-c-$FLIP_S$ complex dataset collected by Cryo-EM and processed in RELION 3.

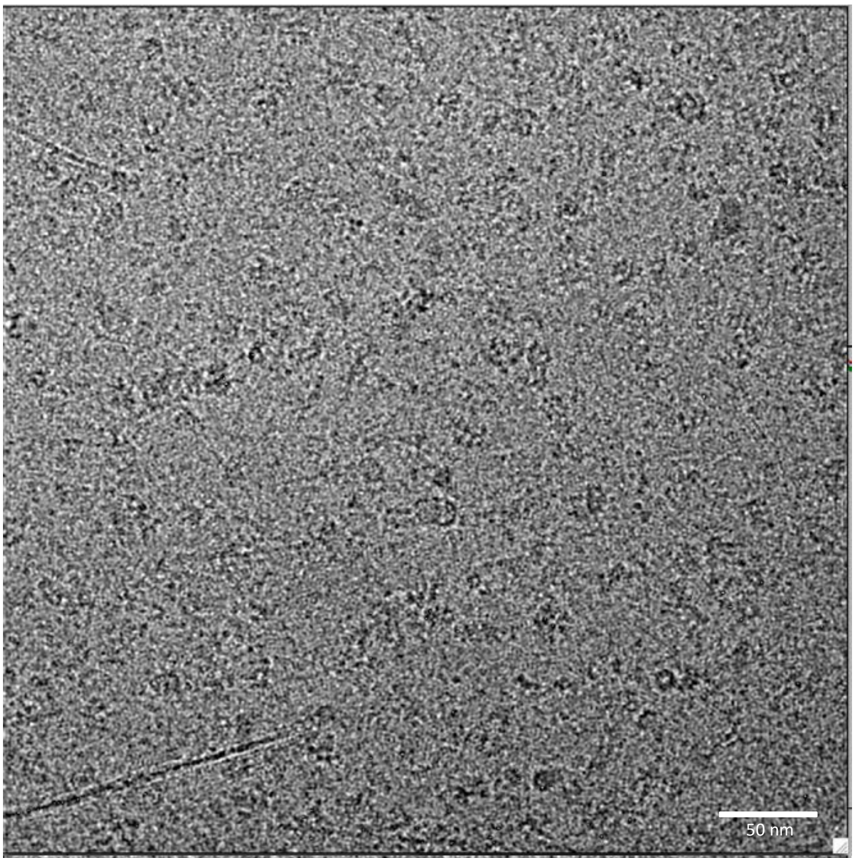
50 nm

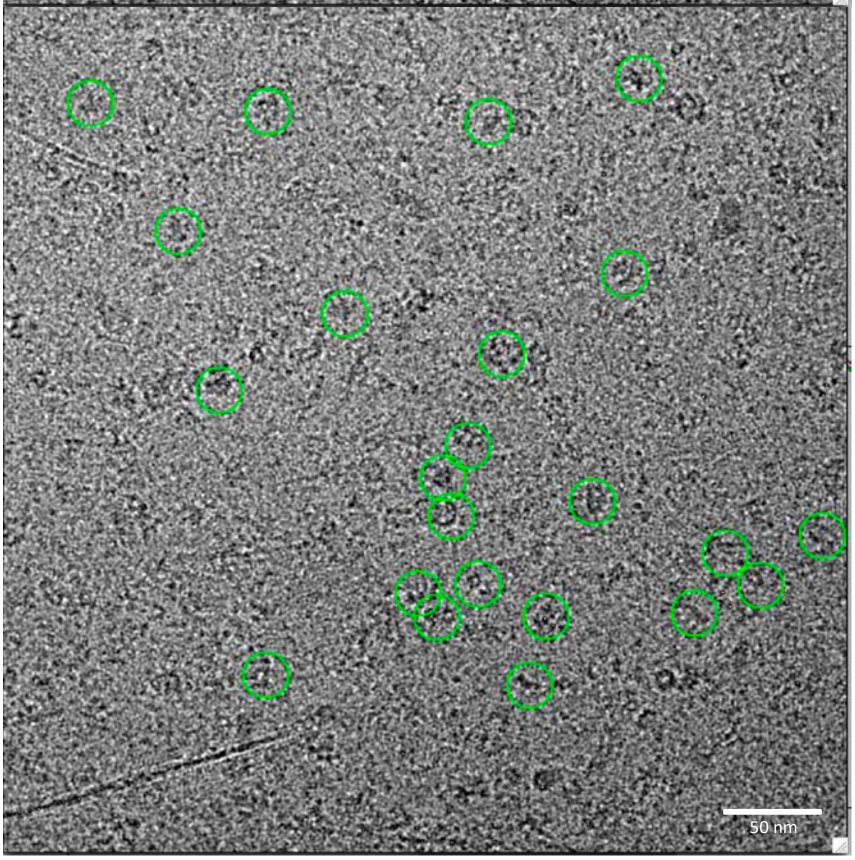
50 nm

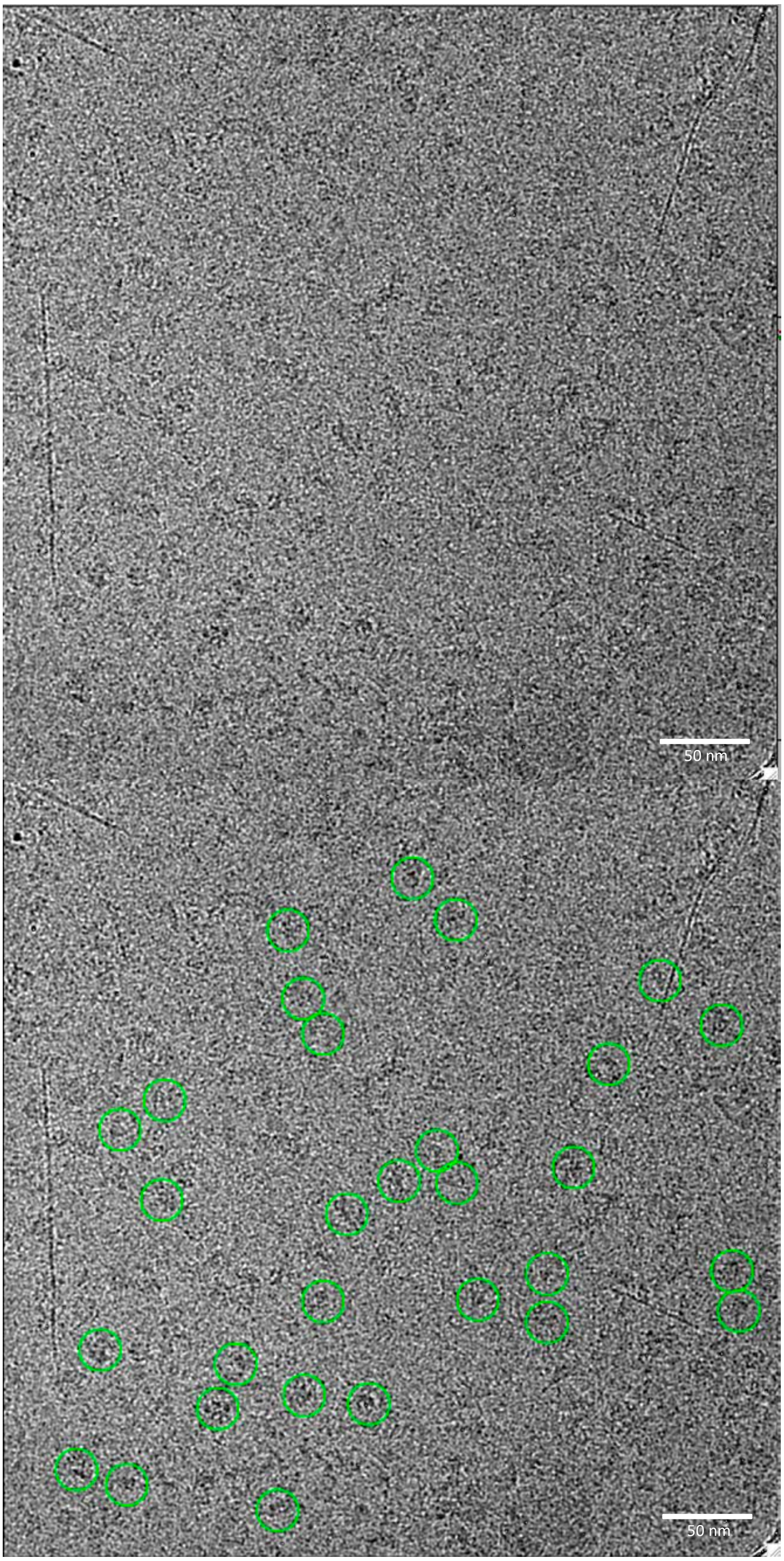
50 nm
50 nm

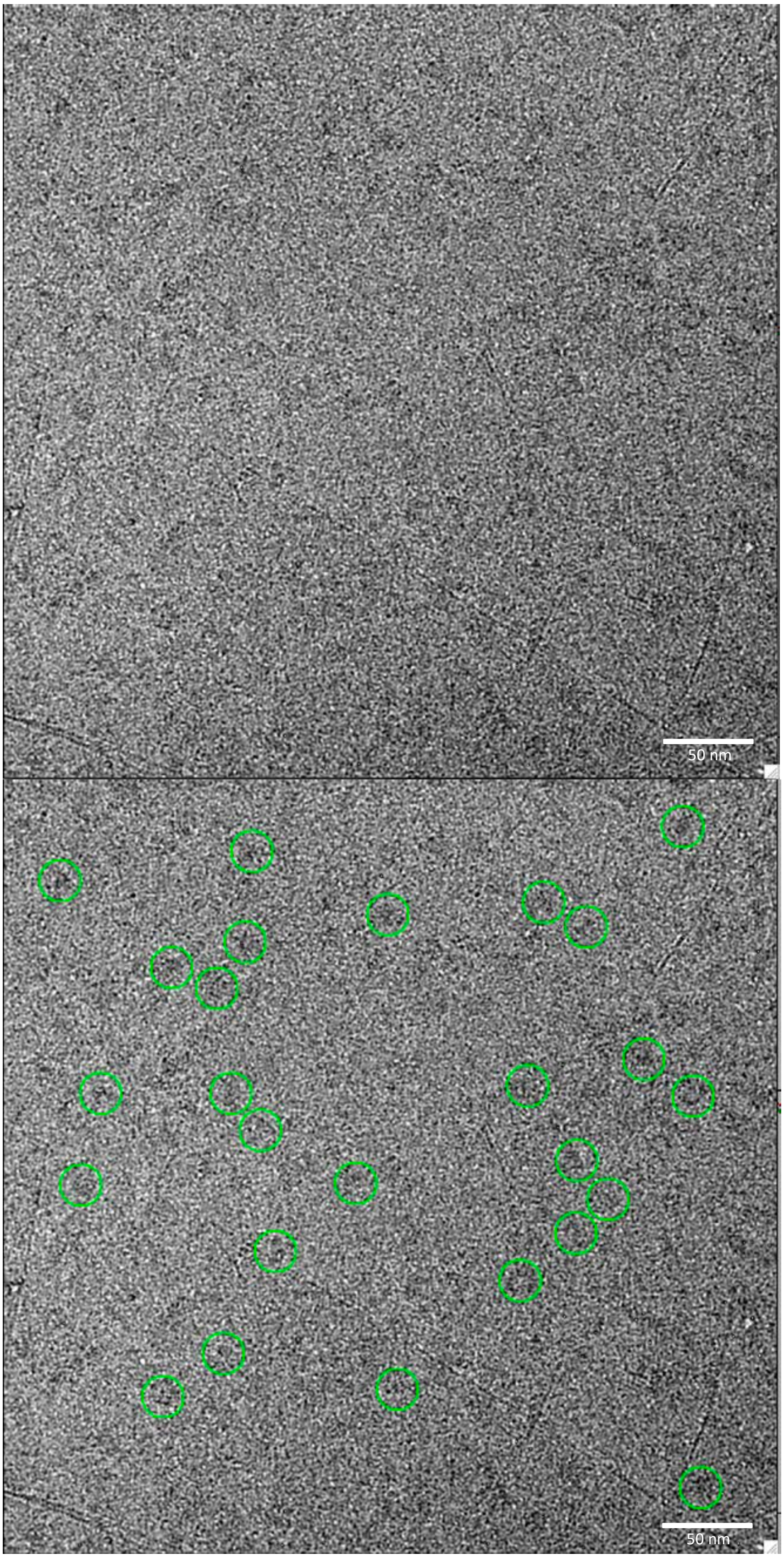

Supplementary Fig. 19: Representative micrographs of FADD:Caspase-8:FLAG-c-FLIP$_S$ complexes collected by Cryo-EM. In each case, top image shows unpicked micrograph, bottom image particles picked (green circles) from micrograph by CrYOLO. A single dataset was collected by CryoEM for this complex. These complexes are representative of those on

the 534 micrographs collected. In addition, n=3 biologically independent experiments were used to optimise grid preparation, and the complexes on those grids were representative of those in the full dataset.

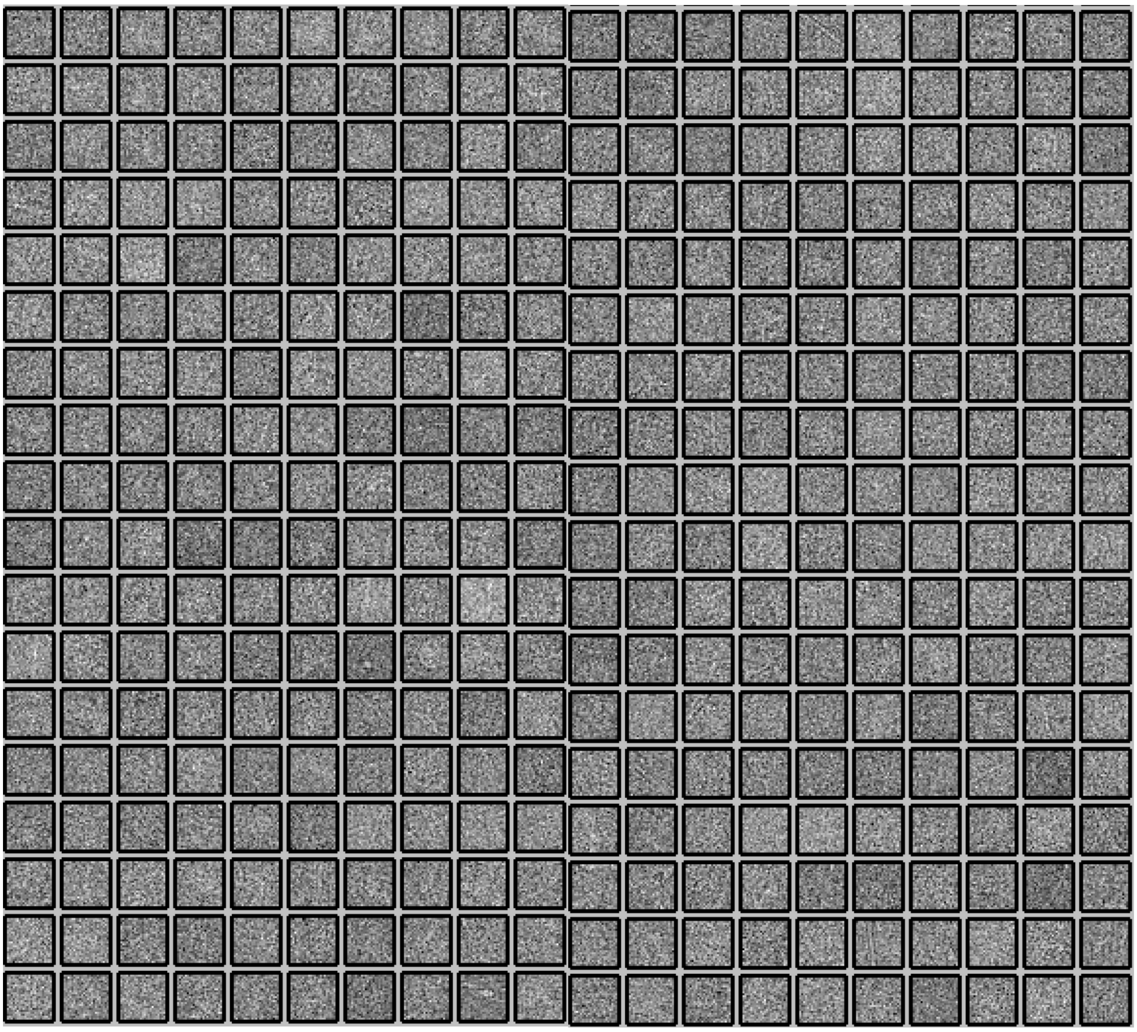

Supplementary Fig. 20: Representative single particles selected from micrographs of the FADD:Caspase-8:FLAG-c-FLIP$_S$ complex dataset collected by Cryo-EM.

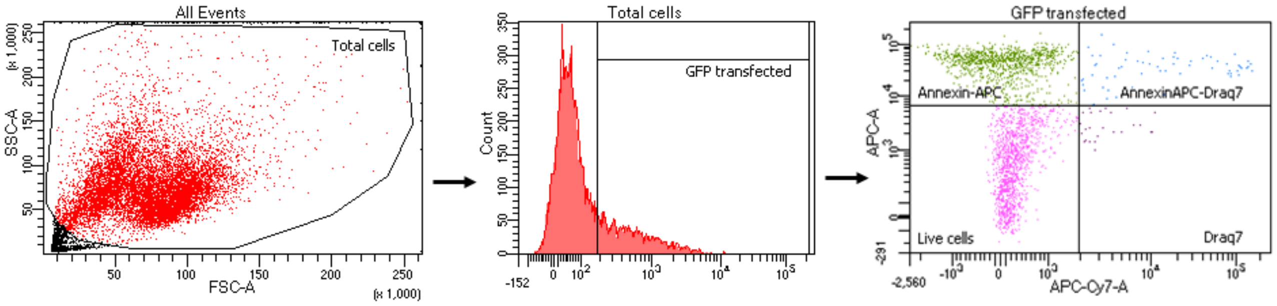

Tube: Gating Strategy

Population	#Events	%Parent
All Events	11,376	####
Total cells	10,163	89.3
GFP transfected	2,484	24.4
Annexin-APC	1,072	43.2
AnnexinAPC-Draq7	64	2.6
Live cells	1,322	53.2
Draq7	26	1.0

Supplementary Fig. 21: Graphical representation of FACS sequential gating strategy for analysis of apoptotic cell death using Annexin V-APC labelling. By using GFP as a transfection marker, cell death in transfected cells was specifically quantified using Annexin V-APC alone or Draq 7 co-labelling and gating of GFP-transfected cells only. Annexin V-APC labelling alone was used as a marker of early apoptotic cells, while double-labelled Annexin V-APC-Draq 7 cells represent late apoptotic cells. Double-negative cells represent viable/live cells.

	FLAG-FADD:Caspase-8 (negative stain)	FLAG-FLIP:Caspase-8:FADD (negative stain)	FLAG-FLIP:Caspase-8:FADD (CryoEM)
Number of initial particles in dataset	3200	7493	46023
Number of particles in final reconstruction	1002	2772	9033
Resolution of final reconstruction	Whole particle 22Å<br>Masked central region 15Å	19Å	13Å
**Data Collection Parameters**			
Number of Micrographs	620	477	534
Microscope Voltage	200Kv	200Kv	300Kv
Nominal magnification	x57,000	x57,000	X 64,000
Pixel size	1.8 Å/pix	1.8 Å/pix	1.08 Å/pix
Defocus range	-1 to -3	-1 to -3	-1.5 to - 3.5
Total electron dose	35 e/Å^2	35 e/Å^2	35 e/Å^2

Supplementary Table 1: Summary of Caspase-8 complex EM structure studies.

Primer Name	Primer Sequence
CRISPR gRNA Casp8-1:	GCCTGGACTACATTCCGCAA
CRISPR gRNA Casp8-2	GCTCTTCCGAATTAATAGAC

Supplementary Table 2: CRISPR gRNA sequences used in this study.

References

1. Dickens, L.S. et al. A death effector domain chain DISC model reveals a crucial role for caspase-8 chain assembly in mediating apoptotic cell death. *Mol Cell* **47**, 291-305 (2012).

2. Fu, T.M. et al. Cryo-EM Structure of Caspase-8 Tandem DED Filament Reveals Assembly and Regulation Mechanisms of the Death-Inducing Signaling Complex. *Mol Cell* **64**, 236-250 (2016).
