# Supplementary material for: Cryo-EM structural analysis of FADD:Caspase-8 complexes defines the catalytic dimer architecture for co-ordinated control of cell fate
Source: Nat Commun. 2021 Feb 5;12:819. doi: 10.1038/s41467-020-20806-9 (PMC7864959; doi:10.1038/s41467-020-20806-9)
Supplement: Supplementary file 2 — Description of Additional Supplementary Files [file 41467_2020_20806_MOESM2_ESM.docx]

**Description of Additional Supplementary Files**

File Name: Supplementary Movie 1: Model of FADD-nucleated full-length Caspase-8 showing tDEDs and catalytic domain assembly.

Description: Each strand of the helix is colored; blue and green strands contain aligned DEDs, while in the cyan strand the DEDs are offset. Caspase-8 catalytic domain subunit colors reflect the DED strand from which they originate (blue/green).

File Name: Supplementary Movie 2: Model of full-length Caspase-8 in complex with c-FLIP_S_ showing inhibition of both tDED and catalytic domain assembly.

Description: Each strand of the tDED helix is colored; tDEDs in blue and green strands are aligned, while tDEDs in cyan strand are offset. In the presence of c-FLIP_S_ (red), catalytic domains originating from aligned strands (blue/green) do not assemble into a canonical catalytic dimer.
