# Supplementary material for: Cryo-EM structural analysis of FADD:Caspase-8 complexes defines the catalytic dimer architecture for co-ordinated control of cell fate
Source: Nat Commun. 2021 Feb 5;12:819. doi: 10.1038/s41467-020-20806-9 (PMC7864959; doi:10.1038/s41467-020-20806-9)
Supplement: Supplementary file 5 — Reporting Summary [file 41467_2020_20806_MOESM5_ESM.pdf]

nature research

Corresponding author(s): Marion MacFarlane & Joanna Fox

Last updated by author(s): Dec 1, 2020

Reporting Summary

Nature Research wishes to improve the reproducibility of the work that we publish. This form provides structure for consistency and transparency in reporting. For further information on Nature Research policies, see Authors & Referees and the Editorial Policy Checklist .

Statistics

For all statistical analyses, confirm that the following items are present in the figure legend, table legend, main text, or Methods section.

n/a	Confirmed	
☐	☒	The exact sample size (*n*) for each experimental group/condition, given as a discrete number and unit of measurement
☐	☒	A statement on whether measurements were taken from distinct samples or whether the same sample was measured repeatedly
☐	☒	The statistical test(s) used AND whether they are one- or two-sided<br>*Only common tests should be described solely by name; describe more complex techniques in the Methods section.*
☐	☒	A description of all covariates tested
☐	☒	A description of any assumptions or corrections, such as tests of normality and adjustment for multiple comparisons
☐	☒	A full description of the statistical parameters including central tendency (e.g. means) or other basic estimates (e.g. regression coefficient) AND variation (e.g. standard deviation) or associated estimates of uncertainty (e.g. confidence intervals)
☐	☒	For null hypothesis testing, the test statistic (e.g. *F*, *t*, *r*) with confidence intervals, effect sizes, degrees of freedom and *P* value noted<br>*Give P values as exact values whenever suitable.*
☒	☐	For Bayesian analysis, information on the choice of priors and Markov chain Monte Carlo settings
☒	☐	For hierarchical and complex designs, identification of the appropriate level for tests and full reporting of outcomes
☒	☐	Estimates of effect sizes (e.g. Cohen's *d*, Pearson's *r*), indicating how they were calculated

Our web collection on statistics for biologists contains articles on many of the points above.

Software and code

Policy information about availability of computer code

Data collection	FEI EPU 1.11
Data analysis	GraphPad Prism 7.0 and 8.0<br>Relion2.1<br>Relion3.0<br>EMAN2<br>CryoSPARC v1<br>Motioncorr2<br>CrYOLO v1.5<br>UCSF Chimera v1.13.1<br>HADDOCK 2.0<br>Waters ProteinLynx Global SERVER (PLGS 3.0) using the UniProt Human database (UniProtKB release 2014_11, 20,265 entries)

For manuscripts utilizing custom algorithms or software that are central to the research but not yet described in published literature, software must be made available to editors/reviewers. We strongly encourage code deposition in a community repository (e.g. GitHub). See the Nature Research guidelines for submitting code & software for further information.

Data

Policy information about availability of data

All manuscripts must include a data availability statement. This statement should provide the following information, where applicable:

- Accession codes, unique identifiers, or web links for publicly available datasets
- A list of figures that have associated raw data
- A description of any restrictions on data availability

Negative-stain and Cryo-EM density maps have been deposited in the Electron Microscopy Data Bank under accession codes: EMD-1198 (Ternary Complex of full-length FLAG-FADD:Caspase-8), EMD-11939 (Central region of Caspase-8:FADD ternary complex) and EMD-11940 (Ternary Complex of full-length Caspase-8 with FADD and FLAG-FLIPs), EMD-11941 (Cryo-EM analysis of ternary complex of full-length Caspase-8 with FADD and FLIPs).
The mass spectrometry proteomics data have been deposited to the ProteomeXchange Consortium via the PRIDE partner repository with the dataset identifier PXD022408.
Source data for Figs. 1b & 1d, Fig. 2c, Fig. 4a & 4c, Fig. 5a, Fig. 6b, and Supplementary Figs. 1, 2a, 12 and 13 are provided with this paper as a Source Data File. All other data will be made available upon reasonable request.

Field-specific reporting

Please select the one below that is the best fit for your research. If you are not sure, read the appropriate sections before making your selection.

☒ Life sciences ☐ Behavioural & social sciences ☐ Ecological, evolutionary & environmental sciences

For a reference copy of the document with all sections, see nature.com/documents/nr-reporting-summary-flat.pdf

Life sciences study design

All studies must disclose on these points even when the disclosure is negative.

Sample size	No statistical method was used to predetermine sample size. The sample size was determined from preliminary experiments and was based on the reproducibility between independent experiments. The number of independent experiments and replicates (n) is indicated in the Figure Legends.
Data exclusions	No samples were excluded from analysis
Replication	Each experiment was successfully repeated at least three times. Western blots are representative blots from the 3 replicates. Each TEM experiment was repeated at least 3 times. Data from a single representative experiment is presented in the manuscript. Negative stain - 620 micrographs collected from which 3200 particles were analyzed. Cryo-EM - 534 movies collected (39 frames per movie), from which 12918 particles were analysed.
Randomization	As our data do not include clinical trials or related experiments, randomization was not applied.<br>For experiments involving cells, all conditions were randomly plated at the same time and all experimental procedures were strictly similar between the different conditions.
Blinding	No blinding method was possible as only one experimenter was performing the analyses.

Reporting for specific materials, systems and methods

We require information from authors about some types of materials, experimental systems and methods used in many studies. Here, indicate whether each material, system or method listed is relevant to your study. If you are not sure if a list item applies to your research, read the appropriate section before selecting a response.

Materials & experimental systems

n/a	Involved in the study	
☐	☒	Antibodies
☐	☒	Eukaryotic cell lines
☒	☐	Palaeontology
☒	☐	Animals and other organisms
☒	☐	Human research participants
☒	☐	Clinical data

Methods

n/a	Involved in the study	
☒	☐	ChIP-seq
☐	☒	Flow cytometry
☒	☐	MRI-based neuroimaging

Antibodies

Antibodies used	The following antibodies were used:<br>FADD mouse monoclonal antibody (mAb), clone 1/FADD, Cat no. 610400 from BD Transduction Laboratories;

Myc mouse mAb, clone 9B11, Cat no. 2276 from Cell Signalling;
Caspase-8 antibody p18 specific mouse mAb, clone C15, Cat no. AG-20B-0057-C050 from Adipogen;
Caspase-8, N-term specific mouse mAb, clone E6, Cat no. 04-574 from Millipore;
c-FLIP mouse mAb (NF-6), Cat no. AG-20B-0056-C050 from Adipogen;
Caspase-10 mouse mAb, clone 4C1, Cat no. M059-3 from MBL;
Vinculin mouse mAb, clone VIN-54, Cat no. ab130007 from Abcam.
Anti-FLAG M2 mouse mAb, Cat no. F3165 from Sigma Aldrich;
Horseradish peroxidase-conjugated goat anti-mouse secondary antibody, Cat no. A8924 from Sigma;
Horseradish peroxidase-conjugated goat anti-rabbit secondary antibody, Cat no. P0448 from DAKO;
Anti-FLAG rabbit polyclonal, Cat no. F7425 from Sigma Aldrich;
5 or 10 nm gold-conjugated goat anti-rabbit IgG, Cat no. EM.GAR5/10, from BBI Solutions;
5 nm gold-conjugated goat anti-mouse IgG/IgM, Cat no. EM.GAF5, from BBI Solutions;
10 nm gold-conjugated goat anti-mouse IgG (H+L), Cat no. EM.GMHL10, from BBI Solutions.
5 nm gold-conjugated Protein-G, Cat no. AC-5-18-05, from Cytodiagnostics Inc.

Validation	All antibodies used in this study were validated by the manufacturer companies. Validation data/citations can be found on the manufacturers' websites by searching the antibody catalogue number provided both above and in the Materials & Methods section of our manuscript.

Eukaryotic cell lines

Policy information about cell lines

Cell line source(s)	HEK 293F/H (suspension variant of HEK 293T) from Invitrogen; Caspase-8-deficient Jurkat T cell line (clone I9.2 - gift from J Blenis Lab).
Authentication	Authenticated by ATCC Cell Line Authentication Service (STR Profile confirmed as ATCC human cell line HEK 293T: CRL-3216 and Caspase-8-deficient Jurkat T cell confirmed as human Jurkat T cell line, clone I9.2: ATCC CRL-2571).
Mycoplasma contamination	Tested and confirmed to be mycoplasma negative using McyoAlert Lonza every 12 months.
Commonly misidentified lines (See ICLAC register)	No commonly misidentified lines were used in this study.

Flow Cytometry

Plots

Confirm that:

- [x] The axis labels state the marker and fluorochrome used (e.g. CD4-FITC).
- [x] The axis scales are clearly visible. Include numbers along axes only for bottom left plot of group (a 'group' is an analysis of identical markers).
- [x] All plots are contour plots with outliers or pseudocolor plots.
- [x] A numerical value for number of cells or percentage (with statistics) is provided.

Methodology

Sample preparation	FACS analysis was used to quantify apoptotic cell death in cells co-transfected with catalytically Caspase-8 or Type I/II/III mutants. Caspase-8 deficient Jurkat cells were plated 24 h prior to transfection with a 5:1 ratio of Caspase-8 variant:EGFP, using PEI (Sigma). 24 h post-transfection, cells were treated with or without anti-CD95 (clone CH11; Cell Signalling) for 4 h and cell death in transfected cells specifically quantified by FACS using Annexin V-APC and Draq 7 co-labelling and gating of GFP-positive cells only.
Instrument	Data was acquired on a BD FACSCanto II cell analyser.
Software	Data analysis was performed using BD FACS Diva, version 8.0.1.
Cell population abundance	No flow-based sorting was performed.
Gating strategy	The FSC/SSC gates confirmed the single cell population and GFP-gating indicated GFP-transfected cells. GFP-transfected cells were then selectively analysed for Annexin V-APC and Draq 7 labelling. Annexin V-APC labelling alone was a marker of early apoptotic cells, while double-labelled Annexin V-APC/Draq 7 cells represented late apoptotic cells. Double negative cells represented viable cells.

- [x] Tick this box to confirm that a figure exemplifying the gating strategy is provided in the Supplementary Information.
